# Short-Term Dynamic and Local Epidemiological Trends in the South American HIV-1B Epidemic

**DOI:** 10.1371/journal.pone.0156712

**Published:** 2016-06-03

**Authors:** Dennis Maletich Junqueira, Rubia Marília de Medeiros, Tiago Gräf, Sabrina Esteves de Matos Almeida

**Affiliations:** 1 Centro Universitário Ritter dos Reis—Uniritter, Departamento de Ciências da Saúde, Porto Alegre, RS, Brazil; 2 Programa de Pós-Graduação em Genética e Biologia Molecular, Universidade Federal do Rio Grande do Sul (UFRGS), Porto Alegre, RS, Brazil; 3 Centro de Desenvolvimento Científico e Tecnológico (CDCT), Fundação Estadual de Produção e Pesquisa em Saúde (FEPPS), Porto Alegre, RS, Brazil; 4 Programa de Pós-graduação em Biotecnologia e Biociências, Universidade Federal de Santa Catarina, Florianópolis, SC, Brazil; 5 Instituto de Ciências da Saúde, Universidade FEEVALE, Novo Hamburgo, RS, Brazil; University of Malaya, MALAYSIA

## Abstract

The human displacement and sexual behavior are the main factors driving the HIV-1 pandemic to the current profile. The intrinsic structure of the HIV transmission among different individuals has valuable importance for the understanding of the epidemic and for the public health response. The aim of this study was to characterize the HIV-1 subtype B (HIV-1B) epidemic in South America through the identification of transmission links and infer trends about geographical patterns and median time of transmission between individuals. Sequences of the protease and reverse transcriptase coding regions from 4,810 individuals were selected from GenBank. Maximum likelihood phylogenies were inferred and submitted to ClusterPicker to identify transmission links. Bayesian analyses were applied only for clusters including ≥5 dated samples in order to estimate the median maximum inter-transmission interval. This study analyzed sequences sampled from 12 South American countries, from individuals of different exposure categories, under different antiretroviral profiles, and from a wide period of time (1989–2013). Continentally, Brazil, Argentina and Venezuela were revealed important sites for the spread of HIV-1B among countries inside South America. Of note, from all the clusters identified about 70% of the HIV-1B infections are primarily occurring among individuals living in the same geographic region. In addition, these transmissions seem to occur early after the infection of an individual, taking in average 2.39 years (95% CI 1.48–3.30) to succeed. Homosexual/Bisexual individuals transmit the virus as quickly as almost half time of that estimated for the general population sampled here. Public health services can be broadly benefitted from this kind of information whether to focus on specific programs of response to the epidemic whether as guiding of prevention campaigns to specific risk groups.

## Introduction

Today, nearly one million individuals are infected by HIV-1 in South America and approximately 35,000 individuals die per year victims of the symptoms of AIDS [1]. A quick answer to the high mortality rate, morbidity and transmission related to HIV in certain specific regions of the South America may require a broad and efficient collection of data on the virus and the host [2]. In this context, phylogenetic analyses have been used to investigate the origin, spread and transmission of HIV between individuals and can be a powerful method in the understanding of the social, demographic and geographical issues in the epidemic [3–11].

Transmission clusters are identified here as phylogenetic inferences of the transmission links between different HIV-infected individuals. Through the genetic and evolutionary relationship these epidemiological chains allow the inference of social contacts between individuals and provide a way to reconstruct the main transmission links involved in the spread of the virus in a certain place [6,12,13]. These findings improves the correlation of local HIV epidemic with transmission pathway, drug resistance, risk behavior and cluster size and may influence the direction focus of public campaigns to specific populations aiming to reduce the rate of transmissions and consequently delaying the increase in the number of new infection cases [14]. While several countries in Europe, Asia, Africa, Australia and North America have already impactful information about the transmission between individuals, South American countries are clearly deficient for this kind of information [6,15–25]. Addressing the specific issues within local epidemics is crucial to a greatly improvement in the HIV-1 epidemic [2].

Several studies have exploited the HIV-1 high rates of mutation and its intrinsic rapid evolution to understand the spread of the virus in a certain population of a specific place [4,26–28]. Here we used phylogenetic analysis to identify and characterize the transmission links y and characterize among HIV-1 South American sequences available in Genbank. As South America has an epidemic primarily based on HIV-1 subtype B (HIV-1B), all analysis were performed using *pol* sequences of this subtype.

## Methods

### Alignments

All available South American HIV-1 sequences from *pol* gene including the protease and partial segment of the reverse transcriptase (nucleotides 2253–3252 relative to strain HXB2) were selected from the Los Alamos HIV Sequence Database (http://www.hiv.lanl.gov/). Additionally, PubMed (http://www.ncbi.nlm.nih.gov/pubmed) was used to consult all studies of HIV-1 conducted in South American countries through the use of the search key “HIV-1 AND country” (where “country” was substituted for the name of each South American country) to select sequences deposited in GenBank (http://www.ncbi.nlm.nih.gov/nucleotide/) but not included in the Los Alamos Database. When available, geographical, demographic and clinical information were also retrieved from Los Alamos or directly from the study describing the sequence.

All sequences were submitted to REGA Subtyping Tool v2.0 [29], and RIP 3.0 [30] to confirm the subtype. Sequences presenting discordances in the subtype assignment were submitted to bootscanning analysis using Simplot 3.5.1 [31]. Final alignment were generated using MUSCLE [32] and it excluded sequences presenting premature stop codons, replicates from the same patient, and intersubtype recombinants. Four sequences of subtype D were used as outgroup.

In order to compare the influence of convergent evolution of resistance mutations on the transmission cluster detection, two versions of the final alignment were built. A set including complete sequences (complete set, 1000bp), and a codon-stripped dataset (codon-stripped set) in which the main sites associated with major antiretroviral drug resistance for protease and reverse transcriptase were excluded, resulting in a 901bp alignment [33]. All alignments are available from the authors upon request.

### Identification of Transmission Clusters

Maximum Likelihood (ML) phylogenies were inferred in RAxML [34] on the CIPRES Science Gateway [35] incorporating the best-fitted nucleotide substitution model (GTR+I+Γ) as determined in MEGA6 [36]. The approximate likelihood-ratio test (aLRT) based on Shimodaira-Hasegawa-like procedure were used to assess confidence in topology [37].

Transmission links were identified by using Cluster Picker [38] with a SH-aLRT support threshold of ≥90. Different maximum pairwise genetic distances (1.0%, 1.5%, 2.0%, 2.5%, 3.0%, 3.5%, 4.0%, 4.5%, 5.0%, 5.5%, 6.0%, 6.5%, 7.0%, and 7.5%) within the clusters were evaluated and transmission cases previously known (09 transmission pairs and 01 transmission triads) were used as controls for the genetic distance evaluation [39–41]. Resultant transmission clusters were classified in local (clusters involving sequences sampled in the same state for Brazilian sequences or in the same country for non-Brazilian sequences), interstate (clusters involving sequences sampled in different states within Brazil), or international types (clusters involving sequences sampled in different countries) according to the sampling region of the sequences involved in the cluster.

### Time-scaled phylogenies

In order to estimate the median time of HIV-1 transmission between individuals, dated phylogenies were reconstructed using a Bayesian MCMC method implemented in BEAST v1.8 [6,22,42]. Only clusters including ≥5 dated samples were selected for further analysis.

The Bayesian analyses assumed an uncorrelated lognormal relaxed molecular clock under the GTR+I+Γ nucleotide substitution model and five different demographic models were tested: constant population size, expansion, exponential growth, logistic growth, and the non-parametric Bayesian skyline plot. The best fitted model were evaluated trough marginal likelihood estimation. Previous estimates of the evolutionary rate for subtype B were used as normal mean prior [43]. The MCMC chain were run for 5.0 x 10^8^ chain steps and the convergence was evaluated in TRACER v1.5 excluding an initial 10% for burn-in [44]. Maximum clade credibility trees (MCC) were summarized using TreeAnnotator v1.8.0 in BEAST package after 50% of the burn-in was discarded and the resulting tree was visualized with FigTree v.1.4.2.

### Resistance Mutation Analysis

Complete sequences alignment were submitted to Calibrated Population Resistance tool (CPR) to detect the presence of surveillance Drug Resistance Mutations (DRM) [45]. Clusters that included sequences presenting the same antiretroviral drug resistance mutations were used to compose a new dataset in order to better understand the transmission of drug resistance among different individuals within a transmission group. The new dataset were submitted to maximum likelihood phylogeny inference in PhyML incorporating the best-fitted nucleotide substitution model (GTR+I+Γ). The resulting maximum likelihood tree were used to reconstruct the ancestral nucleotide sequence of each cluster using the FastML web server software [46].

### Statistical Analysis

Statistical comparisons between datasets (complete *versus* codon-stripped dataset, including geographic distribution, resistance mutation analysis and time between infections) were made using Pearson’s χ^2^-test and Fisher’s exact test when appropriate. Statistical analyses were performed using WinPepi v.11.22 and the significance level was set at P < 0.05.

## Results

### Dataset

**Fig 1:**
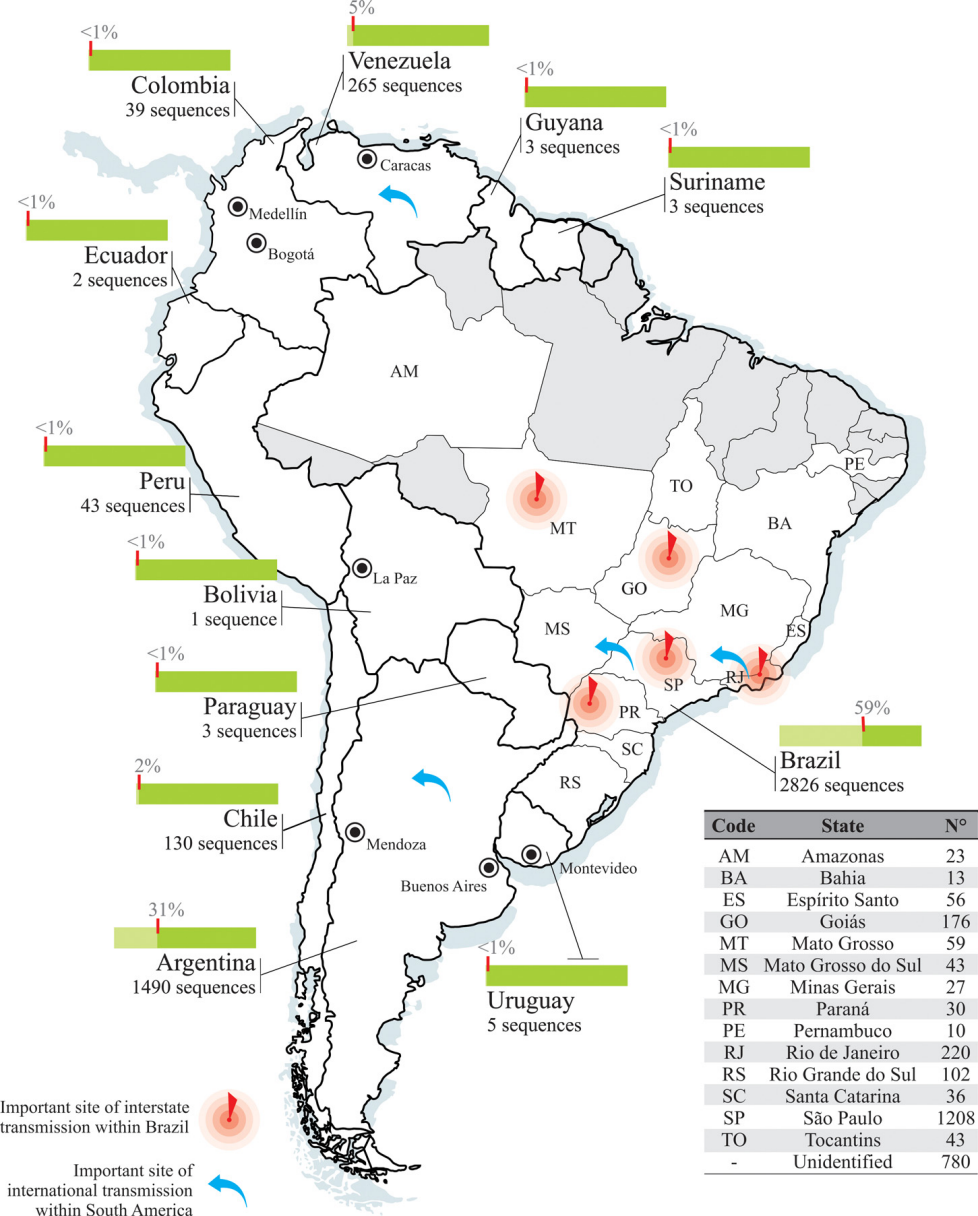
Geographic distribution and proportion of HIV-1 subtype B *pol* sequences from South American countries. Map shows locations of the HIV-1 subtype B sequences included in the dataset. The proportion (green bars) and the total number of sequences analyzed from each location is indicated. A compilation of all sequences from the Brazilian set and its respective state of sampling is indicated at the table included in the figure. Black dots indicate the cities sampled in this study. Sequences from Venezuela were sampled at Caracas (n = 213), from Colombia were sampled at Medellín (n = 32) and Bogotá (n = 7), the unique sequence from Bolivia was sampled at La Paz, sequences from Argentina were sampled at Mendoza (n = 2) and Buenos Aires (n = 1238), and sequences from Uruguay were sampled at Montevideo. For the rest of the countries the sequences had no identification of sampling region. Gray-shaded areas indicate regions not included in this study.

Among 7,600 *pol* sequences available in public databases, 4,810 HIV-1 subtype B isolates from South American countries were selected to set up a dataset for the identification of transmission links (S1 Table). Twelve of the 13 South American countries were represented in the 26 different regions included in this study. Due to the large area occupied by Brazil in South America, samples isolated in this country were identified by specific state of sampling (Fig 1).

Brazil was by far the most represented country in our dataset, exhibiting 2,826 sequences (58.7%) from 14 geographical states (Fig 1). Due to the unavailability of sampling region information, 780 sequences from Brazil could not have an attribution for the sampling state. Argentina was the second best represented country in our dataset with 1,490 sequences. Bolivia, Ecuador, Guyana, Paraguay, Suriname, and Uruguay were misrepresented regions with less than 10 sequences per country.

### Alignments

Traditionally, transmission clusters studies have used datasets including codon-stripped sequences from which codons associated with major resistance in protease and reverse transcriptase are removed [6,16,18,20,38]. As one of the goals of this study was to evaluate the influence and transmission of drug resistance mutations in transmission clusters, we constructed two different datasets (complete and codon-stripped datasets) to test whether the analysis of the complete set could distort the results. Despite the differences in the composition of sequences inside the transmission clusters, all results obtained in this study are relatively similar for both datasets, including geographic distribution, resistance mutation and time between infections (S1 Fig, S2 and S4 Tables).

### Within-cluster maximum genetic distance thresholds

**Fig 2:**
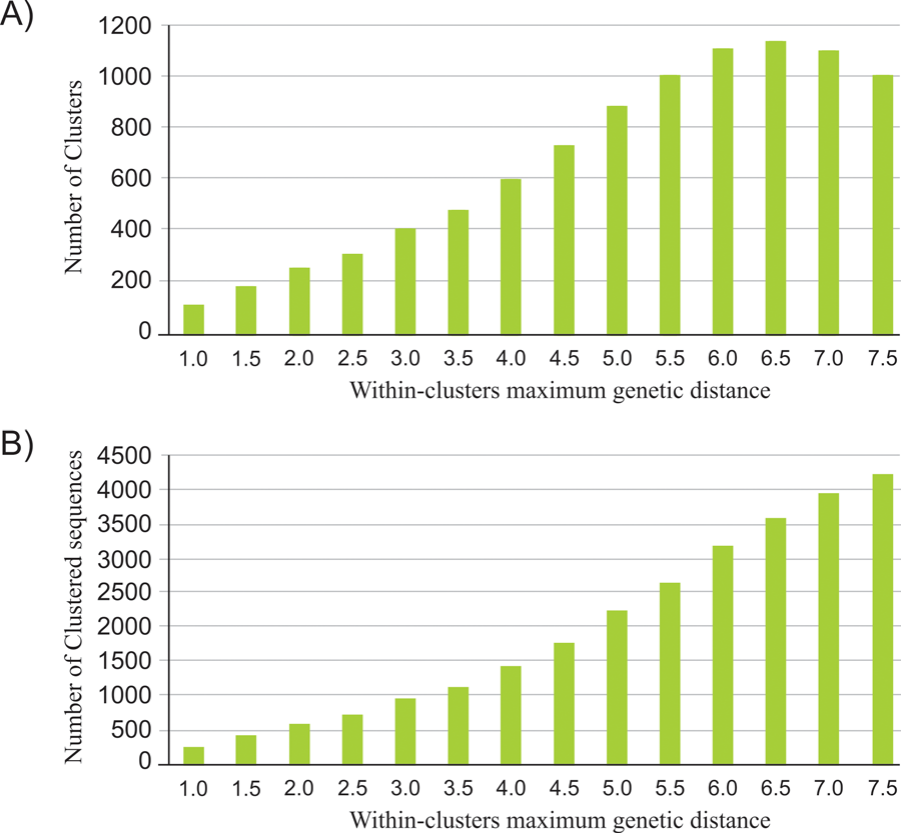
Number of transmission clusters and clustered sequences among 4,810 HIV-1 Subtype B codon-stripped *pol* sequences from South America. (A) Number of transmission clusters identified using Cluster Picker with a SH-aLRT support threshold of ≥90 and under different within-maximum genetic distances. (B) Number of clustered sequences under different within-cluster genetic distances.

We first evaluated the effect of different maximum genetic distances on cluster identification among HIV-1 sequences from South America. The branch support threshold was fixed at 90 (SH-aLRT) with a within-cluster maximum genetic distance varying between 1.5% and 7.5% (using intervals of 0.5%). The number of clusters detected increased with the genetic distance threshold, reaching a maximum at 6.5% (Fig 2 and S1 Fig). At genetic distances of 7.0% and 7.5%, the number of detected clusters was smaller than for 6.5%. Beyond the within-cluster maximum genetic distance of 4.5%, the proportion of new transmission clusters gradually decreases.

In the other way, the proportion of sequences included in the clusters enlarged with the increase in the genetic distance threshold (Fig 2 and S1 Fig). At the genetic distance of 7.5%, more than 80% of the sequences analyzed were included in transmission clusters. These results indicate that as the maximum genetic distance cut-off is relaxed, more sequences are being added to the clusters.

In order to understand the HIV-1 epidemic patterns, transmission links were identified using Cluster Picker in a within-cluster maximum genetic distance of 4.5%. This cut-off effectively detected all transmission links included as controls in this study and it was previously used in other studies [6,22,38].

### HIV-1 Subtype B transmission links

A total of 729 transmission clusters were identified in a SH-aLRT support threshold of ≥90 and a maximum genetic distance of 4.5% (Table 1 and S2 Table). Around 71% of the transmissions links identified here are clustering sequences from the same geographical region (Table 1 and S2 Table). As expected, transmissions links including individuals from different states (within Brazil) or countries were detected to a lesser extent (8,6% and 5,5% respectively).

**Table 1:** Geographical type of HIV-1 Subtype B transmissions among clusters identified within South America for the codon-stripped dataset (901bp).

Clustered Individuals	Geographical Type of Transmission	Number of Clusters Identified	%
**2**	Local Transmission*	392	70,6
	Interstate Transmission (Brazil)	44	7,93
	International Transmission	31	5,59
	Unidentified	88	15,9
**3**	Local Transmission*	81	68,6
	Interstate Transmission (Brazil)	13	11
	International Transmission	6	5,08
	Unidentified	18	15,3
**4**	Local Transmission*	28	73,7
	Interstate Transmission (Brazil)	4	10,5
	International Transmission	2	5,26
	Unidentified	4	10,5
**5**	Local Transmission*	9	81,8
	Interstate Transmission (Brazil)	1	9,09
	International Transmission	1	9,09
**6**	Local Transmission*	1	100
**7**	Local Transmission*	1	50
	Interstate Transmission (Brazil)	1	50
**≥8**	Local Transmission*	4	100
**Total**		729	-

* Transmission Clusters involving sequences sampled in the same state for Brazilian sequences or in the same country for non-Brazilian sequences

Brazil, Argentina and Venezuela seemed to play an important role in the HIV-1 subtype B epidemic inside the continent, presenting the higher number of intercountry connections (S3 Table). In Brazil, the states of São Paulo, Goiás, Rio de Janeiro, Mato Grosso, and Paraná posed as potential sites for the international link within the HIV-1B epidemic in South America as seen by the number of links with other sequences (S3 Table).

Four transmission clusters linked more than seven individuals (Table 1). It is important to highlight that two of these larger clusters were associated exclusively to MSM transmission, and one included both MSM and heterosexual individuals. We verified that approximately 41% of all clusters including more than 4 individuals were made up entirely of MSM individuals.

### Dating Transmission events

As each tip of the tree represents a single patient, the internal nodes represent the most recent common ancestors of these infections and include at least one transmission event. Therefore, the internode intervals are used to estimate the maximum inter-transmission times between patients scaled by calendar years. It is important to highlight that these time intervals are maximum estimates and the inclusion of more individuals in the cluster would lead to shorter average transmission intervals.

We determined the average period in which the clustered transmissions occurred for all groups including ≥5 individuals (82 sequences, 14 clusters) under a BSP demographic model (Table 2 and S4 Table). Analysis of the overall distribution of the internode intervals in South America revealed a median of 1.98 years (95% confidence interval [CI] 1.04–2.92). The great majority of the transmission clusters analyzed with the Bayesian approach were from Brazil allowing the determination of a country-specific average transmission interval of 2.65 years (95% CI 1.3–4.0).

**Table 2:** Average time of HIV-1 subtype B transmission among South American individuals for the codon-stripped dataset (901bp).

Cluster	Taxa	Geographical Type	Exposure Category	Median Internode Intervals (years)
**1**	4	Local	-	6.410
**2**	7	Interstate	HET/MSM	4,468
**3**	5	Local	MSM	0.411
**4**	5	Local	MSM	0.302
**5**	4	Local	HET	2.247
**6**	6	Local	MSM	0.394
**7**	5	Local	MSM	1.623
**8**	5	International	-	1.117
**9**	5	Local	-	1.540
**10**	5	Local	MSM	0.757
**11**	13	Local	MSM	1.596
**12**	6	Local	-	3.361
**13**	5	Local	MSM	0.517
**14**	7	Local	HET/MSM	2.970
** **	**Median 1.98 (95% CI 1.04–2.92)**			

Abbreviations: HET: Heterosexual individual, MSM: men who have sex with men individual

Clusters primarily composed of MSM individuals were separately analyzed in order to calculate the average time of transmission between MSM individuals in South America. For MSM clusters, we found a median time of transmission between individuals of 0.8 years (7 clusters, 45 sequences, 95% CI 0–2.16).

### Resistance Mutation Analysis

The overall DRM prevalence of the samples included in clusters was 44.1% (720/1633; 95% CI 41.7–46.5; Table 3). The prevalence of sequences presenting only drug resistance mutations against protease inhibitors (PI) was 1.53% (25/1633; 95% CI 0.94–2.13), against nucleoside RT inhibitors (NRTI) was 6.43% (105/1633; 95% CI 5.24–7.62), and against non-NRTI (NNRTI) was 3.74% (61/1633; 95% CI 2.82–4.66). In addition, 170 sequences (10.4%; 95% CI 8.93–11.89) presented mutations that conferred resistance to the three classes of antiretroviral drugs, 171 sequences (10.5%; 95% CI 8.99–11.96) presented resistance mutations against PI and NRTI, 10 sequences (0.6%; 95% CI 0.23–0.99) presented PI and NNRTI-resistance mutations, and 178 sequences (10.9%; 95% CI 9.39–12.41) were identified as carrying resistance mutations against NRTI and NNRTI (Table 3). Within protease, L90M (17.5%) was the most frequent mutation, followed by M46I (13%), I54V (9.1%), V82A (9%), and I84V (9%) (S5 Table). The revertants at RT position 184 (M184V) were the most prevalent (18%), followed by M41L (15%), T215Y (13%), L210W (9.7%), and D67N (9.6%) (S6 and S7 Tables). We found 400 clusters presenting samples with DRM.

**Table 3:** Drug resistance mutations identified among 4,810 sequences clustered or not clustered in transmission clusters within South America.

Drug Resistance Mutation (DRM)	Number of Sequences presenting DRM (n = 4810)	Clustered Sequences (n = 1633)		Not Clustered Sequences (n = 3177)	
		N	%	N	%
PI	64	25	1.53	39	1,23
NRTI	346	105	6.43	241	7,59
NNRTI	180	61	3.74	119	3,75
PI + NRTI	751	171	10.5	580	18,3
PI + NNRTI	31	10	0.61	21	0,66
NRTI + NNRTI	788	178	10.9	610	19,2
PI + NRTI + NNRTI	847	170	10.4	677	21,3
**Total**	**3007**	**720**	**44.1**	**2287**	**72**

Abbreviations: NRTI: nucleoside reverse transcriptase inhibitor, NNRTI: non- nucleoside reverse transcriptase inhibitor, PI: protease inhibitor

We also analyzed the prevalence of DRM in the set of sequences not included in any cluster in order to understand the influence of the clustering in the transmission of drug resistance mutations. From 3,177 sequences not included in transmission clusters, 2,287 samples (72%; 95% CI 70.42–73.55) were identified as carrying drug resistance mutations (Table 3). Due to the high prevalence of resistance mutation in this set, we analyzed all sequences by searching for the patient antiretroviral therapy status directly from the study describing each sequence. The great majority of the sequences not included in any cluster (1,537 samples, 48.4%) could not have its antiretroviral status attributed (S8 Table). The remaining 1,640 sequences were categorized in three groups: (i) sequences sampled from patients failing antiretroviral therapy (1271 samples, 40%), (ii) sequences from treatment-naive patients (236 samples, 7.4%), and (iii) sequences from antiretroviral treated individuals (133 samples, 4.2%; S8 Table). Statistical comparison revealed that clustered sequences included more samples from treatment-naive patients and fewer samples from patients failing antiretroviral therapy (P<0,001) than no clustered sequences. Analysis of the most frequent DRM in protease and reverse transcriptase genes revealed a similar pattern between the sequences not included in any cluster and those included in clusters (S5–S7 Tables).

In addition, we examined the ancestral states at all sites associated with drug resistance for 28 clusters including more than three individuals (129 total individuals) (S9 Table). In all cases, the reconstructed ancestral sequence of the transmission cluster harbored the same drug resistance mutation as the sequences within the cluster. However, the selection of the clusters to evaluate the ancestral amino acids may create a bias in this analysis since we only chose clusters that included more than three sequences presenting the same resistance mutation.

## Discussion

The heterogeneity of the dataset used in this study brings a new challenge to the phylodynamic field. Due to the inexistence of a consensus methodology to identify transmission clusters, here we tested the effect of different maximum genetic distances on cluster identification among HIV-1B samples from South America. We observed that from the within-cluster maximum genetic distance of 4.5% the proportion of new transmission clusters identified by Cluster Picker gradually decreases. This result was expected since the inclusion of more sequences in each cluster and/or the combination of smaller ones will allow the detection of increased size clusters. Based on this result we judged the within-cluster maximum genetic distance of 4.5% to be appropriate in detecting transmission clusters [47].

At a genetic distance threshold of 4.5%, more than 720 individuals had a link to at least one other subject (Table 1). It is important to note that the vast majority of these transmission links (71%) included individuals living in the same geographic region. These results may suggest that HIV-1B infections primarily occur among individuals living in the same geographic region. This estimative represents the minimum proportion since several sequences in our dataset presented missing data for geographic origin. If extrapolated, this result may indicate that in average 71% of the epidemic from a particular location in South America can be explained only by local epidemiological trends. We also observed a tendency relating geographical distance to epidemiological connection most likely linking individuals from closest states or countries. These estimates might be a reflection of the poor infrastructure integration relative to accessibility to and among urban centers in the South America. The isolation of countries and cities in the region therefore might be viewed as potential factors intensifying the local trends in the HIV-1B epidemic. Mir et al. (2015) in a study including 6789 HIV-1B sequences from seven different Latin American countries found a much greater geographic compartmentalization of the HIV-1B epidemic in Latin American and Caribbean regions than in Europe [25]. The study supports that the epidemic is mainly driven by transmissions within countries and among neighboring countries than transmissions among countries without a common border. These results most likely reflect an intraregional difference in human mobility and spatial accessibility in Latin American countries.

In relation to the Brazilian interstate links, the geographic distribution revealed that Goiás, São Paulo, Rio de Janeiro, Paraná, and Mato Grosso are important sites of transmission within this country (Fig 1 and S4 Table). Despite the increased number of samples from São Paulo, Rio de Janeiro, and Goiás in our dataset, these results support such conclusions because we chose to identify transmission clusters based in two assumptions: (i) the support threshold of >90 and (ii) the within-clade maximum genetic distance of 4.5%. These assumptions do not prevent the increase in probability of link one sequence of a densely sampled state to another of different state in the same clade; however, it avoids the identification of such clade as a transmission cluster. In addition, demographic data from the Official Brazilian Demographic Data Center—IBGE (http://www.ibge.gov.br/home/) show that these states receive the highest number of internal migrants in the country corroborating the idea that migratory relationship between different states creates potential networks to the diffusion of HIV in the human population (IBGE, 2010). Moreover, São Paulo and Rio de Janeiro are important sites for an international connection of the HIV-1B epidemic within Brazil (Fig 1). Demographic data can support our results since São Paulo and Rio de Janeiro are among the states in Brazil to receive the highest number of immigrants from other countries (http://www.ibge.gov.br/home/).

Our results revealed that Brazil, Argentina and Venezuela are important sites for the spread of HIV-1B within South America. Although most South American countries are misrepresented regions in our dataset, creating a potential sampling bias to conclude about their relative importance in the continental spread of HIV-1B, our findings are in agreement with migratory data revealing that Brazil, Argentina and Venezuela are primary points for regional migration (http://www.iom.int/world-migration). Supposedly, the spread of HIV-1 in South America may be associated to the migratory pathways established by the human population in search of economic or social change. These results highlight the importance of association between demographic data and epidemiological information to construct a full scenario of the current epidemic in a certain region [48].

We also analyzed the dynamic of dissemination within clusters to infer the time between transmissions from an individual to another. Bayesian analysis revealed an average time period of HIV-1B transmissions within South America of 1.98 years (Table 2). Our results propose a short-term dynamic of the epidemic among HIV infected individuals in which after approximately 2 years being infected patients will transmit the virus to other individual. A previous study analyzing heterosexual transmission in a group of 11,071 individuals from the United Kingdom epidemic of HIV-1 non-B subtypes found similar results [22]. The agreement with our findings may indicate a similar pattern in the transmission of HIV-1 irrespective of the population analyzed. This information is of utmost importance to understand the dynamics of infection and suggest that the early stage of infection potentially influence the onward spread of HIV [49,50].

The identification of clusters including only MSM individuals in our results allowed the estimation of an average time of transmission in this group of 0.8 years. We observed that MSM individuals transmit the virus to another subject in almost half time of that for the general population sampled here. The short time interval between transmissions can be explained by the stage of infection since within the first year after transmission, HIV infection in MSM individuals are eight times as infectious as during chronic phase [49]. Additionally, we encountered a larger number of transmission clusters (41%) including more than four individuals comprising only MSM individuals. It suggests a difference in the dynamics of the epidemics in different risk groups most likely reflecting social and sexual behavior of the human population [22]. These results should be taken as a warning data to the public health services since the transmission of the virus within the group is faster than for others groups and that in most countries the population of MSM individuals is still the most affected by HIV infection [51,52]. Prevention measures and information campaigns could absorb this information to guide the reallocation of resources and manage new investments to focus in populations in which the epidemic is concentrated.

An elevated proportion (62%) of sequences presented drug resistance mutation (DRM) in our dataset (Table 3 and S8 Table). The vast majority of sequences assigned for a therapy status were sampled from patients failing antiretroviral therapy (37%) which might explain the increased prevalence of drug resistance mutations in our sample [53–55]. When stratifying the data, we found lower prevalence of DRM in the set of sequences included in transmission clusters (Table 3). This result is consistent with the decreased replication fitness of viruses harboring DRM [11,56,57]. In addition, statistical comparisons based on Pearson’s χ2-test revealed an increased number of sequences from treatment-naive patients included in the linked transmissions suggesting that naive individuals might be transmitting the virus in higher rates than treated or failing antiretroviral individuals. The reasons for this pattern may be related to the lack of knowledge about HIV status and the high viral load characteristic of these untreated patients.

The ancestral reconstructions within transmission clusters revealed a tendency to the circulation of transmitted drug resistance mutations in South America since we found all inferred ancestral sequences harboring the most prevalent mutation within each cluster (S7 Table). In addition, among the resistance mutations found in this study, M184V (found in 38% of the sequences) and M41L (found in 28.5% of the sequences) were the most frequent mutations found in our samples (S2 and S4 Tables). Both mutations reflect resistance to NNRTI drugs e are described as the most common in patients failing antiretroviral therapy [58].

## Conclusion

Our results show that the HIV-1B epidemic is largely influenced by regional trends and suggest a higher probability of HIV transmission between individuals from the same geographic origin. This compartmentalized analysis of the epidemic (by states or countries) and the consequent influence of local, interstate or international transmissions had never been defined for the HIV-1 epidemic in South America. In addition, we propose a short-term dynamics within clusters in which the mean time of transmissions is two years. Information regarding the regional dynamic of HIV infections allied to statistical methods may contribute to the understanding on dissemination and expansion of new variants in certain places, including the entry of new subtypes and the circulation of drug resistant strains.

## Supporting Information

S1 Fig Number of transmission clusters and clustered sequences for the complete and codon-stripped datasets among 4,810 HIV-1 Subtype B *pol* sequences from South America. (A) Absolute number of transmission clusters identified with a SH-aLRT support threshold of ≥90. (B) Absolute number of clustered sequences under different within-cluster genetic distances.
(TIF)

S1 Table GenBank accession number and country of origin of 4,810 HIV-1 Subtype B sequences isolated in South America. (DOCX)

S2 Table Geographical type of HIV-1 Subtype B transmissions among clusters identified within South America for the complete dataset (1000bp). (DOCX)

S3 Table Number of links identified for the states within Brazil involved in interstate transmissions and for the countries involved in international transmissions at South America (including results from transmission pairs and transmission clusters). (DOCX)

S4 Table Average time of HIV-1 subtype B transmission among South American individuals for the complete dataset (1000bp). (DOCX)

S5 Table Amino acid substitutions in HIV-1 Protease gene related to drug resistance to protease inhibitors (PI) identified among 4,810 sequences clustered or not clustered in transmission clusters within South America. (DOCX)

S6 Table Amino acid substitutions in HIV-1 Reverse transcriptase gene related to drug resistance to nucleoside reverse transcriptase inhibitors (NRTI) identified among 4,810 sequences clustered or not clustered in transmission clusters within South America. (DOCX)

S7 Table Amino acid substitutions in HIV-1 reverse transcriptase gene related to drug resistance to non-nucleoside reverse transcriptase inhibitors (NNRTI) identified among 4,810 sequences clustered or not clustered in transmission clusters within South America. (DOCX)

S8 Table Antiretroviral therapy (ART) status of the patients and clustering behavior of the respective sequences. (DOCX)

S9 Table Ancestral reconstruction analysis of the sequences included in clusters and presenting resistance drug mutations. (DOCX)
