# Supplementary figures and images for: Short-Term Dynamic and Local Epidemiological Trends in the South American HIV-1B Epidemic
Source: PLoS One. 2016 Jun 3;11(6):e0156712. doi: 10.1371/journal.pone.0156712 (PMC4892525; doi:10.1371/journal.pone.0156712)

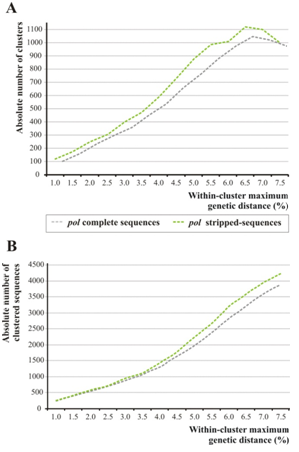

Supplement: S1 Fig — (A) Absolute number of transmission clusters identified with a SH-aLRT support threshold of ≥90. (B) Absolute number of clustered sequences under different within-cluster genetic distances. (TIF) [file pone.0156712.s001.tif]
