# Supplementary material for: Short-Term Dynamic and Local Epidemiological Trends in the South American HIV-1B Epidemic
Source: PLoS One. 2016 Jun 3;11(6):e0156712. doi: 10.1371/journal.pone.0156712 (PMC4892525; doi:10.1371/journal.pone.0156712)
Supplement: S1 Table — (DOCX) [file pone.0156712.s002.docx]

**S1 Table. GenBank accession number and country of origin of 4,810 HIV-1 Subtype B sequences isolated in South America.**

| **Sequence**  **Name** | **GenBank Accession**  **Number** | **Isolation**  **Country** | **Isolation**  **Year** |
| --- | --- | --- | --- |
| 1AB10H08 | JF342274 | Brazil | 2008 |
| 1AD20H08 | JF342276 | Brazil | 2008 |
| 1AG10H08 | JF342279 | Brazil | 2008 |
| 1AH20H08 | JF342280 | Brazil | 2008 |
| 1AI20H08 | JF342283 | Brazil | 2008 |
| 1AJ10H08 | JF342281 | Brazil | 2008 |
| 1AK20H08 | JF342284 | Brazil | 2008 |
| 1AL20H08 | JF342284 | Brazil | 2008 |
| 1AM10H08 | JF342285 | Brazil | 2008 |
| 1AP10H08 | JF342288 | Brazil | 2008 |
| 1AQ20H08 | JF342289 | Brazil | 2008 |
| 1AX10H08 | JF342296 | Brazil | 2008 |
| 1AY10H08 | JF342297 | Brazil | 2008 |
| 1BD10H08 | JF342302 | Brazil | 2008 |
| 1BE10H08 | JF342303 | Brazil | 2008 |
| 1BF20H08 | JF342304 | Brazil | 2008 |
| 1BG10H08 | JF342305 | Brazil | 2008 |
| 1BI10H08 | JF342307 | Brazil | 2008 |
| 1BJ10H08 | JF342308 | Brazil | 2008 |
| 1BK20H08 | JF342309 | Brazil | 2008 |
| 1BM20H08 | JF342311 | Brazil | 2008 |
| 1BN10H10 | JF342312 | Brazil | 2010 |
| 1BT10H10 | JF342319 | Brazil | 2010 |
| 1BW10H10 | JF342321 | Brazil | 2010 |
| 1BX00C02 | EU340706 | Brazil | 2002 |
| 1BY00C02 | EU340707 | Brazil | 2002 |
| 1CF12C05 | EU340714 | Brazil | 2005 |
| 1CI12C05 | EU340717 | Brazil | 2005 |
| 1CJ12C05 | EU340718 | Brazil | 2005 |
| 1CK11C05 | EU340719 | Brazil | 2005 |
| 1CL13C05 | EU340720 | Brazil | 2005 |
| 1CM12C05 | EU340721 | Brazil | 2005 |
| 1CO11C06 | EU340723 | Brazil | 2006 |
| 1CP11C06 | EU340724 | Brazil | 2006 |
| 1CR11C06 | EU340726 | Brazil | 2006 |
| 1CT11C06 | EU340728 | Brazil | 2006 |
| 1CW00C06 | EU340731 | Brazil | 2006 |
| 1CX12C06 | EU340732 | Brazil | 2006 |
| 1CY22C06 | EU340733 | Brazil | 2006 |
| 1CZ11C06 | EU340734 | Brazil | 2006 |
| 1DA22C06 | EU340735 | Brazil | 2006 |
| 1DC11C06 | EU340737 | Brazil | 2006 |
| 1DH00C06 | EU340742 | Brazil | 2006 |
| 1DK00C06 | EU340745 | Brazil | 2006 |
| 1DN00C06 | EU340748 | Brazil | 2006 |
| 1DP12C06 | EU340750 | Brazil | 2006 |
| 1DR12C06 | EU340752 | Brazil | 2006 |
| 1DS11C06 | EU340753 | Brazil | 2006 |
| 1DU00F97 | AF112890 | Brazil | 1997 |
| 1DX00F97 | AF112893 | Brazil | 1997 |
| 1DY00F97 | AF112894 | Brazil | 1997 |
| 1DZ00F97 | AF112895 | Brazil | 1997 |
| 1EA00F97 | AF112896 | Brazil | 1997 |
| 1ED00F97 | AF112899 | Brazil | 1997 |
| 1EE00F97 | AF112900 | Brazil | 1997 |
| 1EF00F97 | AF112901 | Brazil | 1997 |
| 1EG00F97 | AF112902 | Brazil | 1997 |
| 1EJ00F97 | AF112905 | Brazil | 1997 |
| 1EK00F97 | AF112906 | Brazil | 1997 |
| 1EL00F97 | AF112907 | Brazil | 1997 |
| 1EM00F97 | AF112908 | Brazil | 1997 |
| 1EO00F97 | AF112910 | Brazil | 1997 |
| 1EQ00F97 | AF112912 | Brazil | 1997 |
| 1ER00F97 | AF112913 | Brazil | 1997 |
| 1ET00F97 | AF112915 | Brazil | 1997 |
| 1EU00F97 | AF112916 | Brazil | 1997 |
| 1EV00F97 | AF112917 | Brazil | 1997 |
| 1EW00F97 | AF112918 | Brazil | 1997 |
| 1EX00F97 | AF112919 | Brazil | 1997 |
| 1EY00F97 | AF112920 | Brazil | 1997 |
| 1EZ00F97 | AF112921 | Brazil | 1997 |
| 1FA00F97 | AF112922 | Brazil | 1997 |
| 1FB00F97 | AF112923 | Brazil | 1997 |
| 1FC00F97 | AF112924 | Brazil | 1997 |
| 1FD00F97 | AF112925 | Brazil | 1997 |
| 1FE00F97 | AF112926 | Brazil | 1997 |
| 1FG00F97 | AF112928 | Brazil | 1997 |
| 1FH00F97 | AF112929 | Brazil | 1997 |
| 1FJ00F97 | AF112931 | Brazil | 1997 |
| 1FK00F97 | AF112932 | Brazil | 1997 |
| 1FL00F97 | AF112933 | Brazil | 1997 |
| 1FM00F97 | AF112934 | Brazil | 1997 |
| 1FN00F97 | AF112935 | Brazil | 1997 |
| 1FO00F97 | AF112936 | Brazil | 1997 |
| 1FQ00F97 | AF112938 | Brazil | 1997 |
| 1FR00F97 | AF112939 | Brazil | 1997 |
| 1FS00F97 | AF112940 | Brazil | 1997 |
| 1FT00F97 | AF112941 | Brazil | 1997 |
| 1FU00F97 | AF112943 | Brazil | 1997 |
| 1FV00F97 | AF112944 | Brazil | 1997 |
| 1FW00F97 | AF112945 | Brazil | 1997 |
| 1FY00F97 | AF112947 | Brazil | 1997 |
| 1FZ00F97 | AF112948 | Brazil | 1997 |
| 1GA00F97 | AF112949 | Brazil | 1997 |
| 1GC00F97 | AF112951 | Brazil | 1997 |
| 1GD00F97 | AF112952 | Brazil | 1997 |
| 1GF00F97 | AF112954 | Brazil | 1997 |
| 1GG00F97 | AF112955 | Brazil | 1997 |
| 1GH00F97 | AF112956 | Brazil | 1997 |
| 1GI00F97 | AF112957 | Brazil | 1997 |
| 1GJ00F97 | AF112958 | Brazil | 1997 |
| 1GK00F97 | AF112960 | Brazil | 1997 |
| 1GL00F97 | AF112961 | Brazil | 1997 |
| 1GM00F97 | AF112962 | Brazil | 1997 |
| 1GT00J03 | FJ784163 | Brazil | 2003 |
| 1GV00J03 | FJ784165 | Brazil | 2003 |
| 1GX00J03 | FJ784167 | Brazil | 2003 |
| 1GY00J03 | FJ784168 | Brazil | 2003 |
| 1GZ00J03 | FJ784169 | Brazil | 2003 |
| 1HA00J03 | FJ784170 | Brazil | 2003 |
| 1HB00J03 | FJ784171 | Brazil | 2003 |
| 1HC00J03 | FJ784172 | Brazil | 2003 |
| 1HD00J03 | FJ784173 | Brazil | 2003 |
| 1HF00J03 | FJ784175 | Brazil | 2003 |
| 1HG00J03 | FJ784176 | Brazil | 2003 |
| 1HH00J03 | FJ784177 | Brazil | 2003 |
| 1HI00J03 | FJ784178 | Brazil | 2003 |
| 1HJ00J03 | FJ784179 | Brazil | 2003 |
| 1HL00J03 | FJ784181 | Brazil | 2003 |
| 1HM00J03 | FJ784182 | Brazil | 2003 |
| 1HO00J03 | FJ784184 | Brazil | 2003 |
| 1HQ00J03 | FJ784186 | Brazil | 2003 |
| 1HU00J03 | FJ784190 | Brazil | 2003 |
| 1HV00J03 | FJ784191 | Brazil | 2003 |
| 1HW00J03 | FJ784192 | Brazil | 2003 |
| 1IA00J03 | FJ784196 | Brazil | 2003 |
| 1IC00J03 | FJ784198 | Brazil | 2003 |
| 1IE00J03 | FJ784200 | Brazil | 2003 |
| 1IF00J03 | FJ784201 | Brazil | 2003 |
| 1II00J03 | FJ784204 | Brazil | 2003 |
| 1IJ00J03 | FJ784205 | Brazil | 2003 |
| 1IM00J03 | FJ784208 | Brazil | 2003 |
| 1IP00J03 | FJ784211 | Brazil | 2003 |
| 1IQ00J03 | FJ784212 | Brazil | 2003 |
| 1IR00J03 | FJ784213 | Brazil | 2003 |
| 1IS00J03 | FJ784214 | Brazil | 2003 |
| 1IU00J03 | FJ784216 | Brazil | 2003 |
| 1IX00J03 | FJ784219 | Brazil | 2003 |
| 1IY00J03 | FJ784220 | Brazil | 2003 |
| 1IZ00J03 | FJ784221 | Brazil | 2003 |
| 1JD00J03 | FJ784225 | Brazil | 2003 |
| 1JE00J03 | FJ784226 | Brazil | 2003 |
| 1JF00J03 | FJ784227 | Brazil | 2003 |
| 1JG00J03 | FJ784228 | Brazil | 2003 |
| 1JH00J03 | FJ784229 | Brazil | 2003 |
| 1JI00J03 | FJ784230 | Brazil | 2003 |
| 1JJ00J03 | FJ784231 | Brazil | 2003 |
| 1JN00J03 | FJ784234 | Brazil | 2003 |
| 1JQ00J03 | FJ784237 | Brazil | 2003 |
| 1MF11D03 | EF637046 | Brazil | 2003 |
| 1MG11D03 | EF637047 | Brazil | 2003 |
| 1MH11D03 | EF637048 | Brazil | 2003 |
| 1MJ11D03 | EF637050 | Brazil | 2003 |
| 1MK11D03 | EF637051 | Brazil | 2003 |
| 1MM11D03 | EF637053 | Brazil | 2003 |
| 1MN11D03 | EF637054 | Brazil | 2003 |
| 1MP11D03 | EF637056 | Brazil | 2003 |
| 1MQ10D03 | EF637057 | Brazil | 2003 |
| 1MR00D06 | EU293452 | Brazil | 2006 |
| 1MS00D06 | EU293453 | Brazil | 2006 |
| 1MT00D06 | EU293454 | Brazil | 2006 |
| 1MU00D06 | EU293455 | Brazil | 2006 |
| 1MV00D06 | EU293456 | Brazil | 2006 |
| 1MW00D06 | EU293457 | Brazil | 2006 |
| 1MX00D06 | EU293458 | Brazil | 2006 |
| 1MY00D06 | EU293459 | Brazil | 2006 |
| 1MZ00D06 | EU293460 | Brazil | 2006 |
| 1NA00D06 | EU293461 | Brazil | 2006 |
| 1NB00D06 | EU293462 | Brazil | 2006 |
| 1NC00D06 | EU293463 | Brazil | 2006 |
| 1NE00D06 | EU293465 | Brazil | 2006 |
| 1NF00D06 | EU293466 | Brazil | 2006 |
| 1NG00D06 | EU293467 | Brazil | 2006 |
| 1NH00D06 | EU293468 | Brazil | 2006 |
| 1NI00D06 | EU293469 | Brazil | 2006 |
| 1NJ00D06 | EU293470 | Brazil | 2006 |
| 1NK00D06 | EU293471 | Brazil | 2006 |
| 1NL00D06 | EU293472 | Brazil | 2006 |
| 1NM00D06 | EU293473 | Brazil | 2006 |
| 1NN00D06 | EU293474 | Brazil | 2006 |
| 1NO00D06 | EU293475 | Brazil | 2006 |
| 1NP00D06 | EU293476 | Brazil | 2006 |
| 1NQ00D06 | EU293477 | Brazil | 2006 |
| 1NR00D06 | EU293478 | Brazil | 2006 |
| 1NS00D06 | EU293479 | Brazil | 2006 |
| 1NT00D06 | EU293480 | Brazil | 2006 |
| 1NU00D06 | EU293481 | Brazil | 2006 |
| 1NV00D06 | EU293482 | Brazil | 2006 |
| 1NW00D06 | EU293483 | Brazil | 2006 |
| 1NX00D06 | EU293484 | Brazil | 2006 |
| 1NY00D06 | EU293485 | Brazil | 2006 |
| 1OB00D06 | EU293488 | Brazil | 2006 |
| 1OC00D06 | EU293489 | Brazil | 2006 |
| 1OD00D06 | EU293490 | Brazil | 2006 |
| 1OF00D06 | EU293492 | Brazil | 2006 |
| 1OG00D06 | EU293493 | Brazil | 2006 |
| 1OH00D06 | EU293494 | Brazil | 2006 |
| 1OI00D06 | EU293495 | Brazil | 2006 |
| 1OJ00D06 | EU293496 | Brazil | 2006 |
| 1OK00D06 | EU293497 | Brazil | 2006 |
| 1OL00D06 | EU293498 | Brazil | 2006 |
| 1ON00D06 | EU293500 | Brazil | 2006 |
| 1OO00D06 | EU293501 | Brazil | 2006 |
| 1OP00D06 | EU293502 | Brazil | 2006 |
| 1OQ00D06 | EU293503 | Brazil | 2006 |
| 1OR00D06 | EU293504 | Brazil | 2006 |
| 1QC00A05 | EF379160 | Brazil | 2005 |
| 1QD00A05 | EF379161 | Brazil | 2005 |
| 1QL00A05 | EF379169 | Brazil | 2005 |
| 1QP00B05 | EF379173 | Brazil | 2005 |
| 1QY00A06 | EF379182 | Brazil | 2006 |
| 1QZ00A06 | EF379183 | Brazil | 2006 |
| 1RD00A06 | EF379187 | Brazil | 2006 |
| 1RE00A06 | EF379188 | Brazil | 2006 |
| 1RK00A06 | EF379194 | Brazil | 2006 |
| 1RQ00B06 | EF379200 | Brazil | 2006 |
| 1RR00B06 | EF379201 | Brazil | 2006 |
| 1RS00B06 | EF379202 | Brazil | 2006 |
| 1SA00B06 | EF379210 | Brazil | 2006 |
| 1SF22B08 | GQ401297 | Brazil | 2008 |
| 1SS22B07 | GQ401310 | Brazil | 2007 |
| 1TP00JN | FJ618909 | Brazil | N |
| 1TQ00JN | FJ618910 | Brazil | N |
| 1TR00JN | FJ638422 | Brazil | N |
| 1TT22JN | FJ638424 | Brazil | N |
| 1TU00JN | FJ638425 | Brazil | N |
| 1TV11JN | FJ638426 | Brazil | N |
| 1TW00JN | FJ638427 | Brazil | N |
| 1TZ15JN | FJ638430 | Brazil | N |
| 1UA22JN | FJ638431 | Brazil | N |
| 1UC00JN | FJ638433 | Brazil | N |
| 1UD00JN | FJ638434 | Brazil | N |
| 1UE00JN | FJ638435 | Brazil | N |
| 1UF11JN | FJ638436 | Brazil | N |
| 1UI00JN | FJ638439 | Brazil | N |
| 1UJ00JN | FJ638440 | Brazil | N |
| 1UL00JN | FJ638442 | Brazil | N |
| 1UP00JN | FJ638446 | Brazil | N |
| 1UQ00JN | FJ638447 | Brazil | N |
| 1UR12JN | FJ638448 | Brazil | N |
| 1UV10JN | FJ638452 | Brazil | N |
| 1UW00JN | FJ638453 | Brazil | N |
| 1UY00JN | FJ638455 | Brazil | N |
| 1UZ11JN | FJ638456 | Brazil | N |
| 1WA25JN | FJ638457 | Brazil | N |
| 1WE00JN | FJ638461 | Brazil | N |
| 1WK00JN | FJ638467 | Brazil | N |
| 1WL05G07 | GQ862343 | Brazil | 2007 |
| 1WN05G07 | GQ862319 | Brazil | 2007 |
| 1WO05G03 | GQ862335 | Brazil | 2003 |
| 1WP05G07 | GQ862328 | Brazil | 2007 |
| 1WQ05G05 | GQ862310 | Brazil | 2005 |
| 1WT05G04 | GQ862336 | Brazil | 2004 |
| 1WU05G07 | GQ862305 | Brazil | 2007 |
| 1WV05G02 | GQ862333 | Brazil | 2002 |
| 1WW05G07 | GQ862320 | Brazil | 2007 |
| 1WX05G07 | GQ862317 | Brazil | 2007 |
| 1WY05G07 | GQ862321 | Brazil | 2007 |
| 1WZ05G04 | GQ862339 | Brazil | 2004 |
| 1XA05G06 | GQ862332 | Brazil | 2006 |
| 1XB05G07 | GQ862324 | Brazil | 2007 |
| 1XC05G07 | GQ862322 | Brazil | 2007 |
| 1XD05G07 | GQ862330 | Brazil | 2007 |
| 1XE05G04 | GQ862338 | Brazil | 2004 |
| 1XF05G06 | GQ862331 | Brazil | 2006 |
| 1XJ05G04 | GQ862342 | Brazil | 2004 |
| 1XL05G07 | GQ862315 | Brazil | 2007 |
| 1XM05G05 | GQ862337 | Brazil | 2005 |
| 1XQ05G07 | GQ862316 | Brazil | 2007 |
| 1XT05G07 | GQ862325 | Brazil | 2007 |
| 1XV05G07 | GQ862329 | Brazil | 2007 |
| 1XX05G06 | GQ862312 | Brazil | 2006 |
| 1YE10H08 | JF342278 | Brazil | 2008 |
| 1YM10H08 | JF342286 | Brazil | 2008 |
| 1ZK10H08 | JF342310 | Brazil | 2008 |
| 1ZO10H10 | JF342314 | Brazil | 2010 |
| 1ZX00MN | GU214067 | Brazil | N |
| 1ZZ00MN | GU214069 | Brazil | N |
| 10A00MN | GU214070 | Brazil | N |
| 10C00MN | GU214072 | Brazil | N |
| 10D00MN | GU214073 | Brazil | N |
| 10E00MN | GU214074 | Brazil | N |
| 10H00MN | GU214077 | Brazil | N |
| 10I00MN | GU214078 | Brazil | N |
| 10J00MN | GU214079 | Brazil | N |
| 10L00MN | GU214081 | Brazil | N |
| 10M00MN | GU214082 | Brazil | N |
| 10N22MN | GU214083 | Brazil | N |
| 10O00MN | GU214084 | Brazil | N |
| 10R00MN | GU214087 | Brazil | N |
| 10T00MN | GU214089 | Brazil | N |
| 10U00MN | GU214090 | Brazil | N |
| 10V00MN | GU214091 | Brazil | N |
| 10W00MN | GU214092 | Brazil | N |
| 10Y11MN | GU214094 | Brazil | N |
| 10Z00MN | GU214095 | Brazil | N |
| 11A00MN | GU214096 | Brazil | N |
| 11D00MN | GU214099 | Brazil | N |
| 11E22MN | GU214100 | Brazil | N |
| 11F00MN | GU214101 | Brazil | N |
| 11H00MN | GU214103 | Brazil | N |
| 11I00MN | GU214104 | Brazil | N |
| 11J00MN | GU214105 | Brazil | N |
| 11K00MN | GU214106 | Brazil | N |
| 11L00MN | GU214107 | Brazil | N |
| 11M00MN | GU214108 | Brazil | N |
| 11N00MN | GU214109 | Brazil | N |
| 11O00MN | GU214110 | Brazil | N |
| 11Q22MN | HM583573 | Brazil | N |
| 11R00MN | HM583574 | Brazil | N |
| 11S00MN | HM583575 | Brazil | N |
| 11T00MN | HM583576 | Brazil | N |
| 11U00MN | HM583577 | Brazil | N |
| 11W00MN | HM583579 | Brazil | N |
| 11Z00MN | HM583582 | Brazil | N |
| 12A00MN | HM583583 | Brazil | N |
| 12B11MN | HM583584 | Brazil | N |
| 12C00MN | HM583585 | Brazil | N |
| 12D00MN | HM583586 | Brazil | N |
| 12F20H08 | HQ831451 | Brazil | 2008 |
| 12I10H07 | HQ831454 | Brazil | 2007 |
| 12J10H07 | HQ831455 | Brazil | 2007 |
| 12K10H07 | HQ831456 | Brazil | 2007 |
| 12L10H07 | HQ831457 | Brazil | 2007 |
| 12M10H07 | HQ831458 | Brazil | 2007 |
| 12Q10H07 | HQ831462 | Brazil | 2007 |
| 13A10J10 | HQ831472 | Brazil | 2010 |
| 13B10J10 | HQ831473 | Brazil | 2010 |
| 13C10J10 | HQ831474 | Brazil | 2010 |
| 13E10J10 | HQ831476 | Brazil | 2010 |
| 13F00DN | HM024737 | Brazil | N |
| 13H00D05 | HM024739 | Brazil | 2005 |
| 13I00D05 | HM024740 | Brazil | 2005 |
| 13J00D05 | HM024741 | Brazil | 2005 |
| 15R11R09 | HQ127459 | Brazil | 2009 |
| 15S11E09 | HQ127460 | Brazil | 2009 |
| 15T11E09 | HQ127461 | Brazil | 2009 |
| 15V11E09 | HQ127463 | Brazil | 2009 |
| 15X11E09 | HQ127465 | Brazil | 2009 |
| 15Y11E09 | HQ127466 | Brazil | 2009 |
| 15Z11E09 | HQ127467 | Brazil | 2009 |
| 16A11E09 | HQ127468 | Brazil | 2009 |
| 16B11E09 | HQ127469 | Brazil | 2009 |
| 16D11E09 | HQ127471 | Brazil | 2009 |
| 16E11E09 | HQ127472 | Brazil | 2009 |
| 16F11E09 | HQ127473 | Brazil | 2009 |
| 16H11E09 | HQ127475 | Brazil | 2009 |
| 16J11E09 | HQ127477 | Brazil | 2009 |
| 16K11E09 | HQ127478 | Brazil | 2009 |
| 16N11E09 | HQ127481 | Brazil | 2009 |
| 16P11E09 | HQ127483 | Brazil | 2009 |
| 16R11E09 | HQ127485 | Brazil | 2009 |
| 16U11E09 | HQ127488 | Brazil | 2009 |
| 16V11E09 | HQ127489 | Brazil | 2009 |
| 16W11E09 | HQ127490 | Brazil | 2009 |
| 16X11E09 | HQ127491 | Brazil | 2009 |
| 16Y11E09 | HQ127492 | Brazil | 2009 |
| 17A11E09 | HQ127494 | Brazil | 2009 |
| 17B11E09 | HQ127495 | Brazil | 2009 |
| 17C11E09 | HQ127496 | Brazil | 2009 |
| 17D11E09 | HQ127497 | Brazil | 2009 |
| 17E11E09 | HQ127498 | Brazil | 2009 |
| 17G11E09 | HQ127500 | Brazil | 2009 |
| 17J11G09 | HQ127503 | Brazil | 2009 |
| 17N11E09 | HQ127507 | Brazil | 2009 |
| 17O11E09 | HQ127508 | Brazil | 2009 |
| 17Q11E09 | HQ127510 | Brazil | 2009 |
| 17R11E09 | HQ127511 | Brazil | 2009 |
| 17S11E09 | HQ127512 | Brazil | 2009 |
| 17T11E09 | HQ127513 | Brazil | 2009 |
| 17W11E09 | HQ127516 | Brazil | 2009 |
| 17X11B09 | HQ127517 | Brazil | 2009 |
| 17Z11H09 | HQ127519 | Brazil | 2009 |
| 18A11H09 | HQ127520 | Brazil | 2009 |
| 18E11O09 | HQ127524 | Brazil | 2009 |
| 18F11H09 | HQ127525 | Brazil | 2009 |
| 18H11H09 | HQ127527 | Brazil | 2009 |
| 18I11B09 | HQ127528 | Brazil | 2009 |
| 18K11H09 | HQ127530 | Brazil | 2009 |
| 18L11O09 | HQ127531 | Brazil | 2009 |
| 18M11O09 | HQ127532 | Brazil | 2009 |
| 18N11O09 | HQ127533 | Brazil | 2009 |
| 18O11O09 | HQ127534 | Brazil | 2009 |
| 18Q11O09 | HQ127536 | Brazil | 2009 |
| 18S11O09 | HQ127538 | Brazil | 2009 |
| 18T11O09 | HQ127539 | Brazil | 2009 |
| 18U11O09 | HQ127540 | Brazil | 2009 |
| 18V11O09 | HQ127541 | Brazil | 2009 |
| 18W11O09 | HQ127542 | Brazil | 2009 |
| 18X11O09 | HQ127543 | Brazil | 2009 |
| 18Y11E09 | HQ127544 | Brazil | 2009 |
| 18Z11H09 | HQ127545 | Brazil | 2009 |
| 19A11H09 | HQ127546 | Brazil | 2009 |
| 19C11L09 | HQ127548 | Brazil | 2009 |
| 19D11O09 | HQ127549 | Brazil | 2009 |
| 19F11L09 | HQ127551 | Brazil | 2009 |
| 19H11L09 | HQ127553 | Brazil | 2009 |
| 19I11L09 | HQ127554 | Brazil | 2009 |
| 19J11L09 | HQ127555 | Brazil | 2009 |
| 19K11L09 | HQ127556 | Brazil | 2009 |
| 19M11O09 | HQ127558 | Brazil | 2009 |
| 19N11G09 | HQ127559 | Brazil | 2009 |
| 19P11L09 | HQ127561 | Brazil | 2009 |
| 19R11L09 | HQ127563 | Brazil | 2009 |
| 19S11D09 | HQ127564 | Brazil | 2009 |
| 19U11O09 | HQ127566 | Brazil | 2009 |
| 19V11L09 | HQ127567 | Brazil | 2009 |
| 19W11O09 | HQ127568 | Brazil | 2009 |
| 19Y11C09 | HQ127570 | Brazil | 2009 |
| 19Z11L09 | HQ127571 | Brazil | 2009 |
| 2AA11N09 | HQ127572 | Brazil | 2009 |
| 2AB11N09 | HQ127573 | Brazil | 2009 |
| 2AC11N09 | HQ127574 | Brazil | 2009 |
| 2AD11N09 | HQ127575 | Brazil | 2009 |
| 2AE11C09 | HQ127576 | Brazil | 2009 |
| 2AF11N09 | HQ127577 | Brazil | 2009 |
| 2AG11C09 | HQ127578 | Brazil | 2009 |
| 2AI11O09 | HQ127580 | Brazil | 2009 |
| 2AJ11L09 | HQ127581 | Brazil | 2009 |
| 2AL11E09 | HQ127583 | Brazil | 2009 |
| 2AN11O09 | HQ127585 | Brazil | 2009 |
| 2AP11L09 | HQ127587 | Brazil | 2009 |
| 2AQ11N09 | HQ127588 | Brazil | 2009 |
| 2AS11R09 | HQ127590 | Brazil | 2009 |
| 2AT11N09 | HQ127591 | Brazil | 2009 |
| 2AV11N09 | HQ127593 | Brazil | 2009 |
| 2AW11C09 | HQ127594 | Brazil | 2009 |
| 2AX11C09 | HQ127595 | Brazil | 2009 |
| 2AZ11C09 | HQ127597 | Brazil | 2009 |
| 2BB11E09 | HQ127599 | Brazil | 2009 |
| 2BD11O09 | HQ127601 | Brazil | 2009 |
| 2BE11L09 | HQ127602 | Brazil | 2009 |
| 2BG11O09 | HQ127604 | Brazil | 2009 |
| 2BI11O09 | HQ127606 | Brazil | 2009 |
| 2BJ11O09 | HQ127607 | Brazil | 2009 |
| 2BK11O09 | HQ127608 | Brazil | 2009 |
| 2BL11E09 | HQ127609 | Brazil | 2009 |
| 2BM11H09 | HQ127610 | Brazil | 2009 |
| 2BN11R09 | HQ127611 | Brazil | 2009 |
| 2BO11R09 | HQ127612 | Brazil | 2009 |
| 2BP11R09 | HQ127613 | Brazil | 2009 |
| 2BQ11R09 | HQ127614 | Brazil | 2009 |
| 2BR11R09 | HQ127615 | Brazil | 2009 |
| 2BT11R09 | HQ127617 | Brazil | 2009 |
| 2BV11R09 | HQ127619 | Brazil | 2009 |
| 2BW11R09 | HQ127620 | Brazil | 2009 |
| 2CB00AN | AY275721 | Brazil | N |
| 2CC00AN | AY275722 | Brazil | N |
| 2CD00AN | AY275723 | Brazil | N |
| 2CF00AN | AY275725 | Brazil | N |
| 2CG00AN | AY275726 | Brazil | N |
| 2CH00AN | AY275727 | Brazil | N |
| 2CL00AN | AY275731 | Brazil | N |
| 2CS00AN | AY275738 | Brazil | N |
| 2CV00AN | AY275741 | Brazil | N |
| 2CW00AN | AY275742 | Brazil | N |
| 2CX00AN | AY275743 | Brazil | N |
| 2CY00AN | AY275744 | Brazil | N |
| 2CZ00AN | AY275745 | Brazil | N |
| 2DF00AN | AY275751 | Brazil | N |
| 2DN00AN | AY275759 | Brazil | N |
| 2DO00AN | AY275760 | Brazil | N |
| 2DP00AN | AY275761 | Brazil | N |
| 2DR00AN | AY275763 | Brazil | N |
| 2DS00AN | AY275764 | Brazil | N |
| 2DU00AN | AY275766 | Brazil | N |
| 2DV00AN | AY275767 | Brazil | N |
| 2EE00AN | DQ190964 | Brazil | N |
| 2EF00AN | DQ190965 | Brazil | N |
| 2EG00AN | DQ190966 | Brazil | N |
| 2EH00AN | DQ190967 | Brazil | N |
| 2EI00AN | DQ190968 | Brazil | N |
| 2EN00AN | DQ343987 | Brazil | N |
| 2EO00AN | DQ343988 | Brazil | N |
| 2EP00AN | DQ343989 | Brazil | N |
| 2ER00AN | DQ343992 | Brazil | N |
| 2ES00AN | DQ343993 | Brazil | N |
| 2ET00AN | DQ343994 | Brazil | N |
| 2EU00AN | DQ343995 | Brazil | N |
| 2EV00AN | DQ343996 | Brazil | N |
| 2FE00AN | DQ344007 | Brazil | N |
| 2FF00AN | DQ344008 | Brazil | N |
| 2FG00AN | DQ344009 | Brazil | N |
| 2FK00A06 | DQ344013 | Brazil | 2006 |
| 2FL00A06 | DQ344015 | Brazil | 2006 |
| 2FM00A06 | DQ344016 | Brazil | 2006 |
| 2FP20J08 | JN114115 | Brazil | 2008 |
| 2FR20J08 | JN114118 | Brazil | 2008 |
| 2FS20J08 | JN114120 | Brazil | 2008 |
| 2FU20J08 | JN114126 | Brazil | 2008 |
| 2FV20J08 | JN114127 | Brazil | 2008 |
| 2GB20J08 | JN114135 | Brazil | 2008 |
| 2GC20J08 | JN114136 | Brazil | 2008 |
| 2GE20J08 | JN114138 | Brazil | 2008 |
| 2GF20J09 | JN114140 | Brazil | 2009 |
| 2GG20J09 | JN114141 | Brazil | 2009 |
| 2GI20J09 | JN114143 | Brazil | 2009 |
| 2GK20J09 | JN114147 | Brazil | 2009 |
| 2GL20J09 | JN114149 | Brazil | 2009 |
| 2GQ20J09 | JN114155 | Brazil | 2009 |
| 2GR20J09 | JN114159 | Brazil | 2009 |
| 2GT20J09 | JN114164 | Brazil | 2009 |
| 2GU20J09 | JN114168 | Brazil | 2009 |
| 2GV20J09 | JN114169 | Brazil | 2009 |
| 2GW20J09 | JN114170 | Brazil | 2009 |
| 2GX20J09 | JN114171 | Brazil | 2009 |
| 2GY20J09 | JN114173 | Brazil | 2009 |
| 2HA20J09 | JN114175 | Brazil | 2009 |
| 2HD20JN | JN114180 | Brazil | N |
| 2HF20J09 | JN114183 | Brazil | 2009 |
| 2HH20J09 | JN114185 | Brazil | 2009 |
| 2HI20J09 | JN114186 | Brazil | 2009 |
| 2HK20J09 | JN114188 | Brazil | 2009 |
| 2HQ20J09 | JN114195 | Brazil | 2009 |
| 2HR20J09 | JN114199 | Brazil | 2009 |
| 2HS20J09 | JN114201 | Brazil | 2009 |
| 2HT20J09 | JN114202 | Brazil | 2009 |
| 2HU20J09 | JN114203 | Brazil | 2009 |
| 2HV20J09 | JN114206 | Brazil | 2009 |
| 2HW20J09 | JN114207 | Brazil | 2009 |
| 2HZ20J10 | JN114217 | Brazil | 2010 |
| 2IA20J10 | JN114222 | Brazil | 2010 |
| 2IB20J10 | JN114225 | Brazil | 2010 |
| 2IE20J09 | JN114235 | Brazil | 2009 |
| 2IG20J09 | JN662427 | Brazil | 2009 |
| 2JC00JN | FJ548787 | Brazil | N |
| 2JD00JN | FJ548788 | Brazil | N |
| 2JF00JN | FJ548790 | Brazil | N |
| 2JI00JN | FJ548793 | Brazil | N |
| 2JP00JN | FJ548800 | Brazil | N |
| 2JQ00JN | FJ548801 | Brazil | N |
| 2JR00JN | FJ548802 | Brazil | N |
| 2JS00JN | FJ548803 | Brazil | N |
| 2JT00JN | FJ548804 | Brazil | N |
| 2JX00JN | FJ548808 | Brazil | N |
| 2JY00JN | FJ548809 | Brazil | N |
| 2JZ00JN | FJ548810 | Brazil | N |
| 2KA00JN | FJ548811 | Brazil | N |
| 2KB11JN | FJ548812 | Brazil | N |
| 2KC11JN | FJ548813 | Brazil | N |
| 2KD00JN | FJ548814 | Brazil | N |
| 2KE00JN | FJ548815 | Brazil | N |
| 2KF00JN | FJ548816 | Brazil | N |
| 2KG00JN | FJ548817 | Brazil | N |
| 2KJ00JN | FJ548820 | Brazil | N |
| 2KL12JN | FJ548822 | Brazil | N |
| 2KM00JN | FJ548823 | Brazil | N |
| 2KN00JN | FJ594127 | Brazil | N |
| 2KO00JN | FJ594128 | Brazil | N |
| 2KP00JN | FJ594129 | Brazil | N |
| 2KQ00JN | FJ594130 | Brazil | N |
| 2KR00JN | FJ594131 | Brazil | N |
| 2KS00JN | FJ594132 | Brazil | N |
| 2KT00JN | FJ594133 | Brazil | N |
| 2KU00JN | FJ594134 | Brazil | N |
| 2KX00JN | FJ594137 | Brazil | N |
| 2KY00JN | FJ594138 | Brazil | N |
| 2KZ00JN | FJ594139 | Brazil | N |
| 2LA14JN | FJ594140 | Brazil | N |
| 2LB00JN | FJ594141 | Brazil | N |
| 2LC00JN | FJ594142 | Brazil | N |
| 2LD00JN | FJ594143 | Brazil | N |
| 2LF00JN | FJ594145 | Brazil | N |
| 2LH00JN | FJ594147 | Brazil | N |
| 2LI00JN | FJ594148 | Brazil | N |
| 2LK00JN | FJ594150 | Brazil | N |
| 2LM00JN | FJ594152 | Brazil | N |
| 2LO00JN | FJ594154 | Brazil | N |
| 2LQ00JN | FJ594156 | Brazil | N |
| 2LS00JN | FJ594158 | Brazil | N |
| 2LT22JN | FJ594159 | Brazil | N |
| 2LU11JN | FJ594160 | Brazil | N |
| 2LV00JN | FJ594161 | Brazil | N |
| 2LW00JN | FJ594162 | Brazil | N |
| 2LY00JN | FJ594164 | Brazil | N |
| 2LZ00JN | FJ594165 | Brazil | N |
| 2MA00JN | FJ594166 | Brazil | N |
| 2MB00JN | FJ594167 | Brazil | N |
| 2MC00JN | FJ594168 | Brazil | N |
| 2MF00JN | FJ594171 | Brazil | N |
| 2MH00JN | FJ594173 | Brazil | N |
| 2MJ22JN | FJ594175 | Brazil | N |
| 2ML00JN | FJ594177 | Brazil | N |
| 2MP00JN | FJ594181 | Brazil | N |
| 2MQ11JN | FJ594182 | Brazil | N |
| 2MR11JN | FJ594183 | Brazil | N |
| 2MS00JN | FJ594184 | Brazil | N |
| 2MU20E01 | AY929012 | Brazil | 2001 |
| 2MV12E01 | AY929013 | Brazil | 2001 |
| 2MW12E01 | AY929014 | Brazil | 2001 |
| 2MY10E01 | AY929016 | Brazil | 2001 |
| 2MZ22E01 | AY929017 | Brazil | 2001 |
| 2NA22E01 | AY929018 | Brazil | 2001 |
| 2NB20E01 | AY929019 | Brazil | 2001 |
| 2NC10E02 | AY929020 | Brazil | 2002 |
| 2ND20E02 | AY929021 | Brazil | 2002 |
| 2NE20E01 | AY929022 | Brazil | 2001 |
| 2NF20E01 | AY929023 | Brazil | 2001 |
| 2NG20E01 | AY929024 | Brazil | 2001 |
| 2NH22E02 | AY929025 | Brazil | 2002 |
| 2NI22E02 | AY929026 | Brazil | 2002 |
| 2NJ14E02 | AY929027 | Brazil | 2002 |
| 2NK22E02 | AY929028 | Brazil | 2002 |
| 2NL10E02 | AY929029 | Brazil | 2002 |
| 2NM12E02 | AY929030 | Brazil | 2002 |
| 2NN10E02 | AY929031 | Brazil | 2002 |
| 2NO22E02 | AY929032 | Brazil | 2002 |
| 2NP10E02 | AY929033 | Brazil | 2002 |
| 2NS20E03 | AY929036 | Brazil | 2003 |
| 2NT22E01 | AY929037 | Brazil | 2001 |
| 2NW12E01 | AY929040 | Brazil | 2001 |
| 2OA10E02 | AY929044 | Brazil | 2002 |
| 2OG20E04 | AY929050 | Brazil | 2004 |
| 2OJ20E04 | AY929053 | Brazil | 2004 |
| 2OK10E04 | AY929054 | Brazil | 2004 |
| 2OL22E04 | AY929055 | Brazil | 2004 |
| 2ON20E04 | AY929057 | Brazil | 2004 |
| 2OO20E04 | AY929058 | Brazil | 2004 |
| 2OP22E03 | AY929059 | Brazil | 2003 |
| 2OQ20E04 | AY929060 | Brazil | 2004 |
| 2OR10E04 | AY929061 | Brazil | 2004 |
| 2OS11E05 | DQ058780 | Brazil | 2005 |
| 2OT20E05 | DQ058781 | Brazil | 2005 |
| 2OU11E05 | DQ058782 | Brazil | 2005 |
| 2OV10E05 | DQ058783 | Brazil | 2005 |
| 2OW20EN | DQ224357 | Brazil | N |
| 2OX10EN | DQ224354 | Brazil | N |
| 2OY20EN | DQ224355 | Brazil | N |
| 2PH22D02 | DQ358805 | Brazil | 2002 |
| 2PI22D02 | DQ358806 | Brazil | 2002 |
| 2PJ22D02 | DQ358807 | Brazil | 2002 |
| 2PK11D02 | DQ358808 | Brazil | 2002 |
| 2PL13D02 | DQ358809 | Brazil | 2002 |
| 2PM22D02 | DQ358810 | Brazil | 2002 |
| 2PQ122N | AF470810 | Argentina | N |
| 2PT11202 | DQ383746 | Argentina | 2002 |
| 2PU11202 | DQ383748 | Argentina | 2002 |
| 2PV11202 | DQ383749 | Argentina | 2002 |
| 2PX11202 | DQ383752 | Argentina | 2002 |
| 2PZ15298 | AY037268 | Argentina | 1998 |
| 2QH22202 | EF190996 | Argentina | 2002 |
| 2QI22202 | EF190997 | Argentina | 2002 |
| 2QL12299 | AY037282 | Argentina | 1999 |
| 2QN22200 | AY037269 | Argentina | 2000 |
| 2QP22499 | AY037270 | Bolivia | 1999 |
| 2QQ002N | DQ206664 | Argentina | N |
| 2QR002N | DQ206665 | Argentina | N |
| 2QS002N | DQ206666 | Argentina | N |
| 2QT002N | DQ206667 | Argentina | N |
| 2QU002N | DQ206668 | Argentina | N |
| 2QV002N | DQ206669 | Argentina | N |
| 2QW002N | DQ206670 | Argentina | N |
| 2QX002N | DQ206671 | Argentina | N |
| 2QY002N | DQ206672 | Argentina | N |
| 2QZ002N | DQ206673 | Argentina | N |
| 2RA002N | DQ206674 | Argentina | N |
| 2RB002N | DQ345506 | Argentina | N |
| 2RC002N | DQ345507 | Argentina | N |
| 2RD002N | DQ345508 | Argentina | N |
| 2RE002N | DQ345509 | Argentina | N |
| 2RF002N | DQ345510 | Argentina | N |
| 2RG002N | DQ345511 | Argentina | N |
| 2RH002N | DQ345512 | Argentina | N |
| 2RJ002N | DQ345514 | Argentina | N |
| 2RK002N | DQ345515 | Argentina | N |
| 2SY005N | EF193884 | Chile | N |
| 2TB005N | EF193887 | Chile | N |
| 2TC005N | EF193888 | Chile | N |
| 2TE005N | EF193890 | Chile | N |
| 2TH005N | EF193893 | Chile | N |
| 2TI005N | EF193894 | Chile | N |
| 2TJ005N | EF193895 | Chile | N |
| 2TL005N | EF193897 | Chile | N |
| 2TO005N | EF193900 | Chile | N |
| 2TP005N | EF193901 | Chile | N |
| 2TQ005N | EF193902 | Chile | N |
| 2TR005N | EF193903 | Chile | N |
| 2TS005N | EF193904 | Chile | N |
| 2TT005N | EF193905 | Chile | N |
| 2TU005N | EF193906 | Chile | N |
| 2TV005N | EF193907 | Chile | N |
| 2TW005N | EF193908 | Chile | N |
| 2TX005N | EF193909 | Chile | N |
| 2TY005N | EF193910 | Chile | N |
| 2TZ005N | EF193911 | Chile | N |
| 2UB005N | EF193913 | Chile | N |
| 2UC005N | EF193914 | Chile | N |
| 2UD005N | EF193915 | Chile | N |
| 2UE005N | EF193916 | Chile | N |
| 2UF005N | EF193917 | Chile | N |
| 2UG005N | EF193918 | Chile | N |
| 2UH005N | EF193919 | Chile | N |
| 2UI005N | EF193920 | Chile | N |
| 2UJ005N | EF193921 | Chile | N |
| 2UK005N | EF193922 | Chile | N |
| 2UL005N | EF193923 | Chile | N |
| 2UM005N | EF193924 | Chile | N |
| 2UN005N | EF193925 | Chile | N |
| 2UQ005N | EF193928 | Chile | N |
| 2UR005N | EF193929 | Chile | N |
| 2US005N | EF193930 | Chile | N |
| 2UT005N | EF193931 | Chile | N |
| 2UU005N | EF193932 | Chile | N |
| 2UV005N | EF193933 | Chile | N |
| 2UW005N | EF193934 | Chile | N |
| 2UX005N | EF193935 | Chile | N |
| 2UY005N | EF193936 | Chile | N |
| 2UZ005N | EF193937 | Chile | N |
| 2WA005N | EF193938 | Chile | N |
| 2WB005N | EF193939 | Chile | N |
| 2WC005N | EF193940 | Chile | N |
| 2WD005N | EF193941 | Chile | N |
| 2WE005N | EF193942 | Chile | N |
| 2WF005N | EF193943 | Chile | N |
| 2WG005N | EF193944 | Chile | N |
| 2WI005N | EF193946 | Chile | N |
| 2WJ005N | EF193947 | Chile | N |
| 2WK005N | EF193948 | Chile | N |
| 2WL005N | EF193949 | Chile | N |
| 2WM005N | EF193950 | Chile | N |
| 2WN005N | EF193951 | Chile | N |
| 2WO005N | EF193952 | Chile | N |
| 2WP005N | EF193953 | Chile | N |
| 2WR005N | EF193955 | Chile | N |
| 2WS005N | EF193956 | Chile | N |
| 2WT005N | EF193957 | Chile | N |
| 2WU005N | EF193958 | Chile | N |
| 2WV005N | EF193959 | Chile | N |
| 2WW005N | EF193960 | Chile | N |
| 2WX005N | EF193961 | Chile | N |
| 2XA005N | EF193964 | Chile | N |
| 2XB005N | EF193965 | Chile | N |
| 2XC005N | EF193966 | Chile | N |
| 2XD005N | EF193967 | Chile | N |
| 2XE005N | EF193968 | Chile | N |
| 2XF005N | EF193969 | Chile | N |
| 2XG005N | EF193970 | Chile | N |
| 2XH005N | EF193971 | Chile | N |
| 2XI005N | EF193972 | Chile | N |
| 2XJ005N | EF193973 | Chile | N |
| 2XK005N | EF193974 | Chile | N |
| 2XL005N | EF193975 | Chile | N |
| 2XM005N | EF193976 | Chile | N |
| 2XN005N | EF193977 | Chile | N |
| 2XO005N | EF193978 | Chile | N |
| 2XQ005N | EF193980 | Chile | N |
| 2XR005N | EF193981 | Chile | N |
| 2XS005N | EF193982 | Chile | N |
| 2XT005N | EF193983 | Chile | N |
| 2XU005N | EF193984 | Chile | N |
| 2XV005N | EF193985 | Chile | N |
| 2XX005N | EF193987 | Chile | N |
| 2XY005N | EF193988 | Chile | N |
| 2XZ005N | EF193989 | Chile | N |
| 2YA005N | EF193990 | Chile | N |
| 2YB005N | EF193991 | Chile | N |
| 2YC005N | EF193992 | Chile | N |
| 2YD005N | EF193993 | Chile | N |
| 2YF005N | EF193995 | Chile | N |
| 2YG005N | EF193996 | Chile | N |
| 2YH005N | EF193997 | Chile | N |
| 2YI005N | EF193998 | Chile | N |
| 2YJ005N | EF193999 | Chile | N |
| 2YK005N | EF194000 | Chile | N |
| 2YL005N | EF194001 | Chile | N |
| 2YM005N | EF194002 | Chile | N |
| 2YN005N | EF194003 | Chile | N |
| 2YO005N | EF194004 | Chile | N |
| 2YP005N | EF194005 | Chile | N |
| 2YQ005N | EF194006 | Chile | N |
| 2YS005N | EF194008 | Chile | N |
| 2YT005N | EF194009 | Chile | N |
| 2YU005N | EF194010 | Chile | N |
| 2YX005N | EF194013 | Chile | N |
| 2YY005N | EF194014 | Chile | N |
| 2YZ005N | EF194015 | Chile | N |
| 2ZA005N | EF194016 | Chile | N |
| 2ZC005N | EF194018 | Chile | N |
| 2ZD005N | EF194019 | Chile | N |
| 2ZE005N | EF194020 | Chile | N |
| 2ZF005N | EF194021 | Chile | N |
| 2ZH005N | EF194023 | Chile | N |
| 2ZJ005N | EF194025 | Chile | N |
| 2ZL005N | EF194027 | Chile | N |
| 2ZM005N | EF194028 | Chile | N |
| 2ZN005N | EF194029 | Chile | N |
| 2ZO005N | EF194030 | Chile | N |
| 2ZP005N | EF194031 | Chile | N |
| 2ZQ005N | EF194032 | Chile | N |
| 2ZR005N | EF194033 | Chile | N |
| 2ZS005N | EF194034 | Chile | N |
| 2ZT005N | EF194035 | Chile | N |
| 2ZU005N | EF194036 | Chile | N |
| 2ZW005N | EF194038 | Chile | N |
| 2ZX005N | EF194039 | Chile | N |
| 2ZY11600 | AY566148 | Colombia | 2000 |
| 2ZZ11601 | AY566149 | Colombia | 2001 |
| 20A11600 | AY566150 | Colombia | 2000 |
| 20B11601 | AY566151 | Colombia | 2001 |
| 20C11602 | AY566152 | Colombia | 2002 |
| 20D00602 | AY566153 | Colombia | 2002 |
| 20F20602 | AY566155 | Colombia | 2002 |
| 20I12601 | AY566125 | Colombia | 2001 |
| 20J11601 | AY566126 | Colombia | 2001 |
| 20K11601 | AY566127 | Colombia | 2001 |
| 20L13601 | AY566128 | Colombia | 2001 |
| 20N12601 | AY566130 | Colombia | 2001 |
| 20O12601 | AY566131 | Colombia | 2001 |
| 20P11600 | AY566132 | Colombia | 2000 |
| 20Q11601 | AY566133 | Colombia | 2001 |
| 20R12601 | AY566134 | Colombia | 2001 |
| 20S12601 | AY566135 | Colombia | 2001 |
| 20T12601 | AY566136 | Colombia | 2001 |
| 20U11601 | AY566137 | Colombia | 2001 |
| 20W12601 | AY566139 | Colombia | 2001 |
| 20X11601 | AY566140 | Colombia | 2001 |
| 20Y11600 | AY566141 | Colombia | 2000 |
| 20Z13602 | AY566142 | Colombia | 2002 |
| 21A11600 | AY566143 | Colombia | 2000 |
| 21B11602 | AY566144 | Colombia | 2002 |
| 21C13601 | AY566145 | Colombia | 2001 |
| 21D22601 | AY566146 | Colombia | 2001 |
| 21E11600 | AY566147 | Colombia | 2000 |
| 21G11399 | AY781126 | Uruguay | 1999 |
| 21H11399 | AY781127 | Uruguay | 1999 |
| 21K00807 | FJ659675 | Venezuela | 2007 |
| 21L00807 | FJ659677 | Venezuela | 2007 |
| 21M00807 | FJ659679 | Venezuela | 2007 |
| 21N00807 | FJ659681 | Venezuela | 2007 |
| 21U00807 | FJ659688 | Venezuela | 2007 |
| 21V00807 | FJ659689 | Venezuela | 2007 |
| 22A00807 | FJ659695 | Venezuela | 2007 |
| 22G00808 | FJ659778 | Venezuela | 2008 |
| 22K00808 | FJ659770 | Venezuela | 2008 |
| 22M00808 | FJ659766 | Venezuela | 2008 |
| 22P00808 | FJ659761 | Venezuela | 2008 |
| 22Q00808 | FJ659760 | Venezuela | 2008 |
| 22V00808 | FJ659746 | Venezuela | 2008 |
| 22Z00808 | FJ659738 | Venezuela | 2008 |
| 23C00808 | FJ659732 | Venezuela | 2008 |
| 23D00808 | FJ659730 | Venezuela | 2008 |
| 23F00808 | FJ659727 | Venezuela | 2008 |
| 23G00808 | FJ659725 | Venezuela | 2008 |
| 23I00808 | FJ659722 | Venezuela | 2008 |
| 23K00807 | FJ659718 | Venezuela | 2007 |
| 23O00807 | FJ659709 | Venezuela | 2007 |
| 23Q00807 | FJ659706 | Venezuela | 2007 |
| 23R00807 | FJ659705 | Venezuela | 2007 |
| 23V00804 | FJ659839 | Venezuela | 2004 |
| 23W00804 | FJ659838 | Venezuela | 2004 |
| 23Y00807 | FJ659836 | Venezuela | 2007 |
| 23Z00807 | FJ659835 | Venezuela | 2007 |
| 24A00807 | FJ659834 | Venezuela | 2007 |
| 24B00807 | FJ659833 | Venezuela | 2007 |
| 24E00807 | FJ659830 | Venezuela | 2007 |
| 24F00807 | FJ659829 | Venezuela | 2007 |
| 24G00807 | FJ659828 | Venezuela | 2007 |
| 24H00807 | FJ659827 | Venezuela | 2007 |
| 24I00807 | FJ659826 | Venezuela | 2007 |
| 24J00807 | FJ659825 | Venezuela | 2007 |
| 24K00807 | FJ659824 | Venezuela | 2007 |
| 24L00807 | FJ659823 | Venezuela | 2007 |
| 24M00807 | FJ659822 | Venezuela | 2007 |
| 24O00807 | FJ659820 | Venezuela | 2007 |
| 24P00807 | FJ659819 | Venezuela | 2007 |
| 24Q00807 | FJ659818 | Venezuela | 2007 |
| 24R00807 | FJ659817 | Venezuela | 2007 |
| 24S00807 | FJ659816 | Venezuela | 2007 |
| 24T00807 | FJ659815 | Venezuela | 2007 |
| 24U00807 | FJ659814 | Venezuela | 2007 |
| 24W00807 | FJ659812 | Venezuela | 2007 |
| 24X00807 | FJ659811 | Venezuela | 2007 |
| 24Y00807 | FJ659810 | Venezuela | 2007 |
| 25A00807 | FJ659808 | Venezuela | 2007 |
| 25C00807 | FJ659806 | Venezuela | 2007 |
| 25E00807 | FJ659804 | Venezuela | 2007 |
| 25F00807 | FJ659803 | Venezuela | 2007 |
| 25G00805 | FJ659802 | Venezuela | 2005 |
| 25H00805 | FJ659801 | Venezuela | 2005 |
| 25I00805 | FJ659800 | Venezuela | 2005 |
| 25J00805 | FJ659799 | Venezuela | 2005 |
| 25K00805 | FJ659798 | Venezuela | 2005 |
| 25L00805 | FJ659797 | Venezuela | 2005 |
| 25M00805 | FJ659794 | Venezuela | 2005 |
| 25N00805 | FJ659793 | Venezuela | 2005 |
| 25O00805 | FJ659792 | Venezuela | 2005 |
| 25P00805 | FJ659788 | Venezuela | 2005 |
| 25S00808 | FJ659783 | Venezuela | 2008 |
| 25T00808 | FJ659779 | Venezuela | 2008 |
| 25W00808 | FJ659773 | Venezuela | 2008 |
| 25X00808 | FJ659771 | Venezuela | 2008 |
| 25Z00808 | FJ659767 | Venezuela | 2008 |
| 26B00808 | FJ659763 | Venezuela | 2008 |
| 26E00808 | FJ659756 | Venezuela | 2008 |
| 26G00808 | FJ659754 | Venezuela | 2008 |
| 26J00808 | FJ659748 | Venezuela | 2008 |
| 26K00808 | FJ659747 | Venezuela | 2008 |
| 26O00808 | FJ659739 | Venezuela | 2008 |
| 26P00808 | FJ659737 | Venezuela | 2008 |
| 26Q00808 | FJ659735 | Venezuela | 2008 |
| 26R00808 | FJ659733 | Venezuela | 2008 |
| 26S00808 | FJ659731 | Venezuela | 2008 |
| 26U00808 | FJ659726 | Venezuela | 2008 |
| 26V00808 | FJ659723 | Venezuela | 2008 |
| 26W00807 | FJ659721 | Venezuela | 2007 |
| 26X00807 | FJ659719 | Venezuela | 2007 |
| 27C00807 | FJ659701 | Venezuela | 2007 |
| 27D00807 | FJ659698 | Venezuela | 2007 |
| 3XU00N90 | AB485641 | Brazil | 1990 |
| 3XV00N90 | AB485642 | Brazil | 1990 |
| 3XWNNN92 | AF009369 | Brazil | 1992 |
| 3XXNNN92 | AF009370 | Brazil | 1992 |
| 3XYNNN92 | AF009371 | Brazil | 1992 |
| 3XZNNN92 | AF009372 | Brazil | 1992 |
| 3YANNN93 | AF009373 | Brazil | 1993 |
| 3YBNNN92 | AF009374 | Brazil | 1992 |
| 3YCNNN92 | AF009375 | Brazil | 1992 |
| 3YDNNN92 | AF009377 | Brazil | 1992 |
| 3YENNN92 | AF009379 | Brazil | 1992 |
| 3YFNNN92 | AF009380 | Brazil | 1992 |
| 3YGNNN92 | AF009381 | Brazil | 1992 |
| 3YHNNN90 | AF447837 | Brazil | 1990 |
| 3YI00EN | AY145782 | Brazil | N |
| 3YJ00NN | AY145783 | Brazil | N |
| 3YK00NN | AY145784 | Brazil | N |
| 3YL00NN | AY145785 | Brazil | N |
| 3YM00NN | AY145786 | Brazil | N |
| 3YN00NN | AY145787 | Brazil | N |
| 3YO00NN | AY145788 | Brazil | N |
| 3YP00NN | AY145789 | Brazil | N |
| 3YQ00NN | AY145790 | Brazil | N |
| 3YR00NN | AY145791 | Brazil | N |
| 3YS00NN | AY145792 | Brazil | N |
| 3YT00NN | AY145793 | Brazil | N |
| 3YU00NN | AY145794 | Brazil | N |
| 3YV00NN | AY145795 | Brazil | N |
| 3YW00NN | AY145796 | Brazil | N |
| 3YX00NN | AY145797 | Brazil | N |
| 3YY00NN | AY145798 | Brazil | N |
| 3YZ00NN | AY145799 | Brazil | N |
| 3ZA00NN | AY145800 | Brazil | N |
| 3ZB00NN | AY145801 | Brazil | N |
| 3ZC00NN | AY145802 | Brazil | N |
| 3ZD00NN | AY145803 | Brazil | N |
| 3ZE00NN | AY145804 | Brazil | N |
| 3ZF00NN | AY145805 | Brazil | N |
| 3ZG00NN | AY145806 | Brazil | N |
| 3ZH00NN | AY145807 | Brazil | N |
| 3ZI00NN | AY145808 | Brazil | N |
| 3ZJ00NN | AY145809 | Brazil | N |
| 3ZK00NN | AY145810 | Brazil | N |
| 3ZM00NN | AY145812 | Brazil | N |
| 3ZN00NN | AY145814 | Brazil | N |
| 3ZO00NN | AY145815 | Brazil | N |
| 3ZPNNN89 | AY173956 | Brazil | 1989 |
| 3ZQNNN01 | AY213512 | Brazil | 2001 |
| 3ZRNNN01 | AY213513 | Brazil | 2001 |
| 3ZSNNN01 | AY213514 | Brazil | 2001 |
| 3ZUNNN01 | AY213517 | Brazil | 2001 |
| 3ZVNNN01 | AY213524 | Brazil | 2001 |
| 3ZYNNN01 | AY213529 | Brazil | 2001 |
| 3ZZNNN01 | AY213530 | Brazil | 2001 |
| 30ANNN01 | AY213531 | Brazil | 2001 |
| 30BNNN01 | AY213532 | Brazil | 2001 |
| 30CNNN01 | AY213533 | Brazil | 2001 |
| 30ENNN01 | AY213537 | Brazil | 2001 |
| 30FNNN01 | AY213538 | Brazil | 2001 |
| 30GNNN01 | AY213539 | Brazil | 2001 |
| 30INNN01 | AY213541 | Brazil | 2001 |
| 30JNNN01 | AY213543 | Brazil | 2001 |
| 30KNNN01 | AY213545 | Brazil | 2001 |
| 30LNNN01 | AY213546 | Brazil | 2001 |
| 30MNNN01 | AY213547 | Brazil | 2001 |
| 30NNNN01 | AY213548 | Brazil | 2001 |
| 31SNNNN | AY444196 | Brazil | N |
| 31TNNNN | AY444197 | Brazil | N |
| 31UNNNN | AY444198 | Brazil | N |
| 31VNNNN | AY444199 | Brazil | N |
| 31WNNNN | AY444200 | Brazil | N |
| 31XNNNN | AY444201 | Brazil | N |
| 31YNNNN | AY444202 | Brazil | N |
| 31ZNNNN | AY444203 | Brazil | N |
| 32BNNNN | AY444205 | Brazil | N |
| 32CNNNN | AY444206 | Brazil | N |
| 32DNNNN | AY444207 | Brazil | N |
| 32FNND99 | AY771373 | Brazil | 1999 |
| 32HNND99 | AY771375 | Brazil | 1999 |
| 32INND99 | AY771376 | Brazil | 1999 |
| 32JNND99 | AY771377 | Brazil | 1999 |
| 32KNND99 | AY771378 | Brazil | 1999 |
| 32LNND99 | AY771379 | Brazil | 1999 |
| 32MNND99 | AY771382 | Brazil | 1999 |
| 32NNND99 | AY771383 | Brazil | 1999 |
| 32ONND99 | AY771384 | Brazil | 1999 |
| 32PNND99 | AY771385 | Brazil | 1999 |
| 32QNND99 | AY771386 | Brazil | 1999 |
| 32RNND99 | AY771387 | Brazil | 1999 |
| 32SNND99 | AY771389 | Brazil | 1999 |
| 32TNND99 | AY771390 | Brazil | 1999 |
| 32UNND99 | AY771391 | Brazil | 1999 |
| 32VNND99 | AY771392 | Brazil | 1999 |
| 32WNND99 | AY771393 | Brazil | 1999 |
| 32XNND99 | AY771394 | Brazil | 1999 |
| 32YNND99 | AY771395 | Brazil | 1999 |
| 33ANND99 | AY771397 | Brazil | 1999 |
| 33BNND99 | AY771398 | Brazil | 1999 |
| 33CNND99 | AY771399 | Brazil | 1999 |
| 33DNND99 | AY771400 | Brazil | 1999 |
| 33ENND99 | AY771401 | Brazil | 1999 |
| 33FNND99 | AY771402 | Brazil | 1999 |
| 33GNND99 | AY771403 | Brazil | 1999 |
| 33HNND99 | AY771404 | Brazil | 1999 |
| 33INND99 | AY771405 | Brazil | 1999 |
| 33JNND99 | AY771406 | Brazil | 1999 |
| 33KNND99 | AY771407 | Brazil | 1999 |
| 33LNND99 | AY771408 | Brazil | 1999 |
| 33MNND99 | AY771409 | Brazil | 1999 |
| 33NNND99 | AY771411 | Brazil | 1999 |
| 33ONND99 | AY771413 | Brazil | 1999 |
| 33PNND99 | AY771414 | Brazil | 1999 |
| 33QNND99 | AY771415 | Brazil | 1999 |
| 33RNND99 | AY771416 | Brazil | 1999 |
| 33SNND99 | AY771417 | Brazil | 1999 |
| 33TNND99 | AY771419 | Brazil | 1999 |
| 33UNND99 | AY771420 | Brazil | 1999 |
| 33WNND99 | AY771422 | Brazil | 1999 |
| 33XNND99 | AY771423 | Brazil | 1999 |
| 33YNND99 | AY771424 | Brazil | 1999 |
| 33ZNND99 | AY771425 | Brazil | 1999 |
| 34ANND99 | AY771426 | Brazil | 1999 |
| 34BNND99 | AY771427 | Brazil | 1999 |
| 34CNND99 | AY771428 | Brazil | 1999 |
| 34DNND99 | AY771429 | Brazil | 1999 |
| 34ENND99 | AY771430 | Brazil | 1999 |
| 34FNND99 | AY771432 | Brazil | 1999 |
| 34GNND99 | AY771433 | Brazil | 1999 |
| 34HNND99 | AY771434 | Brazil | 1999 |
| 34INND99 | AY771435 | Brazil | 1999 |
| 34JNND99 | AY771436 | Brazil | 1999 |
| 34KNND99 | AY771437 | Brazil | 1999 |
| 34LNND99 | AY771438 | Brazil | 1999 |
| 34MNND99 | AY771440 | Brazil | 1999 |
| 34NNND99 | AY771441 | Brazil | 1999 |
| 34ONND99 | AY771442 | Brazil | 1999 |
| 34PNND99 | AY771443 | Brazil | 1999 |
| 34QNND99 | AY771444 | Brazil | 1999 |
| 34RNND99 | AY771445 | Brazil | 1999 |
| 34SNND99 | AY771446 | Brazil | 1999 |
| 34TNND99 | AY771448 | Brazil | 1999 |
| 34UNND99 | AY771449 | Brazil | 1999 |
| 34VNND99 | AY771450 | Brazil | 1999 |
| 34WNND99 | AY771451 | Brazil | 1999 |
| 34XNND99 | AY771452 | Brazil | 1999 |
| 34YNND99 | AY771453 | Brazil | 1999 |
| 34ZNND99 | AY771455 | Brazil | 1999 |
| 35ANND99 | AY771456 | Brazil | 1999 |
| 35BNND99 | AY771458 | Brazil | 1999 |
| 35CNND99 | AY771461 | Brazil | 1999 |
| 35DNND99 | AY771462 | Brazil | 1999 |
| 35ENND99 | AY771463 | Brazil | 1999 |
| 35FNND99 | AY771464 | Brazil | 1999 |
| 35GNND99 | AY771465 | Brazil | 1999 |
| 35HNND99 | AY771466 | Brazil | 1999 |
| 35INND99 | AY771467 | Brazil | 1999 |
| 35JNND99 | AY771468 | Brazil | 1999 |
| 35KNND99 | AY771469 | Brazil | 1999 |
| 35LNND99 | AY771470 | Brazil | 1999 |
| 35MNND99 | AY771472 | Brazil | 1999 |
| 35NNND99 | AY771473 | Brazil | 1999 |
| 35ONND99 | AY771474 | Brazil | 1999 |
| 35PNND99 | AY771475 | Brazil | 1999 |
| 35QNND99 | AY771476 | Brazil | 1999 |
| 35RNND99 | AY771477 | Brazil | 1999 |
| 35SNND99 | AY771478 | Brazil | 1999 |
| 35TNND99 | AY771479 | Brazil | 1999 |
| 35UNND99 | AY771480 | Brazil | 1999 |
| 35VNND99 | AY771481 | Brazil | 1999 |
| 35WNND99 | AY771483 | Brazil | 1999 |
| 35XNND99 | AY771485 | Brazil | 1999 |
| 35YNND99 | AY771486 | Brazil | 1999 |
| 35ZNND99 | AY771487 | Brazil | 1999 |
| 36ANND99 | AY771488 | Brazil | 1999 |
| 36BNND99 | AY771490 | Brazil | 1999 |
| 36CNND99 | AY771492 | Brazil | 1999 |
| 36DNND99 | AY771493 | Brazil | 1999 |
| 36ENND99 | AY771494 | Brazil | 1999 |
| 36FNND99 | AY771495 | Brazil | 1999 |
| 36GNND99 | AY771496 | Brazil | 1999 |
| 36HNND99 | AY771497 | Brazil | 1999 |
| 36INND99 | AY771498 | Brazil | 1999 |
| 36JNND99 | AY771499 | Brazil | 1999 |
| 36KNND99 | AY771500 | Brazil | 1999 |
| 36MNND99 | AY771502 | Brazil | 1999 |
| 36NNND99 | AY771503 | Brazil | 1999 |
| 36ONND99 | AY771504 | Brazil | 1999 |
| 36PNND99 | AY771505 | Brazil | 1999 |
| 36QNND99 | AY771506 | Brazil | 1999 |
| 36RNND99 | AY771507 | Brazil | 1999 |
| 36SNND99 | AY771508 | Brazil | 1999 |
| 36TNND99 | AY771509 | Brazil | 1999 |
| 36UNND99 | AY771511 | Brazil | 1999 |
| 36VNND99 | AY771512 | Brazil | 1999 |
| 36WNND99 | AY771514 | Brazil | 1999 |
| 36XNND99 | AY771515 | Brazil | 1999 |
| 36YNND99 | AY771516 | Brazil | 1999 |
| 36ZNND99 | AY771517 | Brazil | 1999 |
| 37ANND99 | AY771518 | Brazil | 1999 |
| 37BNND99 | AY771519 | Brazil | 1999 |
| 37CNND99 | AY771522 | Brazil | 1999 |
| 37DNND99 | AY771525 | Brazil | 1999 |
| 37ENND99 | AY771526 | Brazil | 1999 |
| 37FNND99 | AY771527 | Brazil | 1999 |
| 37GNND99 | AY771528 | Brazil | 1999 |
| 37INND99 | AY771530 | Brazil | 1999 |
| 37JNND99 | AY771531 | Brazil | 1999 |
| 37KNND99 | AY771532 | Brazil | 1999 |
| 37LNND99 | AY771533 | Brazil | 1999 |
| 37MNND99 | AY771535 | Brazil | 1999 |
| 37NNND99 | AY771537 | Brazil | 1999 |
| 37ONND99 | AY771538 | Brazil | 1999 |
| 37PNND99 | AY771539 | Brazil | 1999 |
| 37QNND99 | AY771540 | Brazil | 1999 |
| 37RNND99 | AY771541 | Brazil | 1999 |
| 37SNND99 | AY771542 | Brazil | 1999 |
| 37TNND99 | AY771543 | Brazil | 1999 |
| 37UNND99 | AY771544 | Brazil | 1999 |
| 37VNND99 | AY771545 | Brazil | 1999 |
| 37WNND99 | AY771546 | Brazil | 1999 |
| 37XNND99 | AY771547 | Brazil | 1999 |
| 37YNND99 | AY771548 | Brazil | 1999 |
| 37ZNND99 | AY771550 | Brazil | 1999 |
| 38BNND99 | AY771552 | Brazil | 1999 |
| 38DNND99 | AY771555 | Brazil | 1999 |
| 38ENND99 | AY771556 | Brazil | 1999 |
| 38FNND99 | AY771557 | Brazil | 1999 |
| 38GNND99 | AY771558 | Brazil | 1999 |
| 38HNND99 | AY771559 | Brazil | 1999 |
| 38INND99 | AY771560 | Brazil | 1999 |
| 38JNND99 | AY771561 | Brazil | 1999 |
| 38KNND99 | AY771563 | Brazil | 1999 |
| 38LNND99 | AY771564 | Brazil | 1999 |
| 38MNND99 | AY771565 | Brazil | 1999 |
| 38NNND99 | AY771566 | Brazil | 1999 |
| 38ONND99 | AY771567 | Brazil | 1999 |
| 38PNND99 | AY771568 | Brazil | 1999 |
| 38QNND99 | AY771569 | Brazil | 1999 |
| 38SNND99 | AY771571 | Brazil | 1999 |
| 38TNND99 | AY771572 | Brazil | 1999 |
| 38VNND99 | AY771575 | Brazil | 1999 |
| 38WNND99 | AY771576 | Brazil | 1999 |
| 38XNND99 | AY771578 | Brazil | 1999 |
| 38YNND99 | AY771579 | Brazil | 1999 |
| 38ZNND99 | AY771580 | Brazil | 1999 |
| 39ANND99 | AY771581 | Brazil | 1999 |
| 39BNND99 | AY771582 | Brazil | 1999 |
| 39CNND99 | AY771584 | Brazil | 1999 |
| 39DNND99 | AY771585 | Brazil | 1999 |
| 39ENND99 | AY771587 | Brazil | 1999 |
| 4BXNND98 | DQ003015 | Brazil | 1998 |
| 4BZ04EN | DQ156234 | Brazil | N |
| 4CA04EN | DQ156235 | Brazil | N |
| 4CF04EN | DQ156241 | Brazil | N |
| 4CG04EN | DQ156242 | Brazil | N |
| 4CH04EN | DQ156243 | Brazil | N |
| 4CK04EN | DQ156246 | Brazil | N |
| 4CM04EN | DQ156248 | Brazil | N |
| 4CQ04EN | DQ156253 | Brazil | N |
| 4CT04EN | DQ156256 | Brazil | N |
| 4DD04EN | DQ156268 | Brazil | N |
| 4DE04EN | DQ156269 | Brazil | N |
| 4DF04EN | DQ156272 | Brazil | N |
| 4DG04EN | DQ156273 | Brazil | N |
| 4DH04EN | DQ156274 | Brazil | N |
| 4DI04EN | DQ156275 | Brazil | N |
| 4DJ04EN | DQ156276 | Brazil | N |
| 4DK04EN | DQ156278 | Brazil | N |
| 4DN04EN | DQ156282 | Brazil | N |
| 4DP04EN | DQ156284 | Brazil | N |
| 4DQ04EN | DQ156285 | Brazil | N |
| 4DR04EN | DQ156286 | Brazil | N |
| 4DS04EN | DQ156290 | Brazil | N |
| 4DT04EN | DQ156291 | Brazil | N |
| 4DU04EN | DQ156293 | Brazil | N |
| 4DV04EN | DQ156294 | Brazil | N |
| 4DW04EN | DQ156295 | Brazil | N |
| 4DX04EN | DQ156296 | Brazil | N |
| 4DYNNA02 | DQ343965 | Brazil | 2002 |
| 4DZNNA02 | DQ343966 | Brazil | 2002 |
| 4EANNA02 | DQ343967 | Brazil | 2002 |
| 4EBNNA02 | DQ343972 | Brazil | 2002 |
| 4EENNA02 | DQ344017 | Brazil | 2002 |
| 4EFN2N04 | DQ518441 | Brazil | 2004 |
| 4EGN2N04 | DQ518442 | Brazil | 2004 |
| 4EHN2N04 | DQ518443 | Brazil | 2004 |
| 4EIN2N04 | DQ518445 | Brazil | 2004 |
| 4EJN2N04 | DQ518446 | Brazil | 2004 |
| 4EKN2N04 | DQ518447 | Brazil | 2004 |
| 4ELN2N04 | DQ518448 | Brazil | 2004 |
| 4ENN2N04 | DQ518450 | Brazil | 2004 |
| 4EON2N04 | DQ518451 | Brazil | 2004 |
| 4EPN2N04 | DQ518452 | Brazil | 2004 |
| 4EQN2N04 | DQ518453 | Brazil | 2004 |
| 4ERN2N04 | DQ518454 | Brazil | 2004 |
| 4ESN2N04 | DQ518455 | Brazil | 2004 |
| 4ETN2N04 | DQ518456 | Brazil | 2004 |
| 4EUN2N04 | DQ518458 | Brazil | 2004 |
| 4EVN2N04 | DQ518459 | Brazil | 2004 |
| 4EXN2N04 | DQ518462 | Brazil | 2004 |
| 4EYN2N04 | DQ518463 | Brazil | 2004 |
| 4EZN2N04 | DQ518464 | Brazil | 2004 |
| 4FAN2N04 | DQ518465 | Brazil | 2004 |
| 4FBN2N04 | DQ518466 | Brazil | 2004 |
| 4FCN2N04 | DQ518467 | Brazil | 2004 |
| 4FDN2N04 | DQ518468 | Brazil | 2004 |
| 4FEN2N04 | DQ518471 | Brazil | 2004 |
| 4FGN2N04 | DQ518473 | Brazil | 2004 |
| 4FHN2N04 | DQ518474 | Brazil | 2004 |
| 4FIN2N04 | DQ518475 | Brazil | 2004 |
| 4FJN2N04 | DQ518476 | Brazil | 2004 |
| 4FKN2N04 | DQ518477 | Brazil | 2004 |
| 4FLN2N04 | DQ518478 | Brazil | 2004 |
| 4FMN2N04 | DQ518480 | Brazil | 2004 |
| 4FNN2N04 | DQ518481 | Brazil | 2004 |
| 4FON2N04 | DQ518482 | Brazil | 2004 |
| 4FPN2N04 | DQ518485 | Brazil | 2004 |
| 4FQN2N04 | DQ518487 | Brazil | 2004 |
| 4FRN2N04 | DQ518488 | Brazil | 2004 |
| 4FSN2N04 | DQ518529 | Brazil | 2004 |
| 4FTN2N04 | DQ518530 | Brazil | 2004 |
| 4FUN2N04 | DQ518534 | Brazil | 2004 |
| 4FVN2N04 | DQ518535 | Brazil | 2004 |
| 4FWN2N04 | DQ518542 | Brazil | 2004 |
| 4FYN2N04 | DQ518546 | Brazil | 2004 |
| 4FZN2N04 | DQ518547 | Brazil | 2004 |
| 4GANNE02 | DQ899643 | Brazil | 2002 |
| 4GBNNE03 | DQ899644 | Brazil | 2003 |
| 4GCNNE03 | DQ899645 | Brazil | 2003 |
| 4GDNNE03 | DQ899646 | Brazil | 2003 |
| 4GENNE03 | DQ899647 | Brazil | 2003 |
| 4GFNNE03 | DQ899649 | Brazil | 2003 |
| 4GGNNE03 | DQ899650 | Brazil | 2003 |
| 4GHNNE03 | DQ899651 | Brazil | 2003 |
| 4GINNE03 | DQ899652 | Brazil | 2003 |
| 4GJNNE04 | DQ899653 | Brazil | 2004 |
| 4GKNNE04 | DQ899654 | Brazil | 2004 |
| 4GLNNE04 | DQ899655 | Brazil | 2004 |
| 4GMNNE04 | DQ899656 | Brazil | 2004 |
| 4GNNNE04 | DQ899657 | Brazil | 2004 |
| 4GONNE04 | DQ899658 | Brazil | 2004 |
| 4GQNNE04 | DQ899660 | Brazil | 2004 |
| 4GRNNE04 | DQ899661 | Brazil | 2004 |
| 4GSNNE04 | DQ899662 | Brazil | 2004 |
| 4GTNNE05 | DQ899663 | Brazil | 2005 |
| 4GUNNE05 | DQ899664 | Brazil | 2005 |
| 4GVNNE05 | DQ899665 | Brazil | 2005 |
| 4GWNNE05 | DQ899666 | Brazil | 2005 |
| 4GXNNE05 | DQ899667 | Brazil | 2005 |
| 4GYNNE05 | DQ899668 | Brazil | 2005 |
| 4GZNNE05 | DQ899670 | Brazil | 2005 |
| 4HANNE05 | DQ899672 | Brazil | 2005 |
| 4HBNNE05 | DQ899673 | Brazil | 2005 |
| 4HCNNE05 | DQ899674 | Brazil | 2005 |
| 4HDNNE05 | DQ899675 | Brazil | 2005 |
| 4HENNE05 | DQ899676 | Brazil | 2005 |
| 4HFNNE05 | DQ899678 | Brazil | 2005 |
| 4HGNNE05 | DQ899679 | Brazil | 2005 |
| 4HHNNE05 | DQ899680 | Brazil | 2005 |
| 4HINNE05 | DQ899681 | Brazil | 2005 |
| 4HJNNE03 | DQ899682 | Brazil | 2003 |
| 4HKNNE03 | DQ899683 | Brazil | 2003 |
| 4HLNNE04 | DQ899687 | Brazil | 2004 |
| 4HMNNE04 | DQ899688 | Brazil | 2004 |
| 4HNNNE04 | DQ899689 | Brazil | 2004 |
| 4HONNE04 | DQ899690 | Brazil | 2004 |
| 4HPNNE05 | DQ899691 | Brazil | 2005 |
| 4HQNNE05 | DQ899692 | Brazil | 2005 |
| 4HRNNE05 | DQ899693 | Brazil | 2005 |
| 4HSNNE05 | DQ899694 | Brazil | 2005 |
| 4HTNNE05 | DQ899695 | Brazil | 2005 |
| 4HUNNE05 | DQ899696 | Brazil | 2005 |
| 4HWNNE04 | DQ899699 | Brazil | 2004 |
| 4HXNNE04 | DQ899700 | Brazil | 2004 |
| 4HZNNE04 | DQ899702 | Brazil | 2004 |
| 4IANNE04 | DQ899703 | Brazil | 2004 |
| 4IBNNE04 | DQ899705 | Brazil | 2004 |
| 4IENNE04 | DQ899712 | Brazil | 2004 |
| 4IGNNE05 | DQ899714 | Brazil | 2005 |
| 4IHNNE05 | DQ899715 | Brazil | 2005 |
| 4IINNE05 | DQ899716 | Brazil | 2005 |
| 4IJNNE06 | DQ899717 | Brazil | 2006 |
| 4IKNNE06 | DQ899718 | Brazil | 2006 |
| 4ILNNE06 | DQ899719 | Brazil | 2006 |
| 4IMNNE06 | DQ899720 | Brazil | 2006 |
| 4INNNE04 | DQ899721 | Brazil | 2004 |
| 4IONNE05 | DQ899722 | Brazil | 2005 |
| 4IPNNE05 | DQ899723 | Brazil | 2005 |
| 4IRNNE05 | DQ899725 | Brazil | 2005 |
| 4ISNNE05 | DQ899726 | Brazil | 2005 |
| 4ITNNE04 | DQ899727 | Brazil | 2004 |
| 4IUNNE04 | EF042627 | Brazil | 2004 |
| 4IVNNE04 | EF042628 | Brazil | 2004 |
| 4IWNNE04 | EF042630 | Brazil | 2004 |
| 4IXNNE04 | EF042631 | Brazil | 2004 |
| 4IYNNE06 | EF042632 | Brazil | 2006 |
| 4IZNNE04 | EF042633 | Brazil | 2004 |
| 4JANNE05 | EF042634 | Brazil | 2005 |
| 4JBNNE04 | EF042635 | Brazil | 2004 |
| 4JCNNE04 | EF042636 | Brazil | 2004 |
| 4JDNNE06 | EF042637 | Brazil | 2006 |
| 4JENNE06 | EF042638 | Brazil | 2006 |
| 4JFNNE06 | EF042639 | Brazil | 2006 |
| 4JGNNE04 | EF042640 | Brazil | 2004 |
| 4JHNNE05 | EF042641 | Brazil | 2005 |
| 4JKNNE05 | EF042645 | Brazil | 2005 |
| 4JMNNE05 | EF042649 | Brazil | 2005 |
| 4JONNE04 | EF042653 | Brazil | 2004 |
| 4JQNNE04 | EF042655 | Brazil | 2004 |
| 4JRNNE05 | EF042656 | Brazil | 2005 |
| 4JSNNE04 | EF042659 | Brazil | 2004 |
| 4JVNNE04 | EF042662 | Brazil | 2004 |
| 4JXNNE05 | EF042665 | Brazil | 2005 |
| 4JYNNE05 | EF042666 | Brazil | 2005 |
| 4JZNNE05 | EF042668 | Brazil | 2005 |
| 4KANNE05 | EF042669 | Brazil | 2005 |
| 4KBNNE05 | EF042670 | Brazil | 2005 |
| 4KDNNE05 | EF042672 | Brazil | 2005 |
| 4KENNE05 | EF042673 | Brazil | 2005 |
| 4KFNNE05 | EF042674 | Brazil | 2005 |
| 4KGNNE05 | EF042675 | Brazil | 2005 |
| 4KHNNE05 | EF042676 | Brazil | 2005 |
| 4KJNNE06 | EF042678 | Brazil | 2006 |
| 4KKNNE05 | EF042679 | Brazil | 2005 |
| 4KMNNE05 | EF042681 | Brazil | 2005 |
| 4KNNNE06 | EF042682 | Brazil | 2006 |
| 4KONNE06 | EF042683 | Brazil | 2006 |
| 4KQNNE06 | EF042689 | Brazil | 2006 |
| 4KRNNE06 | EF042690 | Brazil | 2006 |
| 4KUNNNN | EF075952 | Brazil | N |
| 4KVNNNN | EF075953 | Brazil | N |
| 4KWNNNN | EF075954 | Brazil | N |
| 4KXNNNN | EF075956 | Brazil | N |
| 4KYNNNN | EF075957 | Brazil | N |
| 4KZNNNN | EF075958 | Brazil | N |
| 4LANNNN | EF075959 | Brazil | N |
| 4LBNNNN | EF075960 | Brazil | N |
| 4LDNNNN | EF075962 | Brazil | N |
| 4LENNNN | EF075964 | Brazil | N |
| 4MANND04 | FJ195086 | Brazil | 2004 |
| 4MBNND05 | FJ195088 | Brazil | 2005 |
| 4MCNND05 | FJ195089 | Brazil | 2005 |
| 4MDNND04 | FJ195090 | Brazil | 2004 |
| 4MENND05 | FJ195091 | Brazil | 2005 |
| 4MGNNN05 | FJ405060 | Brazil | 2005 |
| 4MINNN05 | FJ405064 | Brazil | 2005 |
| 4MJNNN05 | FJ405067 | Brazil | 2005 |
| 4MKNNN05 | FJ405071 | Brazil | 2005 |
| 4MLNNN05 | FJ405075 | Brazil | 2005 |
| 4MMNNN05 | FJ405080 | Brazil | 2005 |
| 4MNNNN05 | FJ405082 | Brazil | 2005 |
| 4MPNNN05 | FJ405091 | Brazil | 2005 |
| 4MQNNN05 | FJ405094 | Brazil | 2005 |
| 4MRNNN05 | FJ405098 | Brazil | 2005 |
| 4MUNNN05 | FJ405101 | Brazil | 2005 |
| 4MVNNN05 | FJ405102 | Brazil | 2005 |
| 4MXNNN05 | FJ405104 | Brazil | 2005 |
| 4MZNNN05 | FJ405106 | Brazil | 2005 |
| 4NANNN05 | FJ405108 | Brazil | 2005 |
| 4NBNNN05 | FJ405109 | Brazil | 2005 |
| 4NCNNN05 | FJ405110 | Brazil | 2005 |
| 4NDNNN02 | FJ405111 | Brazil | 2002 |
| 4NENNN05 | FJ405112 | Brazil | 2005 |
| 4NFNNN05 | FJ405113 | Brazil | 2005 |
| 4NGNNN05 | FJ405114 | Brazil | 2005 |
| 4NINNN05 | FJ405117 | Brazil | 2005 |
| 4NJ00E06 | FJ591163 | Brazil | 2006 |
| 4NK00E06 | FJ591165 | Brazil | 2006 |
| 4NL00E06 | FJ591166 | Brazil | 2006 |
| 4NM00E06 | FJ591167 | Brazil | 2006 |
| 4NN00E06 | FJ591170 | Brazil | 2006 |
| 4NO00E06 | FJ591171 | Brazil | 2006 |
| 4NP00E06 | FJ591173 | Brazil | 2006 |
| 4NQ00E06 | FJ591174 | Brazil | 2006 |
| 4NR00E06 | FJ591175 | Brazil | 2006 |
| 4NS00N04 | FJ591177 | Brazil | 2004 |
| 4NT00N04 | FJ591178 | Brazil | 2004 |
| 4NU00N04 | FJ591179 | Brazil | 2004 |
| 4NV00N04 | FJ591182 | Brazil | 2004 |
| 4NW00N04 | FJ591183 | Brazil | 2004 |
| 4NX00N04 | FJ591185 | Brazil | 2004 |
| 4NY00N04 | FJ591186 | Brazil | 2004 |
| 4OA00N06 | FJ591189 | Brazil | 2006 |
| 4OB00N08 | FJ591191 | Brazil | 2008 |
| 4OC00N04 | FJ591192 | Brazil | 2004 |
| 4OD00N04 | FJ591197 | Brazil | 2004 |
| 4OE00N04 | FJ591198 | Brazil | 2004 |
| 4OF00N04 | FJ591199 | Brazil | 2004 |
| 4OG00N04 | FJ591200 | Brazil | 2004 |
| 4OH00N04 | FJ591201 | Brazil | 2004 |
| 4OI00N04 | FJ591202 | Brazil | 2004 |
| 4OK00N04 | FJ591206 | Brazil | 2004 |
| 4OM00N04 | FJ591211 | Brazil | 2004 |
| 4ON00N04 | FJ591220 | Brazil | 2004 |
| 4OO00N04 | FJ591221 | Brazil | 2004 |
| 4OP00N04 | FJ591222 | Brazil | 2004 |
| 4OQ00N04 | FJ591224 | Brazil | 2004 |
| 4OR00N04 | FJ591226 | Brazil | 2004 |
| 4OS00N04 | FJ591227 | Brazil | 2004 |
| 4OT00N04 | FJ591228 | Brazil | 2004 |
| 4OU00N04 | FJ591230 | Brazil | 2004 |
| 4OV00N04 | FJ591231 | Brazil | 2004 |
| 4OW00N04 | FJ591233 | Brazil | 2004 |
| 4OX00N04 | FJ591234 | Brazil | 2004 |
| 4OY00N04 | FJ591235 | Brazil | 2004 |
| 4OZ00N04 | FJ591239 | Brazil | 2004 |
| 4PA00N04 | FJ591240 | Brazil | 2004 |
| 4PB00N04 | FJ591243 | Brazil | 2004 |
| 4PC00N04 | FJ591244 | Brazil | 2004 |
| 4PD00N04 | FJ591246 | Brazil | 2004 |
| 4PE00N04 | FJ591247 | Brazil | 2004 |
| 4PF00N05 | FJ591250 | Brazil | 2005 |
| 4PG00N05 | FJ591251 | Brazil | 2005 |
| 4PH00N05 | FJ591252 | Brazil | 2005 |
| 4PI00N05 | FJ591253 | Brazil | 2005 |
| 4PJ00N05 | FJ591254 | Brazil | 2005 |
| 4PK00N05 | FJ591256 | Brazil | 2005 |
| 4PM00N05 | FJ591258 | Brazil | 2005 |
| 4PN00N05 | FJ591259 | Brazil | 2005 |
| 4PO00N05 | FJ591261 | Brazil | 2005 |
| 4PP00N05 | FJ591262 | Brazil | 2005 |
| 4PQ00N05 | FJ591263 | Brazil | 2005 |
| 4PR00N05 | FJ591266 | Brazil | 2005 |
| 4PS00N05 | FJ591267 | Brazil | 2005 |
| 4PT00N05 | FJ591269 | Brazil | 2005 |
| 4PV00N05 | FJ591272 | Brazil | 2005 |
| 4PW00N05 | FJ591273 | Brazil | 2005 |
| 4PX00N05 | FJ591274 | Brazil | 2005 |
| 4PY00N05 | FJ591275 | Brazil | 2005 |
| 4QA00N05 | FJ591278 | Brazil | 2005 |
| 4QB00N05 | FJ591280 | Brazil | 2005 |
| 4QC00N05 | FJ591284 | Brazil | 2005 |
| 4QD00N05 | FJ591285 | Brazil | 2005 |
| 4QE00N05 | FJ591286 | Brazil | 2005 |
| 4QF00N05 | FJ591291 | Brazil | 2005 |
| 4QI00N05 | FJ591297 | Brazil | 2005 |
| 4QK00N05 | FJ591300 | Brazil | 2005 |
| 4QL00N05 | FJ591301 | Brazil | 2005 |
| 4QM00N05 | FJ591304 | Brazil | 2005 |
| 4QN00N05 | FJ591305 | Brazil | 2005 |
| 4QP00N05 | FJ591308 | Brazil | 2005 |
| 4QQ00N05 | FJ591309 | Brazil | 2005 |
| 4QR00N05 | FJ591310 | Brazil | 2005 |
| 4QS00N05 | FJ591311 | Brazil | 2005 |
| 4QT00N05 | FJ591312 | Brazil | 2005 |
| 4QU00N05 | FJ591313 | Brazil | 2005 |
| 4QV00N05 | FJ591314 | Brazil | 2005 |
| 4QW00N05 | FJ591319 | Brazil | 2005 |
| 4QX00N05 | FJ591321 | Brazil | 2005 |
| 4QY00N05 | FJ591322 | Brazil | 2005 |
| 4QZ00N05 | FJ591323 | Brazil | 2005 |
| 4RA00N05 | FJ591328 | Brazil | 2005 |
| 4RC00N05 | FJ591330 | Brazil | 2005 |
| 4RD00N05 | FJ591332 | Brazil | 2005 |
| 4RE00N05 | FJ591333 | Brazil | 2005 |
| 4RF00N05 | FJ591336 | Brazil | 2005 |
| 4RG00N05 | FJ591337 | Brazil | 2005 |
| 4RH00N05 | FJ591340 | Brazil | 2005 |
| 4RI00N05 | FJ591343 | Brazil | 2005 |
| 4RJ00N05 | FJ591345 | Brazil | 2005 |
| 4RK00N05 | FJ591346 | Brazil | 2005 |
| 4RL00N05 | FJ591348 | Brazil | 2005 |
| 4RM00N05 | FJ591349 | Brazil | 2005 |
| 4RO00N05 | FJ591351 | Brazil | 2005 |
| 4RP00N05 | FJ591354 | Brazil | 2005 |
| 4RQ00N05 | FJ591356 | Brazil | 2005 |
| 4RR00N05 | FJ591357 | Brazil | 2005 |
| 4RS00N05 | FJ591358 | Brazil | 2005 |
| 4RT00N05 | FJ591361 | Brazil | 2005 |
| 4RU00N05 | FJ591365 | Brazil | 2005 |
| 4RV00N05 | FJ591366 | Brazil | 2005 |
| 4RW00N05 | FJ591369 | Brazil | 2005 |
| 4RY00N05 | FJ591372 | Brazil | 2005 |
| 4RZ00N05 | FJ591373 | Brazil | 2005 |
| 4SA00N05 | FJ591375 | Brazil | 2005 |
| 4SB00N05 | FJ591378 | Brazil | 2005 |
| 4SC00N05 | FJ591379 | Brazil | 2005 |
| 4SE00N05 | FJ591382 | Brazil | 2005 |
| 4SF00N05 | FJ591383 | Brazil | 2005 |
| 4SG00N05 | FJ591384 | Brazil | 2005 |
| 4SH00N05 | FJ591391 | Brazil | 2005 |
| 4SI00N05 | FJ591394 | Brazil | 2005 |
| 4SJ00N05 | FJ591395 | Brazil | 2005 |
| 4SK00N05 | FJ591396 | Brazil | 2005 |
| 4SL00N05 | FJ591398 | Brazil | 2005 |
| 4SN00N05 | FJ591400 | Brazil | 2005 |
| 4SO00N05 | fJ591401 | Brazil | 2005 |
| 4SP00N05 | FJ591405 | Brazil | 2005 |
| 4SQ00N05 | FJ591407 | Brazil | 2005 |
| 4SR00N05 | FJ591408 | Brazil | 2005 |
| 4ST00N05 | FJ591415 | Brazil | 2005 |
| 4SU00N05 | FJ591419 | Brazil | 2005 |
| 4SV00N05 | FJ591421 | Brazil | 2005 |
| 4SW00N05 | FJ591422 | Brazil | 2005 |
| 4SY00N05 | FJ591426 | Brazil | 2005 |
| 4SZ00N05 | FJ591427 | Brazil | 2005 |
| 4TB00N05 | FJ591429 | Brazil | 2005 |
| 4TC00N05 | FJ591432 | Brazil | 2005 |
| 4TD00N05 | FJ591433 | Brazil | 2005 |
| 4TE00N05 | FJ591434 | Brazil | 2005 |
| 4TF00N05 | FJ591435 | Brazil | 2005 |
| 4TG00N05 | FJ591438 | Brazil | 2005 |
| 4TH00N05 | FJ591442 | Brazil | 2005 |
| 4TJ00N05 | FJ591445 | Brazil | 2005 |
| 4TK00N05 | FJ591446 | Brazil | 2005 |
| 4TL00N05 | FJ591447 | Brazil | 2005 |
| 4TM00N05 | FJ591448 | Brazil | 2005 |
| 4TN00N05 | FJ591449 | Brazil | 2005 |
| 4TO00N05 | FJ591450 | Brazil | 2005 |
| 4TP00N06 | FJ591452 | Brazil | 2006 |
| 4TR00N06 | FJ591454 | Brazil | 2006 |
| 4TS00N06 | FJ591457 | Brazil | 2006 |
| 4TT00N06 | FJ591459 | Brazil | 2006 |
| 4TU00N06 | FJ591463 | Brazil | 2006 |
| 4TV00N06 | FJ591465 | Brazil | 2006 |
| 4TW00N06 | FJ591467 | Brazil | 2006 |
| 4TX00N06 | FJ591471 | Brazil | 2006 |
| 4TY00N06 | FJ591472 | Brazil | 2006 |
| 4TZ00N06 | FJ591473 | Brazil | 2006 |
| 4UA00N06 | FJ591474 | Brazil | 2006 |
| 4UB00N06 | FJ591477 | Brazil | 2006 |
| 4UC00N06 | FJ591480 | Brazil | 2006 |
| 4UD00N06 | FJ591482 | Brazil | 2006 |
| 4UE00N06 | FJ591484 | Brazil | 2006 |
| 4UF00N06 | FJ591487 | Brazil | 2006 |
| 4UG00N06 | FJ591488 | Brazil | 2006 |
| 4UH00N06 | FJ591490 | Brazil | 2006 |
| 4UI00N06 | FJ591491 | Brazil | 2006 |
| 4UJ00N06 | FJ591492 | Brazil | 2006 |
| 4UK00N06 | FJ591493 | Brazil | 2006 |
| 4UL00N06 | FJ591494 | Brazil | 2006 |
| 4UM00N06 | FJ591496 | Brazil | 2006 |
| 4UO00N06 | FJ591498 | Brazil | 2006 |
| 4UP00N06 | FJ591500 | Brazil | 2006 |
| 4UQ00N06 | FJ591501 | Brazil | 2006 |
| 4UR00N06 | FJ591504 | Brazil | 2006 |
| 4US00N06 | FJ591505 | Brazil | 2006 |
| 4UT00N06 | FJ591508 | Brazil | 2006 |
| 4UU00N06 | FJ591510 | Brazil | 2006 |
| 4UV00N06 | FJ591512 | Brazil | 2006 |
| 4UW00N06 | FJ591513 | Brazil | 2006 |
| 4UY00N06 | FJ591515 | Brazil | 2006 |
| 4UZ00N06 | FJ591516 | Brazil | 2006 |
| 4WA00N06 | FJ591517 | Brazil | 2006 |
| 4WC00N06 | FJ591519 | Brazil | 2006 |
| 4WE00N06 | FJ591522 | Brazil | 2006 |
| 4WF00N06 | FJ591525 | Brazil | 2006 |
| 4WG00N06 | FJ591528 | Brazil | 2006 |
| 4WH00N06 | FJ591532 | Brazil | 2006 |
| 4WI00N06 | FJ591533 | Brazil | 2006 |
| 4WJ00N06 | FJ591534 | Brazil | 2006 |
| 4WK00N06 | FJ591535 | Brazil | 2006 |
| 4WL00N06 | FJ591536 | Brazil | 2006 |
| 4WM00N06 | FJ591537 | Brazil | 2006 |
| 4WO00N06 | FJ591542 | Brazil | 2006 |
| 4WP00N06 | FJ591543 | Brazil | 2006 |
| 4WQ00N06 | FJ591544 | Brazil | 2006 |
| 4WR00N06 | FJ591546 | Brazil | 2006 |
| 4WS00N06 | FJ591547 | Brazil | 2006 |
| 4WT00N06 | FJ591549 | Brazil | 2006 |
| 4WU00N06 | FJ591553 | Brazil | 2006 |
| 4WV00N06 | FJ591556 | Brazil | 2006 |
| 4WW00N04 | FJ591558 | Brazil | 2004 |
| 4WX00N04 | FJ591562 | Brazil | 2004 |
| 4WY00N04 | FJ591565 | Brazil | 2004 |
| 4WZ00N04 | FJ591566 | Brazil | 2004 |
| 4XA00N04 | FJ591567 | Brazil | 2004 |
| 4XB00N04 | FJ591568 | Brazil | 2004 |
| 4XC00N04 | FJ591569 | Brazil | 2004 |
| 4XD00N04 | FJ591570 | Brazil | 2004 |
| 4XE00N04 | FJ591572 | Brazil | 2004 |
| 4XF00N04 | FJ591573 | Brazil | 2004 |
| 4XG00N04 | FJ591575 | Brazil | 2004 |
| 4XH00N04 | FJ591576 | Brazil | 2004 |
| 4XI00N04 | FJ591577 | Brazil | 2004 |
| 4XJ00N04 | FJ591581 | Brazil | 2004 |
| 4XK00N04 | FJ591582 | Brazil | 2004 |
| 4XL00N04 | FJ591583 | Brazil | 2004 |
| 4XM00N04 | FJ591584 | Brazil | 2004 |
| 4XN00N04 | FJ591585 | Brazil | 2004 |
| 4XO00N04 | FJ591586 | Brazil | 2004 |
| 4XP00N04 | FJ591587 | Brazil | 2004 |
| 4XQ00N04 | FJ591588 | Brazil | 2004 |
| 4XR00N04 | FJ591589 | Brazil | 2004 |
| 4XS00N04 | FJ591591 | Brazil | 2004 |
| 4XT00N04 | FJ591596 | Brazil | 2004 |
| 4XU00N04 | FJ591599 | Brazil | 2004 |
| 4XW00N04 | FJ591601 | Brazil | 2004 |
| 4XX00N04 | FJ591603 | Brazil | 2004 |
| 4XY00N04 | FJ591604 | Brazil | 2004 |
| 4XZ00N04 | FJ591607 | Brazil | 2004 |
| 4YA00N04 | FJ591610 | Brazil | 2004 |
| 4YB00N04 | FJ591611 | Brazil | 2004 |
| 4YC00N04 | FJ591613 | Brazil | 2004 |
| 4YD00N04 | FJ591614 | Brazil | 2004 |
| 4YE00N04 | FJ591615 | Brazil | 2004 |
| 4YF00N04 | FJ591616 | Brazil | 2004 |
| 4YG00N04 | FJ591617 | Brazil | 2004 |
| 4YH00N04 | FJ591618 | Brazil | 2004 |
| 4YI00N04 | FJ591619 | Brazil | 2004 |
| 4YJ00N04 | FJ591620 | Brazil | 2004 |
| 4YK00N04 | FJ591621 | Brazil | 2004 |
| 4YL00N04 | FJ591622 | Brazil | 2004 |
| 4YM00N04 | FJ591623 | Brazil | 2004 |
| 4YN00N04 | FJ591624 | Brazil | 2004 |
| 4YO00N04 | FJ591625 | Brazil | 2004 |
| 4YP00N05 | FJ591627 | Brazil | 2005 |
| 4YQ00N05 | FJ591628 | Brazil | 2005 |
| 4YR00N05 | FJ591629 | Brazil | 2005 |
| 4YS00N05 | FJ591630 | Brazil | 2005 |
| 4YT00N05 | FJ591631 | Brazil | 2005 |
| 4YU00N05 | FJ591632 | Brazil | 2005 |
| 4YV00N05 | FJ591633 | Brazil | 2005 |
| 4YW00N05 | FJ591634 | Brazil | 2005 |
| 4YX00N05 | FJ591635 | Brazil | 2005 |
| 4YY00N05 | FJ591636 | Brazil | 2005 |
| 4YZ00N05 | FJ591637 | Brazil | 2005 |
| 4ZA00N05 | FJ591638 | Brazil | 2005 |
| 4ZB00N05 | FJ591640 | Brazil | 2005 |
| 4ZC00N05 | FJ591641 | Brazil | 2005 |
| 4ZD00N05 | FJ591642 | Brazil | 2005 |
| 4ZE00N05 | FJ591643 | Brazil | 2005 |
| 4ZF00N05 | FJ591645 | Brazil | 2005 |
| 4ZG00N05 | FJ591646 | Brazil | 2005 |
| 4ZH00N05 | FJ591647 | Brazil | 2005 |
| 4ZI00N05 | FJ591649 | Brazil | 2005 |
| 4ZJ00N05 | FJ591650 | Brazil | 2005 |
| 4ZK00N05 | FJ591652 | Brazil | 2005 |
| 4ZL00N05 | FJ591653 | Brazil | 2005 |
| 4ZM00N05 | FJ591654 | Brazil | 2005 |
| 4ZN00N05 | FJ591656 | Brazil | 2005 |
| 4ZO00N05 | FJ591657 | Brazil | 2005 |
| 4ZP00N05 | FJ591659 | Brazil | 2005 |
| 4ZQ00N05 | FJ591660 | Brazil | 2005 |
| 4ZR00N06 | FJ591661 | Brazil | 2006 |
| 4ZS00N06 | FJ591662 | Brazil | 2006 |
| 4ZT00N06 | FJ591663 | Brazil | 2006 |
| 4ZU00N06 | FJ591666 | Brazil | 2006 |
| 4ZW00N06 | FJ591668 | Brazil | 2006 |
| 4ZX00N06 | FJ591669 | Brazil | 2006 |
| 4ZY00N06 | FJ591670 | Brazil | 2006 |
| 4ZZ00N06 | FJ591671 | Brazil | 2006 |
| 40A00N06 | FJ591672 | Brazil | 2006 |
| 40B00N06 | FJ591673 | Brazil | 2006 |
| 40C00N06 | FJ591674 | Brazil | 2006 |
| 40D00N06 | FJ591675 | Brazil | 2006 |
| 40E00N06 | FJ591676 | Brazil | 2006 |
| 40F00N06 | FJ591677 | Brazil | 2006 |
| 40G00N06 | FJ591679 | Brazil | 2006 |
| 40H00N06 | FJ591680 | Brazil | 2006 |
| 40I00N06 | FJ591682 | Brazil | 2006 |
| 40J00N06 | FJ591683 | Brazil | 2006 |
| 40K00N06 | FJ591684 | Brazil | 2006 |
| 40L00N06 | FJ591685 | Brazil | 2006 |
| 40M00N06 | FJ591686 | Brazil | 2006 |
| 40O00N06 | FJ591689 | Brazil | 2006 |
| 40P00N06 | FJ591690 | Brazil | 2006 |
| 40Q00N06 | FJ591691 | Brazil | 2006 |
| 40R00N06 | FJ591692 | Brazil | 2006 |
| 40S00N06 | FJ591694 | Brazil | 2006 |
| 40T00N06 | FJ591695 | Brazil | 2006 |
| 40U00N06 | FJ591696 | Brazil | 2006 |
| 40V00N06 | FJ591697 | Brazil | 2006 |
| 40W00N06 | FJ591698 | Brazil | 2006 |
| 40Y00N06 | FJ591700 | Brazil | 2006 |
| 40Z00N06 | FJ591701 | Brazil | 2006 |
| 41A00N06 | FJ591702 | Brazil | 2006 |
| 41B00N06 | FJ591703 | Brazil | 2006 |
| 41C00N06 | FJ591704 | Brazil | 2006 |
| 41E00N06 | FJ591707 | Brazil | 2006 |
| 41F00N06 | FJ591708 | Brazil | 2006 |
| 41G00N06 | FJ591709 | Brazil | 2006 |
| 41H00N06 | FJ591710 | Brazil | 2006 |
| 41I00N06 | FJ591711 | Brazil | 2006 |
| 41J00N06 | FJ591712 | Brazil | 2006 |
| 41K00N06 | FJ591713 | Brazil | 2006 |
| 41L00N06 | FJ591714 | Brazil | 2006 |
| 41M00N06 | FJ591717 | Brazil | 2006 |
| 41N00N06 | FJ591718 | Brazil | 2006 |
| 41O00N06 | FJ591719 | Brazil | 2006 |
| 41P00N06 | FJ591720 | Brazil | 2006 |
| 41Q00N06 | FJ591721 | Brazil | 2006 |
| 41R00N06 | FJ591722 | Brazil | 2006 |
| 41S00N06 | FJ591723 | Brazil | 2006 |
| 41T00N06 | FJ591724 | Brazil | 2006 |
| 41U00N06 | FJ591725 | Brazil | 2006 |
| 41V00N06 | FJ591727 | Brazil | 2006 |
| 41W00N06 | FJ591729 | Brazil | 2006 |
| 41X00N04 | FJ591730 | Brazil | 2004 |
| 41Y00N06 | FJ591732 | Brazil | 2006 |
| 41Z00N06 | FJ591733 | Brazil | 2006 |
| 42A00N06 | FJ591734 | Brazil | 2006 |
| 42B00N06 | FJ591735 | Brazil | 2006 |
| 42C00N06 | FJ591736 | Brazil | 2006 |
| 42D00N06 | FJ591737 | Brazil | 2006 |
| 42E00N06 | FJ591740 | Brazil | 2006 |
| 42F00N06 | FJ591741 | Brazil | 2006 |
| 42G00N06 | FJ591742 | Brazil | 2006 |
| 42H00N06 | FJ591743 | Brazil | 2006 |
| 42I00N06 | FJ591744 | Brazil | 2006 |
| 42J00N06 | FJ591745 | Brazil | 2006 |
| 42K00N06 | FJ591746 | Brazil | 2006 |
| 42L00N06 | FJ591747 | Brazil | 2006 |
| 42M00N06 | FJ591748 | Brazil | 2006 |
| 42N00N06 | FJ591749 | Brazil | 2006 |
| 42O00N06 | FJ591750 | Brazil | 2006 |
| 42P00N06 | FJ591751 | Brazil | 2006 |
| 42Q00N06 | FJ591752 | Brazil | 2006 |
| 42T00N06 | FJ591755 | Brazil | 2006 |
| 42U00N06 | FJ591757 | Brazil | 2006 |
| 42V00N06 | FJ591758 | Brazil | 2006 |
| 42W00N06 | FJ591759 | Brazil | 2006 |
| 42X00N06 | FJ591760 | Brazil | 2006 |
| 42Y00N06 | FJ591761 | Brazil | 2006 |
| 42Z00N06 | FJ591762 | Brazil | 2006 |
| 43A00N06 | FJ591763 | Brazil | 2006 |
| 43B00N06 | FJ591765 | Brazil | 2006 |
| 43C00N06 | FJ591766 | Brazil | 2006 |
| 43D00N06 | FJ591767 | Brazil | 2006 |
| 43F00N06 | FJ591769 | Brazil | 2006 |
| 43G00N06 | FJ591770 | Brazil | 2006 |
| 43H00N06 | FJ591771 | Brazil | 2006 |
| 43I00N06 | FJ591772 | Brazil | 2006 |
| 43J00N06 | FJ591773 | Brazil | 2006 |
| 43K00N06 | FJ591774 | Brazil | 2006 |
| 43M00N06 | FJ591776 | Brazil | 2006 |
| 43N00N06 | FJ591777 | Brazil | 2006 |
| 43O00N06 | FJ591779 | Brazil | 2006 |
| 43P00N06 | FJ591780 | Brazil | 2006 |
| 43Q00N06 | FJ591781 | Brazil | 2006 |
| 43R00N06 | FJ591782 | Brazil | 2006 |
| 43S00N06 | FJ591783 | Brazil | 2006 |
| 43T00N06 | FJ591785 | Brazil | 2006 |
| 43U00N06 | FJ591786 | Brazil | 2006 |
| 43V00N06 | FJ591787 | Brazil | 2006 |
| 43W00N06 | FJ591788 | Brazil | 2006 |
| 43X00N06 | FJ591789 | Brazil | 2006 |
| 43Y00N06 | FJ591790 | Brazil | 2006 |
| 43Z00N06 | FJ591791 | Brazil | 2006 |
| 44A00N06 | FJ591792 | Brazil | 2006 |
| 44B00N06 | FJ591793 | Brazil | 2006 |
| 44C00N06 | FJ591794 | Brazil | 2006 |
| 44D00N06 | FJ591795 | Brazil | 2006 |
| 44E00N06 | FJ591798 | Brazil | 2006 |
| 44F00N06 | FJ591799 | Brazil | 2006 |
| 44G00N06 | FJ591800 | Brazil | 2006 |
| 44H00N06 | FJ591801 | Brazil | 2006 |
| 44J00N06 | FJ591803 | Brazil | 2006 |
| 44K00N06 | FJ591804 | Brazil | 2006 |
| 44L00N06 | FJ591805 | Brazil | 2006 |
| 44M00N06 | FJ591807 | Brazil | 2006 |
| 44N00N06 | FJ591808 | Brazil | 2006 |
| 44P00N06 | FJ591810 | Brazil | 2006 |
| 44Q00N06 | FJ591811 | Brazil | 2006 |
| 44R00N06 | FJ591812 | Brazil | 2006 |
| 44S00N06 | FJ591814 | Brazil | 2006 |
| 44T00N06 | FJ591815 | Brazil | 2006 |
| 44U00N06 | FJ591816 | Brazil | 2006 |
| 44V00N06 | FJ591817 | Brazil | 2006 |
| 44W00N06 | FJ591818 | Brazil | 2006 |
| 44X00N06 | FJ591819 | Brazil | 2006 |
| 44Y00N06 | FJ591820 | Brazil | 2006 |
| 44Z00N02 | FJ591821 | Brazil | 2002 |
| 45A00N02 | FJ591822 | Brazil | 2002 |
| 45B00N02 | FJ591823 | Brazil | 2002 |
| 45D00N02 | FJ591825 | Brazil | 2002 |
| 45E00N02 | FJ591826 | Brazil | 2002 |
| 45F00N02 | FJ591828 | Brazil | 2002 |
| 45G00N02 | FJ591829 | Brazil | 2002 |
| 45H00N02 | FJ591830 | Brazil | 2002 |
| 45I00N02 | FJ591831 | Brazil | 2002 |
| 45J00N02 | FJ591832 | Brazil | 2002 |
| 45K00N02 | FJ591833 | Brazil | 2002 |
| 45L00N02 | FJ591834 | Brazil | 2002 |
| 45N00N02 | FJ591837 | Brazil | 2002 |
| 45O00N02 | FJ591838 | Brazil | 2002 |
| 45P00N02 | FJ591839 | Brazil | 2002 |
| 45Q00N02 | FJ591840 | Brazil | 2002 |
| 45R00N02 | FJ591841 | Brazil | 2002 |
| 45S00N02 | FJ591842 | Brazil | 2002 |
| 45T00N02 | FJ591843 | Brazil | 2002 |
| 45U00N02 | FJ591844 | Brazil | 2002 |
| 45V00N02 | FJ591845 | Brazil | 2002 |
| 45W00N02 | FJ591846 | Brazil | 2002 |
| 45X00N02 | FJ591847 | Brazil | 2002 |
| 45Y00N02 | FJ591848 | Brazil | 2002 |
| 45Z00N02 | FJ591849 | Brazil | 2002 |
| 46A00N02 | FJ591850 | Brazil | 2002 |
| 46B00N02 | FJ591851 | Brazil | 2002 |
| 46C00N02 | FJ591852 | Brazil | 2002 |
| 46D00N02 | FJ591854 | Brazil | 2002 |
| 46E00N02 | FJ591855 | Brazil | 2002 |
| 46F00N02 | FJ591856 | Brazil | 2002 |
| 46G00N03 | FJ591857 | Brazil | 2003 |
| 46H00N02 | FJ591858 | Brazil | 2002 |
| 46I00N02 | FJ591860 | Brazil | 2002 |
| 46J00N02 | FJ591861 | Brazil | 2002 |
| 46K00N02 | FJ591862 | Brazil | 2002 |
| 46L00N02 | FJ591863 | Brazil | 2002 |
| 46M00N02 | FJ591864 | Brazil | 2002 |
| 46N00N02 | FJ591865 | Brazil | 2002 |
| 46P00N03 | FJ591867 | Brazil | 2003 |
| 46Q00N01 | FJ591868 | Brazil | 2001 |
| 46R00N03 | FJ591869 | Brazil | 2003 |
| 46S00N03 | FJ591870 | Brazil | 2003 |
| 46T00N03 | FJ591871 | Brazil | 2003 |
| 46U00N02 | FJ591872 | Brazil | 2002 |
| 46V00N02 | FJ591873 | Brazil | 2002 |
| 46X00N02 | FJ591875 | Brazil | 2002 |
| 46Z00N02 | FJ591877 | Brazil | 2002 |
| 47A00N03 | FJ591878 | Brazil | 2003 |
| 47B00N03 | FJ591879 | Brazil | 2003 |
| 47C00N03 | FJ591880 | Brazil | 2003 |
| 47D00N02 | FJ591881 | Brazil | 2002 |
| 47G00N02 | FJ591884 | Brazil | 2002 |
| 47H00N02 | FJ591885 | Brazil | 2002 |
| 47I00N02 | FJ591886 | Brazil | 2002 |
| 47K00N02 | FJ591888 | Brazil | 2002 |
| 47L00N02 | FJ591889 | Brazil | 2002 |
| 47N00N02 | FJ591891 | Brazil | 2002 |
| 47O00N03 | FJ591892 | Brazil | 2003 |
| 47P00N03 | FJ591893 | Brazil | 2003 |
| 47Q00N03 | FJ591894 | Brazil | 2003 |
| 47R00N03 | FJ591895 | Brazil | 2003 |
| 47S00N03 | FJ591896 | Brazil | 2003 |
| 47T00N02 | FJ591897 | Brazil | 2002 |
| 47U00N03 | FJ591898 | Brazil | 2003 |
| 47V00N03 | FJ591900 | Brazil | 2003 |
| 47W00N03 | FJ591902 | Brazil | 2003 |
| 47X00N03 | FJ591903 | Brazil | 2003 |
| 47Y00N02 | FJ591904 | Brazil | 2002 |
| 48A00N03 | FJ591907 | Brazil | 2003 |
| 48B00N03 | FJ591909 | Brazil | 2003 |
| 48C00N03 | FJ591911 | Brazil | 2003 |
| 48D00N03 | FJ591912 | Brazil | 2003 |
| 48E00N03 | FJ591913 | Brazil | 2003 |
| 48G00N03 | FJ591915 | Brazil | 2003 |
| 48H00N03 | FJ591916 | Brazil | 2003 |
| 48I00N03 | FJ591917 | Brazil | 2003 |
| 48J00N03 | FJ591919 | Brazil | 2003 |
| 48L00N03 | FJ591923 | Brazil | 2003 |
| 48M00N03 | FJ591924 | Brazil | 2003 |
| 48N00N02 | FJ591925 | Brazil | 2002 |
| 48O00N03 | FJ591926 | Brazil | 2003 |
| 48P00N03 | FJ591927 | Brazil | 2003 |
| 48Q00N03 | FJ591928 | Brazil | 2003 |
| 48R00N02 | FJ591929 | Brazil | 2002 |
| 48S00N03 | FJ591931 | Brazil | 2003 |
| 48T00N03 | FJ591932 | Brazil | 2003 |
| 48U00N03 | FJ591933 | Brazil | 2003 |
| 48V00N03 | FJ591935 | Brazil | 2003 |
| 48W00N03 | FJ591936 | Brazil | 2003 |
| 48X00N03 | FJ591937 | Brazil | 2003 |
| 48Y00N03 | FJ591938 | Brazil | 2003 |
| 48Z00N03 | FJ591942 | Brazil | 2003 |
| 49A00N03 | FJ591943 | Brazil | 2003 |
| 49B00N03 | FJ591944 | Brazil | 2003 |
| 49D00N02 | FJ591946 | Brazil | 2002 |
| 49E00N03 | FJ591947 | Brazil | 2003 |
| 49F00N03 | FJ591949 | Brazil | 2003 |
| 49G00N03 | FJ591950 | Brazil | 2003 |
| 49H00N03 | FJ591951 | Brazil | 2003 |
| 49I00N03 | FJ591952 | Brazil | 2003 |
| 49J00N02 | FJ591956 | Brazil | 2002 |
| 49K00N03 | FJ591960 | Brazil | 2003 |
| 49L00N03 | FJ591961 | Brazil | 2003 |
| 49M00N03 | FJ591962 | Brazil | 2003 |
| 49N00N03 | FJ591963 | Brazil | 2003 |
| 49P00N03 | FJ591965 | Brazil | 2003 |
| 49Q00N03 | FJ591966 | Brazil | 2003 |
| 49R00N03 | FJ591969 | Brazil | 2003 |
| 49S00N03 | FJ591970 | Brazil | 2003 |
| 49T00N03 | FJ591971 | Brazil | 2003 |
| 49U00N03 | FJ591972 | Brazil | 2003 |
| 49V00N03 | FJ591974 | Brazil | 2003 |
| 49W00N03 | FJ591977 | Brazil | 2003 |
| 49X00N03 | FJ591978 | Brazil | 2003 |
| 49Y00N03 | FJ591979 | Brazil | 2003 |
| 49Z00N03 | FJ591982 | Brazil | 2003 |
| 5AA00N03 | FJ591983 | Brazil | 2003 |
| 5AB00N02 | FJ591984 | Brazil | 2002 |
| 5AC00N03 | FJ591985 | Brazil | 2003 |
| 5AD00N03 | FJ591986 | Brazil | 2003 |
| 5AF00N03 | FJ591988 | Brazil | 2003 |
| 5AG00N03 | FJ591989 | Brazil | 2003 |
| 5AH00N03 | FJ591990 | Brazil | 2003 |
| 5AI00N03 | FJ591991 | Brazil | 2003 |
| 5AJ00N03 | FJ591992 | Brazil | 2003 |
| 5AK00N03 | FJ591993 | Brazil | 2003 |
| 5AL00N02 | FJ591996 | Brazil | 2002 |
| 5AM00N03 | FJ591997 | Brazil | 2003 |
| 5AN00N03 | FJ591999 | Brazil | 2003 |
| 5AQ00N03 | FJ592002 | Brazil | 2003 |
| 5AR00N03 | FJ592004 | Brazil | 2003 |
| 5AS00N03 | FJ592005 | Brazil | 2003 |
| 5AT00N03 | FJ592006 | Brazil | 2003 |
| 5AU00N03 | FJ592007 | Brazil | 2003 |
| 5AV00N03 | FJ592008 | Brazil | 2003 |
| 5AW00N03 | FJ592009 | Brazil | 2003 |
| 5AX00N03 | FJ592010 | Brazil | 2003 |
| 5AY00N03 | FJ592011 | Brazil | 2003 |
| 5AZ00N03 | FJ592012 | Brazil | 2003 |
| 5BA00N03 | FJ592014 | Brazil | 2003 |
| 5BB00N03 | FJ592015 | Brazil | 2003 |
| 5BC00N03 | FJ592016 | Brazil | 2003 |
| 5BD00N03 | FJ592017 | Brazil | 2003 |
| 5BE00N03 | FJ592019 | Brazil | 2003 |
| 5BF00N03 | FJ592020 | Brazil | 2003 |
| 5BG00N02 | FJ592021 | Brazil | 2002 |
| 5BH00N02 | FJ592022 | Brazil | 2002 |
| 5BJ00N02 | FJ592024 | Brazil | 2002 |
| 5BK00N02 | FJ592025 | Brazil | 2002 |
| 5BL00N02 | FJ592026 | Brazil | 2002 |
| 5BM00N02 | FJ592027 | Brazil | 2002 |
| 5BN00N02 | FJ592028 | Brazil | 2002 |
| 5BO00N02 | FJ592029 | Brazil | 2002 |
| 5BP00N02 | FJ592030 | Brazil | 2002 |
| 5BQ00N02 | FJ592031 | Brazil | 2002 |
| 5BR00N02 | FJ592032 | Brazil | 2002 |
| 5BS00N02 | FJ592033 | Brazil | 2002 |
| 5BT00N02 | FJ592034 | Brazil | 2002 |
| 5BU00N02 | FJ592035 | Brazil | 2002 |
| 5BV00N02 | FJ592036 | Brazil | 2002 |
| 5BW00N02 | FJ592037 | Brazil | 2002 |
| 5BX00N02 | FJ592038 | Brazil | 2002 |
| 5BY00N02 | FJ592039 | Brazil | 2002 |
| 5BZ00N02 | FJ592041 | Brazil | 2002 |
| 5CA00N02 | FJ592042 | Brazil | 2002 |
| 5CB00N02 | FJ592043 | Brazil | 2002 |
| 5CC00N02 | FJ592044 | Brazil | 2002 |
| 5CD00N02 | FJ592045 | Brazil | 2002 |
| 5CE00N02 | FJ592046 | Brazil | 2002 |
| 5CF00N02 | FJ592047 | Brazil | 2002 |
| 5CG00N02 | FJ592048 | Brazil | 2002 |
| 5CH00N02 | FJ592049 | Brazil | 2002 |
| 5CI00N02 | FJ592050 | Brazil | 2002 |
| 5CK00N02 | FJ592052 | Brazil | 2002 |
| 5CL00N02 | FJ592053 | Brazil | 2002 |
| 5CM00N02 | FJ592054 | Brazil | 2002 |
| 5CN00N02 | FJ592055 | Brazil | 2002 |
| 5CO00N02 | FJ592056 | Brazil | 2002 |
| 5CP00N02 | FJ592057 | Brazil | 2002 |
| 5CQ00N02 | FJ592058 | Brazil | 2002 |
| 5CR00N02 | FJ592059 | Brazil | 2002 |
| 5CS00N02 | FJ592060 | Brazil | 2002 |
| 5CT00N02 | FJ592061 | Brazil | 2002 |
| 5CU00N02 | FJ592062 | Brazil | 2002 |
| 5CW00N02 | FJ592064 | Brazil | 2002 |
| 5CX00N02 | FJ592065 | Brazil | 2002 |
| 5CY00N02 | FJ592066 | Brazil | 2002 |
| 5CZ00N02 | FJ592067 | Brazil | 2002 |
| 5DA00N02 | FJ592068 | Brazil | 2002 |
| 5DB00N02 | FJ592069 | Brazil | 2002 |
| 5DC00N02 | FJ592070 | Brazil | 2002 |
| 5DD00N02 | FJ592071 | Brazil | 2002 |
| 5DE00N02 | FJ592072 | Brazil | 2002 |
| 5DF00N03 | FJ592073 | Brazil | 2003 |
| 5DGNND02 | FJ966984 | Brazil | 2002 |
| 5DINND02 | FJ966986 | Brazil | 2002 |
| 5DJNND02 | FJ966987 | Brazil | 2002 |
| 5DKNND02 | FJ966989 | Brazil | 2002 |
| 5DLNND02 | FJ966990 | Brazil | 2002 |
| 5DMNND02 | FJ966991 | Brazil | 2002 |
| 5DNNND02 | FJ966992 | Brazil | 2002 |
| 5DONND02 | FJ966993 | Brazil | 2002 |
| 5DPNND02 | FJ966994 | Brazil | 2002 |
| 5DQNND02 | FJ966995 | Brazil | 2002 |
| 5DRNND02 | FJ966996 | Brazil | 2002 |
| 5DSNND02 | FJ966997 | Brazil | 2002 |
| 5DTNND02 | FJ966998 | Brazil | 2002 |
| 5DUNND02 | FJ966999 | Brazil | 2002 |
| 5DVNND02 | FJ967000 | Brazil | 2002 |
| 5DWNND02 | FJ967001 | Brazil | 2002 |
| 5DXNND02 | FJ967002 | Brazil | 2002 |
| 5DYNND02 | FJ967003 | Brazil | 2002 |
| 5DZNND02 | FJ967004 | Brazil | 2002 |
| 5EANND02 | FJ967005 | Brazil | 2002 |
| 5EBNND02 | FJ967006 | Brazil | 2002 |
| 5ECNND02 | FJ967007 | Brazil | 2002 |
| 5EDNND02 | FJ967008 | Brazil | 2002 |
| 5EENND02 | FJ967009 | Brazil | 2002 |
| 5EFNND02 | FJ967010 | Brazil | 2002 |
| 5EGNND02 | FJ967011 | Brazil | 2002 |
| 5EHNND02 | FJ967012 | Brazil | 2002 |
| 5EINND02 | FJ967014 | Brazil | 2002 |
| 5EJNND02 | FJ967015 | Brazil | 2002 |
| 5EKNND02 | FJ967016 | Brazil | 2002 |
| 5ELNND02 | FJ967017 | Brazil | 2002 |
| 5ENNND03 | FJ967020 | Brazil | 2003 |
| 5EONND03 | FJ967021 | Brazil | 2003 |
| 5EPNND03 | FJ967022 | Brazil | 2003 |
| 5EQNND03 | FJ967023 | Brazil | 2003 |
| 5ERNND03 | FJ967024 | Brazil | 2003 |
| 5ESNND03 | FJ967025 | Brazil | 2003 |
| 5ETNND03 | FJ967026 | Brazil | 2003 |
| 5EVNND03 | FJ967028 | Brazil | 2003 |
| 5EWNND03 | FJ967029 | Brazil | 2003 |
| 5EXNND03 | FJ967030 | Brazil | 2003 |
| 5EYNND03 | FJ967031 | Brazil | 2003 |
| 5FANND03 | FJ967034 | Brazil | 2003 |
| 5FCNND03 | FJ967038 | Brazil | 2003 |
| 5FDNND03 | FJ967039 | Brazil | 2003 |
| 5FENND03 | FJ967040 | Brazil | 2003 |
| 5FFNND03 | FJ967041 | Brazil | 2003 |
| 5FGNND03 | FJ967042 | Brazil | 2003 |
| 5FHNND03 | FJ967043 | Brazil | 2003 |
| 5FJNND03 | FJ967045 | Brazil | 2003 |
| 5FKNND03 | FJ967046 | Brazil | 2003 |
| 5FLNND03 | FJ967047 | Brazil | 2003 |
| 5FNNND03 | FJ967050 | Brazil | 2003 |
| 5FONND03 | FJ967051 | Brazil | 2003 |
| 5FPNND03 | FJ967053 | Brazil | 2003 |
| 5FQNND03 | FJ967054 | Brazil | 2003 |
| 5FRNND03 | FJ967055 | Brazil | 2003 |
| 5FSNND03 | FJ967056 | Brazil | 2003 |
| 5FTNND03 | FJ967057 | Brazil | 2003 |
| 5FUNND03 | FJ967058 | Brazil | 2003 |
| 5FVNND03 | FJ967059 | Brazil | 2003 |
| 5FWNND03 | FJ967060 | Brazil | 2003 |
| 5FXNND03 | FJ967061 | Brazil | 2003 |
| 5FYNND03 | FJ967063 | Brazil | 2003 |
| 5FZNND03 | FJ967064 | Brazil | 2003 |
| 5GANND03 | FJ967067 | Brazil | 2003 |
| 5GBNND03 | FJ967068 | Brazil | 2003 |
| 5GCNND03 | FJ967069 | Brazil | 2003 |
| 5GDNND03 | FJ967070 | Brazil | 2003 |
| 5GFNND03 | FJ967072 | Brazil | 2003 |
| 5GGNND03 | FJ967073 | Brazil | 2003 |
| 5GINND04 | FJ967075 | Brazil | 2004 |
| 5GJNND04 | FJ967076 | Brazil | 2004 |
| 5GKNND04 | FJ967077 | Brazil | 2004 |
| 5GLNND04 | FJ967078 | Brazil | 2004 |
| 5GMNND04 | FJ967079 | Brazil | 2004 |
| 5GONND04 | FJ967082 | Brazil | 2004 |
| 5GPNND04 | FJ967083 | Brazil | 2004 |
| 5GSNND04 | FJ967086 | Brazil | 2004 |
| 5GTNND04 | FJ967087 | Brazil | 2004 |
| 5GUNND04 | FJ967088 | Brazil | 2004 |
| 5GVNND04 | FJ967089 | Brazil | 2004 |
| 5GWNND04 | FJ967090 | Brazil | 2004 |
| 5GXNND04 | FJ967091 | Brazil | 2004 |
| 5GYNND04 | FJ967092 | Brazil | 2004 |
| 5HBNND04 | FJ967095 | Brazil | 2004 |
| 5HCNND04 | FJ967096 | Brazil | 2004 |
| 5HDNND04 | FJ967097 | Brazil | 2004 |
| 5HENND04 | FJ967098 | Brazil | 2004 |
| 5HHNND04 | FJ967101 | Brazil | 2004 |
| 5HJNND04 | FJ967104 | Brazil | 2004 |
| 5HKNND04 | FJ967106 | Brazil | 2004 |
| 5HMNND04 | FJ967109 | Brazil | 2004 |
| 5HNNND04 | FJ967110 | Brazil | 2004 |
| 5HONND05 | FJ967112 | Brazil | 2005 |
| 5HPNND05 | FJ967113 | Brazil | 2005 |
| 5HQNND05 | FJ967114 | Brazil | 2005 |
| 5HRNND05 | FJ967115 | Brazil | 2005 |
| 5HSNND05 | FJ967116 | Brazil | 2005 |
| 5HTNND06 | GQ294596 | Brazil | 2006 |
| 5HVNND06 | GQ294598 | Brazil | 2006 |
| 5HWNND06 | GQ294599 | Brazil | 2006 |
| 5HYNND06 | GQ294602 | Brazil | 2006 |
| 5IANND06 | GQ294604 | Brazil | 2006 |
| 5IBNND06 | GQ294605 | Brazil | 2006 |
| 5IENND06 | GQ294610 | Brazil | 2006 |
| 5IFNND06 | GQ294612 | Brazil | 2006 |
| 5IGNND06 | GQ294614 | Brazil | 2006 |
| 5IKNND06 | GQ294619 | Brazil | 2006 |
| 5ILNND06 | GQ294620 | Brazil | 2006 |
| 5IONND06 | GQ294624 | Brazil | 2006 |
| 5IPNND06 | GQ294626 | Brazil | 2006 |
| 5JQNND04 | GU288709 | Brazil | 2004 |
| 5JR0ND05 | GU288710 | Brazil | 2005 |
| 5JS0ND05 | GU288712 | Brazil | 2005 |
| 5JT00D06 | GU288714 | Brazil | 2006 |
| 5JU12D04 | GU288715 | Brazil | 2004 |
| 5JV00D06 | GU288716 | Brazil | 2006 |
| 5JW00D05 | GU288717 | Brazil | 2005 |
| 5JX00D04 | GU288718 | Brazil | 2004 |
| 5JY00D06 | GU288719 | Brazil | 2006 |
| 5JZ00D06 | GU288720 | Brazil | 2006 |
| 5KB00D06 | GU288722 | Brazil | 2006 |
| 5KC00D04 | GU288723 | Brazil | 2004 |
| 5KD00D06 | GU288724 | Brazil | 2006 |
| 5KE00D06 | GU288725 | Brazil | 2006 |
| 5KF12D03 | GU288726 | Brazil | 2003 |
| 5KG00D04 | GU288727 | Brazil | 2004 |
| 5KH00D06 | GU288729 | Brazil | 2006 |
| 5KJ00D05 | GU288732 | Brazil | 2005 |
| 5KK12D04 | GU288733 | Brazil | 2004 |
| 5KL00D04 | GU288734 | Brazil | 2004 |
| 5KM00D06 | GU288735 | Brazil | 2006 |
| 5KP00D05 | GU288738 | Brazil | 2005 |
| 5KR00D05 | GU288741 | Brazil | 2005 |
| 5KS00D05 | GU288742 | Brazil | 2005 |
| 5KU12D03 | GU288744 | Brazil | 2003 |
| 5KV22D04 | GU288745 | Brazil | 2004 |
| 5KW00D05 | GU288746 | Brazil | 2005 |
| 5KX00D05 | GU288747 | Brazil | 2005 |
| 5KY00D05 | GU288748 | Brazil | 2005 |
| 5KZ00D06 | GU288749 | Brazil | 2006 |
| 5LA00D04 | GU288750 | Brazil | 2004 |
| 5LB00D06 | GU288751 | Brazil | 2006 |
| 5LC00D06 | GU288752 | Brazil | 2006 |
| 5LG00D05 | GU288757 | Brazil | 2005 |
| 5LH20D04 | GU288758 | Brazil | 2004 |
| 5LI00D04 | GU288759 | Brazil | 2004 |
| 5LJ12D03 | GU288760 | Brazil | 2003 |
| 5LL22D04 | GU288764 | Brazil | 2004 |
| 5LM00D05 | GU288765 | Brazil | 2005 |
| 5LN00D05 | GU288766 | Brazil | 2005 |
| 5LO00D06 | GU288767 | Brazil | 2006 |
| 5LQ00D06 | GU288770 | Brazil | 2006 |
| 5LR00D05 | GU288771 | Brazil | 2005 |
| 5LS00D05 | GU288773 | Brazil | 2005 |
| 5LT00D05 | GU288774 | Brazil | 2005 |
| 5LU00D06 | GU288775 | Brazil | 2006 |
| 5LV00D06 | GU288776 | Brazil | 2006 |
| 5LW00D04 | GU288778 | Brazil | 2004 |
| 5LX00D04 | GU288779 | Brazil | 2004 |
| 5LZ00D05 | GU288781 | Brazil | 2005 |
| 5MA00D06 | GU288782 | Brazil | 2006 |
| 5MB00D05 | GU288783 | Brazil | 2005 |
| 5MC12D04 | GU288784 | Brazil | 2004 |
| 5MD00D05 | GU288785 | Brazil | 2005 |
| 5ME12D04 | GU288786 | Brazil | 2004 |
| 5MF00D06 | GU288787 | Brazil | 2006 |
| 5MG00D06 | GU288788 | Brazil | 2006 |
| 5MH12D03 | GU288789 | Brazil | 2003 |
| 5MJ00D05 | GU288791 | Brazil | 2005 |
| 5MK00D05 | GU288792 | Brazil | 2005 |
| 5ML00D05 | GU288793 | Brazil | 2005 |
| 5MM00D06 | GU288794 | Brazil | 2006 |
| 5MN00D04 | GU288795 | Brazil | 2004 |
| 5MP00D05 | GU288797 | Brazil | 2005 |
| 5MQ00D05 | GU288798 | Brazil | 2005 |
| 5MR00D04 | GU288799 | Brazil | 2004 |
| 5MS12D04 | GU288800 | Brazil | 2004 |
| 5MT00D05 | GU288802 | Brazil | 2005 |
| 5MU00D06 | GU288803 | Brazil | 2006 |
| 5MV00D04 | GU288804 | Brazil | 2004 |
| 5MX00D06 | GU288806 | Brazil | 2006 |
| 5MY00D05 | GU288807 | Brazil | 2005 |
| 5MZ00D06 | GU288808 | Brazil | 2006 |
| 5NA00D04 | GU288810 | Brazil | 2004 |
| 5NB00D05 | GU288811 | Brazil | 2005 |
| 5NC13D03 | GU288813 | Brazil | 2003 |
| 5ND00D05 | HM024743 | Brazil | 2005 |
| 5NF00D05 | HM024745 | Brazil | 2005 |
| 5NG00D05 | HM024747 | Brazil | 2005 |
| 5NH00D05 | HM024748 | Brazil | 2005 |
| 5NI00D05 | HM024750 | Brazil | 2005 |
| 5NJ00D05 | HM024751 | Brazil | 2005 |
| 5NK00D05 | HM024752 | Brazil | 2005 |
| 5NL00D05 | HM024753 | Brazil | 2005 |
| 5NM00D05 | HM024754 | Brazil | 2005 |
| 5NN00D05 | HM024755 | Brazil | 2005 |
| 5NO00D05 | HM024756 | Brazil | 2005 |
| 5NP00D05 | HM024757 | Brazil | 2005 |
| 5NQ00D05 | HM024758 | Brazil | 2005 |
| 5NR00D05 | HM024759 | Brazil | 2005 |
| 5NS00D05 | HM024760 | Brazil | 2005 |
| 5NT00D05 | HM024761 | Brazil | 2005 |
| 5NU00D05 | HM024762 | Brazil | 2005 |
| 5NV00D05 | HM024763 | Brazil | 2005 |
| 5NW00D05 | HM024764 | Brazil | 2005 |
| 5NX00D05 | HM024765 | Brazil | 2005 |
| 5NY00D05 | HM024766 | Brazil | 2005 |
| 5NZ00D05 | HM024767 | Brazil | 2005 |
| 5OA00D05 | HM024768 | Brazil | 2005 |
| 5OB00D05 | HM024769 | Brazil | 2005 |
| 5OC00D05 | HM024770 | Brazil | 2005 |
| 5OD00D05 | HM024771 | Brazil | 2005 |
| 5OE00D05 | HM024772 | Brazil | 2005 |
| 5OF00D05 | HM024773 | Brazil | 2005 |
| 5OG00D05 | HM024776 | Brazil | 2005 |
| 5OH00D05 | HM024778 | Brazil | 2005 |
| 5OI00D05 | hM024781 | Brazil | 2005 |
| 5OJ00D05 | hM024782 | Brazil | 2005 |
| 5OK00D05 | HM024784 | Brazil | 2005 |
| 5OL00D05 | HM024785 | Brazil | 2005 |
| 5OM00D05 | HM024786 | Brazil | 2005 |
| 5ON00D05 | HM024787 | Brazil | 2005 |
| 5OO00D05 | HM024788 | Brazil | 2005 |
| 5OP00D05 | HM024789 | Brazil | 2005 |
| 5OQ00D05 | HM024790 | Brazil | 2005 |
| 5OR00D05 | HM024791 | Brazil | 2005 |
| 5OS00D05 | HM024792 | Brazil | 2005 |
| 5OT00D05 | HM024793 | Brazil | 2005 |
| 5OU00D05 | HM024794 | Brazil | 2005 |
| 5OV00D05 | HM024795 | Brazil | 2005 |
| 5OW00D05 | HM024796 | Brazil | 2005 |
| 5OX00D05 | HM024797 | Brazil | 2005 |
| 5OY00D05 | HM024798 | Brazil | 2005 |
| 5OZ00D05 | HM024799 | Brazil | 2005 |
| 5PA00D05 | HM024800 | Brazil | 2005 |
| 5PC00D05 | HM024802 | Brazil | 2005 |
| 5PD00D05 | HM024803 | Brazil | 2005 |
| 5PE00D05 | HM024804 | Brazil | 2005 |
| 5PF00D05 | HM024807 | Brazil | 2005 |
| 5PG00D05 | HM024808 | Brazil | 2005 |
| 5PH00D05 | HM024809 | Brazil | 2005 |
| 5PI00D05 | HM024812 | Brazil | 2005 |
| 5PJ00D05 | HM024813 | Brazil | 2005 |
| 5PK00D05 | HM024814 | Brazil | 2005 |
| 5PL00D05 | HM024815 | Brazil | 2005 |
| 5PM00D05 | HM024817 | Brazil | 2005 |
| 5PN00D05 | HM024819 | Brazil | 2005 |
| 5PO00D05 | HM024820 | Brazil | 2005 |
| 5PP00D05 | HM024821 | Brazil | 2005 |
| 5PQ00D05 | HM024822 | Brazil | 2005 |
| 5PR00D05 | HM024823 | Brazil | 2005 |
| 5PS00D05 | HM024824 | Brazil | 2005 |
| 5PU00D05 | HM024827 | Brazil | 2005 |
| 5PV00D05 | HM024828 | Brazil | 2005 |
| 5PW00D05 | HM024829 | Brazil | 2005 |
| 5PX00D05 | HM024830 | Brazil | 2005 |
| 5PY00D05 | HM024831 | Brazil | 2005 |
| 5PZ00D05 | HM024832 | Brazil | 2005 |
| 5QA00D05 | HM024833 | Brazil | 2005 |
| 5QB00D05 | HM024834 | Brazil | 2005 |
| 5QC00D05 | HM024835 | Brazil | 2005 |
| 5QD00D05 | HM024836 | Brazil | 2005 |
| 5QE00D05 | HM024837 | Brazil | 2005 |
| 5QF00D05 | HM024839 | Brazil | 2005 |
| 5QG00D05 | HM024840 | Brazil | 2005 |
| 5QH00D05 | HM024841 | Brazil | 2005 |
| 5QI00D05 | HM024842 | Brazil | 2005 |
| 5QJ00D05 | HM024843 | Brazil | 2005 |
| 5QK00D05 | HM024844 | Brazil | 2005 |
| 5QL00D05 | HM024845 | Brazil | 2005 |
| 5QM00D05 | HM024846 | Brazil | 2005 |
| 5QN00D05 | HM024847 | Brazil | 2005 |
| 5QO00D05 | HM024848 | Brazil | 2005 |
| 5QP00D05 | HM024849 | Brazil | 2005 |
| 5QQ00D05 | HM024851 | Brazil | 2005 |
| 5QR00D05 | HM024853 | Brazil | 2005 |
| 5QS00D05 | HM024854 | Brazil | 2005 |
| 5QU00D05 | HM024856 | Brazil | 2005 |
| 5QV00D05 | HM024857 | Brazil | 2005 |
| 5QW00D05 | HM024858 | Brazil | 2005 |
| 5QX00D05 | HM024859 | Brazil | 2005 |
| 5QZ00D05 | HM024861 | Brazil | 2005 |
| 5RA00D05 | HM024862 | Brazil | 2005 |
| 5RC00D05 | HM024865 | Brazil | 2005 |
| 5RD00D05 | HM024866 | Brazil | 2005 |
| 5RE00D05 | HM024867 | Brazil | 2005 |
| 5RF00D05 | HM024869 | Brazil | 2005 |
| 5RG00D05 | HM024870 | Brazil | 2005 |
| 5RH00D05 | HM024871 | Brazil | 2005 |
| 5RI00D05 | HM024872 | Brazil | 2005 |
| 5RJ00D05 | HM024873 | Brazil | 2005 |
| 5RK00D05 | HM024875 | Brazil | 2005 |
| 5RL00D05 | HM024876 | Brazil | 2005 |
| 5RM00D05 | HM024877 | Brazil | 2005 |
| 5RO00D05 | HM024879 | Brazil | 2005 |
| 5RP00D05 | HM024882 | Brazil | 2005 |
| 5RQ00D05 | HM024883 | Brazil | 2005 |
| 5RR00D05 | HM024884 | Brazil | 2005 |
| 5RS00D05 | HM024885 | Brazil | 2005 |
| 5RU00D05 | HM024887 | Brazil | 2005 |
| 5RV00D05 | HM024888 | Brazil | 2005 |
| 5RW00D05 | HM024889 | Brazil | 2005 |
| 5RX00D05 | HM024890 | Brazil | 2005 |
| 5RY00D05 | HM024891 | Brazil | 2005 |
| 5RZ00D05 | HM024893 | Brazil | 2005 |
| 5SA00D05 | HM024894 | Brazil | 2005 |
| 5SB00D05 | HM024895 | Brazil | 2005 |
| 5SD00D05 | HM024897 | Brazil | 2005 |
| 5SE00D05 | HM024898 | Brazil | 2005 |
| 5SF00D05 | HM024899 | Brazil | 2005 |
| 5SG00D05 | HM024900 | Brazil | 2005 |
| 5SH00D05 | HM024902 | Brazil | 2005 |
| 5SI00D05 | HM024903 | Brazil | 2005 |
| 5SJ00D05 | HM024904 | Brazil | 2005 |
| 5SK00D05 | HM024905 | Brazil | 2005 |
| 5SL00D05 | HM024906 | Brazil | 2005 |
| 5SM00D05 | HM024907 | Brazil | 2005 |
| 5SN00D05 | HM024908 | Brazil | 2005 |
| 5SO00D05 | HM024909 | Brazil | 2005 |
| 5SP00D05 | HM024910 | Brazil | 2005 |
| 5SQ00D05 | HM024911 | Brazil | 2005 |
| 5SR00D05 | HM024912 | Brazil | 2005 |
| 5SS00D05 | HM024913 | Brazil | 2005 |
| 5ST00D05 | HM024914 | Brazil | 2005 |
| 5SU00D05 | HM024915 | Brazil | 2005 |
| 5SV00D05 | HM024916 | Brazil | 2005 |
| 5SW00D05 | HM024917 | Brazil | 2005 |
| 5SY00D05 | HM024919 | Brazil | 2005 |
| 5TB00D05 | HM024922 | Brazil | 2005 |
| 5TC00D05 | HM024923 | Brazil | 2005 |
| 5TD00D05 | HM024924 | Brazil | 2005 |
| 5TF00D05 | HM024927 | Brazil | 2005 |
| 5TG00D05 | HM024929 | Brazil | 2005 |
| 5TH00D05 | HM024930 | Brazil | 2005 |
| 5TK00D05 | HM024934 | Brazil | 2005 |
| 5TL00D05 | HM024936 | Brazil | 2005 |
| 5TM00D05 | HM024937 | Brazil | 2005 |
| 5TN00D05 | HM024939 | Brazil | 2005 |
| 5TO00D05 | HM024941 | Brazil | 2005 |
| 5TP00D05 | HM024942 | Brazil | 2005 |
| 5TQ00D05 | HM024943 | Brazil | 2005 |
| 5TR00D05 | HM024944 | Brazil | 2005 |
| 5TS00D05 | HM024945 | Brazil | 2005 |
| 5TT00D05 | HM024946 | Brazil | 2005 |
| 5TU00D05 | HM024948 | Brazil | 2005 |
| 5TV00D05 | HM024949 | Brazil | 2005 |
| 5TW00D05 | HM024950 | Brazil | 2005 |
| 5TX00D05 | HM024951 | Brazil | 2005 |
| 5TY00D05 | HM024952 | Brazil | 2005 |
| 5TZ00D05 | HM024953 | Brazil | 2005 |
| 5UA00D05 | HM024955 | Brazil | 2005 |
| 5UB00D05 | HM024956 | Brazil | 2005 |
| 5UC00D05 | HM024957 | Brazil | 2005 |
| 5UD00D05 | HM024958 | Brazil | 2005 |
| 5UE00D05 | HM024959 | Brazil | 2005 |
| 5UF00D05 | HM024960 | Brazil | 2005 |
| 5UG00D05 | HM024961 | Brazil | 2005 |
| 5UH00D05 | HM024962 | Brazil | 2005 |
| 5UI00D05 | HM024963 | Brazil | 2005 |
| 5UJ00D05 | HM024964 | Brazil | 2005 |
| 5UK00D05 | HM024965 | Brazil | 2005 |
| 5UL00D05 | HM024967 | Brazil | 2005 |
| 5UM00D05 | HM024968 | Brazil | 2005 |
| 5UN00D05 | HM024969 | Brazil | 2005 |
| 5UO00D05 | HM024970 | Brazil | 2005 |
| 5UP00D05 | HM024971 | Brazil | 2005 |
| 5UQ00D05 | HM024972 | Brazil | 2005 |
| 5UR00D05 | HM024973 | Brazil | 2005 |
| 5US00D05 | HM024974 | Brazil | 2005 |
| 5UT00D05 | HM024975 | Brazil | 2005 |
| 5UU00D05 | HM024976 | Brazil | 2005 |
| 5UV00D05 | HM024977 | Brazil | 2005 |
| 5UW00D05 | HM024978 | Brazil | 2005 |
| 5UX00D05 | HM024979 | Brazil | 2005 |
| 5UY00D05 | HM024982 | Brazil | 2005 |
| 5UZ00D05 | HM024983 | Brazil | 2005 |
| 5WB00D05 | HM024985 | Brazil | 2005 |
| 5WC00D05 | HM024986 | Brazil | 2005 |
| 5WD00D05 | HM024987 | Brazil | 2005 |
| 5WE00D05 | HM024988 | Brazil | 2005 |
| 5WF00D05 | HM024989 | Brazil | 2005 |
| 5WG00D05 | HM024990 | Brazil | 2005 |
| 5WH00D05 | HM024991 | Brazil | 2005 |
| 5WI00D05 | HM024992 | Brazil | 2005 |
| 5WJ00D05 | HM024993 | Brazil | 2005 |
| 5WK00D05 | HM024994 | Brazil | 2005 |
| 5WL00D05 | HM024997 | Brazil | 2005 |
| 5WM00D05 | HM024998 | Brazil | 2005 |
| 5WN00D05 | HM024999 | Brazil | 2005 |
| 5WO00D05 | HM025000 | Brazil | 2005 |
| 5WP00D05 | HM025001 | Brazil | 2005 |
| 5WQ00D05 | HM025002 | Brazil | 2005 |
| 5WR00D05 | HM025005 | Brazil | 2005 |
| 5WS00D05 | HM025006 | Brazil | 2005 |
| 5WT00D05 | HM025007 | Brazil | 2005 |
| 5WU00D05 | HM025008 | Brazil | 2005 |
| 5WV00D05 | HM025009 | Brazil | 2005 |
| 5WX00D05 | HM025012 | Brazil | 2005 |
| 5WY00D05 | HM025013 | Brazil | 2005 |
| 5WZ00D05 | HM025014 | Brazil | 2005 |
| 5XA00D05 | HM025015 | Brazil | 2005 |
| 5XB00D05 | HM025016 | Brazil | 2005 |
| 5XC00D05 | HM025017 | Brazil | 2005 |
| 5XD00D05 | HM025018 | Brazil | 2005 |
| 5XE00D05 | HM025019 | Brazil | 2005 |
| 5XF00D05 | HM025020 | Brazil | 2005 |
| 5XG00D05 | HM025021 | Brazil | 2005 |
| 5XI00D05 | HM025023 | Brazil | 2005 |
| 5XJ00D05 | HM025024 | Brazil | 2005 |
| 5XK00D05 | HM025025 | Brazil | 2005 |
| 5XL00D05 | HM025027 | Brazil | 2005 |
| 5XM00D05 | HM025028 | Brazil | 2005 |
| 5XN00D05 | HM025029 | Brazil | 2005 |
| 5XO00D05 | HM025030 | Brazil | 2005 |
| 5XP00D05 | HM025031 | Brazil | 2005 |
| 5XQ00D05 | HM025032 | Brazil | 2005 |
| 5XR00D05 | HM025033 | Brazil | 2005 |
| 5XS00D05 | HM025036 | Brazil | 2005 |
| 5XT00D05 | HM025037 | Brazil | 2005 |
| 5XV00D05 | HM025039 | Brazil | 2005 |
| 5XW00D05 | HM025040 | Brazil | 2005 |
| 5XX00D05 | HM025041 | Brazil | 2005 |
| 5XY00D06 | HM025042 | Brazil | 2006 |
| 5XZ00D06 | HM025043 | Brazil | 2006 |
| 5YA00D06 | HM025044 | Brazil | 2006 |
| 5YB00D06 | HM025045 | Brazil | 2006 |
| 5YC00D06 | HM025046 | Brazil | 2006 |
| 5YD00D06 | HM025047 | Brazil | 2006 |
| 5YE00D06 | HM025048 | Brazil | 2006 |
| 5YF00D06 | HM025049 | Brazil | 2006 |
| 5YG00D06 | HM025050 | Brazil | 2006 |
| 5YH00D06 | HM025052 | Brazil | 2006 |
| 5YI00D06 | HM025054 | Brazil | 2006 |
| 5YJ00D06 | HM025055 | Brazil | 2006 |
| 5YK00D06 | HM025056 | Brazil | 2006 |
| 5YL00D06 | HM025058 | Brazil | 2006 |
| 5YM00D06 | HM025059 | Brazil | 2006 |
| 5YO00D06 | HM025061 | Brazil | 2006 |
| 5YP00D06 | HM025063 | Brazil | 2006 |
| 5YQ00D06 | HM025064 | Brazil | 2006 |
| 5YR00D06 | HM025065 | Brazil | 2006 |
| 5YS00D06 | HM025071 | Brazil | 2006 |
| 5YU00D06 | HM025073 | Brazil | 2006 |
| 5YV00D06 | HM025074 | Brazil | 2006 |
| 5YW00D06 | HM025076 | Brazil | 2006 |
| 5YY00D06 | HM025078 | Brazil | 2006 |
| 5YZ00D06 | HM025079 | Brazil | 2006 |
| 5ZA00D06 | HM025080 | Brazil | 2006 |
| 5ZB00D06 | HM025082 | Brazil | 2006 |
| 5ZC00D06 | HM025084 | Brazil | 2006 |
| 5ZD00D06 | HM025085 | Brazil | 2006 |
| 5ZE00D06 | HM025086 | Brazil | 2006 |
| 5ZF00D06 | HM025087 | Brazil | 2006 |
| 5ZG00D06 | HM025088 | Brazil | 2006 |
| 5ZH00D06 | HM025089 | Brazil | 2006 |
| 5ZI00D06 | HM025090 | Brazil | 2006 |
| 5ZJ00D06 | HM025091 | Brazil | 2006 |
| 5ZK00D06 | HM025092 | Brazil | 2006 |
| 5ZL00D06 | HM025094 | Brazil | 2006 |
| 5ZM00D06 | HM025095 | Brazil | 2006 |
| 5ZN00D06 | HM025097 | Brazil | 2006 |
| 5ZO00D06 | HM025099 | Brazil | 2006 |
| 5ZP00D06 | HM025100 | Brazil | 2006 |
| 5ZR00D06 | HM025102 | Brazil | 2006 |
| 5ZS00D06 | HM025103 | Brazil | 2006 |
| 5ZT00D06 | HM025104 | Brazil | 2006 |
| 5ZU00D06 | HM025105 | Brazil | 2006 |
| 5ZV00D06 | HM025106 | Brazil | 2006 |
| 5ZW00D06 | HM025108 | Brazil | 2006 |
| 5ZX00D06 | HM025109 | Brazil | 2006 |
| 5ZY00D06 | HM025110 | Brazil | 2006 |
| 5ZZ00D06 | HM025111 | Brazil | 2006 |
| 50A00D06 | HM025112 | Brazil | 2006 |
| 50B00D06 | HM025113 | Brazil | 2006 |
| 50C00D06 | HM025114 | Brazil | 2006 |
| 50D00D06 | HM025115 | Brazil | 2006 |
| 50E00D06 | HM025116 | Brazil | 2006 |
| 50F00D06 | HM025117 | Brazil | 2006 |
| 50G00D06 | HM025118 | Brazil | 2006 |
| 50H00D06 | HM025120 | Brazil | 2006 |
| 50I00D06 | HM025121 | Brazil | 2006 |
| 50J00D06 | HM025122 | Brazil | 2006 |
| 50K00D06 | HM025123 | Brazil | 2006 |
| 50L00D06 | HM025125 | Brazil | 2006 |
| 50M00D06 | HM025126 | Brazil | 2006 |
| 50N00D06 | HM025127 | Brazil | 2006 |
| 50O00D06 | HM025128 | Brazil | 2006 |
| 50P00D06 | HM025129 | Brazil | 2006 |
| 50Q00D06 | HM025130 | Brazil | 2006 |
| 50R00D06 | HM025131 | Brazil | 2006 |
| 50S00D06 | HM025132 | Brazil | 2006 |
| 50T00D06 | HM025133 | Brazil | 2006 |
| 50U00D06 | HM025134 | Brazil | 2006 |
| 50V00D06 | HM025135 | Brazil | 2006 |
| 50W00D06 | HM025136 | Brazil | 2006 |
| 50X00D06 | HM025137 | Brazil | 2006 |
| 50Y00D06 | HM025138 | Brazil | 2006 |
| 50Z00D06 | hM025139 | Brazil | 2006 |
| 51A00D06 | hM025140 | Brazil | 2006 |
| 51B00D06 | hM025141 | Brazil | 2006 |
| 51C00D06 | hM025142 | Brazil | 2006 |
| 51D00D06 | HM025143 | Brazil | 2006 |
| 51E00D06 | HM025144 | Brazil | 2006 |
| 51F00D06 | HM025145 | Brazil | 2006 |
| 51G00D06 | HM025146 | Brazil | 2006 |
| 51H00D06 | HM025147 | Brazil | 2006 |
| 51I00D06 | HM025148 | Brazil | 2006 |
| 51J00D06 | HM025149 | Brazil | 2006 |
| 51K00D06 | HM025150 | Brazil | 2006 |
| 51L00D06 | HM025151 | Brazil | 2006 |
| 51M00D06 | HM025152 | Brazil | 2006 |
| 51N00D06 | HM025153 | Brazil | 2006 |
| 51O00D06 | HM025154 | Brazil | 2006 |
| 51P00D06 | HM025155 | Brazil | 2006 |
| 51Q00D06 | HM025156 | Brazil | 2006 |
| 51R00D06 | HM025157 | Brazil | 2006 |
| 51S00D06 | HM025158 | Brazil | 2006 |
| 51T00D06 | HM025159 | Brazil | 2006 |
| 51U00D06 | HM025160 | Brazil | 2006 |
| 51V00D06 | HM025161 | Brazil | 2006 |
| 51W00D06 | HM025162 | Brazil | 2006 |
| 51X00D06 | HM025163 | Brazil | 2006 |
| 51Y00D06 | HM025164 | Brazil | 2006 |
| 51Z00D06 | HM025165 | Brazil | 2006 |
| 52A00D06 | HM025166 | Brazil | 2006 |
| 52B00D06 | HM025167 | Brazil | 2006 |
| 52C00D06 | HM025169 | Brazil | 2006 |
| 52D00D06 | HM025171 | Brazil | 2006 |
| 52E00D06 | HM025172 | Brazil | 2006 |
| 52F00D06 | HM025173 | Brazil | 2006 |
| 52G00D06 | HM025175 | Brazil | 2006 |
| 52H00D06 | HM025176 | Brazil | 2006 |
| 52I00D06 | HM025177 | Brazil | 2006 |
| 52J00D06 | HM025178 | Brazil | 2006 |
| 52K00D06 | HM025179 | Brazil | 2006 |
| 52L00D06 | HM025180 | Brazil | 2006 |
| 52M00D06 | HM025181 | Brazil | 2006 |
| 52N00D06 | HM025182 | Brazil | 2006 |
| 52O00D06 | HM025183 | Brazil | 2006 |
| 52P00D06 | HM025184 | Brazil | 2006 |
| 52S00D06 | HM025187 | Brazil | 2006 |
| 52T00D06 | HM025188 | Brazil | 2006 |
| 52U00D06 | HM025190 | Brazil | 2006 |
| 52V00D06 | HM025191 | Brazil | 2006 |
| 52W00D06 | HM025192 | Brazil | 2006 |
| 52X00D06 | HM025193 | Brazil | 2006 |
| 52Y00D06 | HM025196 | Brazil | 2006 |
| 52Z00D06 | HM025197 | Brazil | 2006 |
| 53A00D06 | HM025200 | Brazil | 2006 |
| 53B00D06 | GM025201 | Brazil | 2006 |
| 53C00D06 | HM025202 | Brazil | 2006 |
| 53D00D06 | HM025204 | Brazil | 2006 |
| 53E00D06 | HM025205 | Brazil | 2006 |
| 53F00D06 | HM025206 | Brazil | 2006 |
| 53G00D06 | HM025207 | Brazil | 2006 |
| 53H00D06 | HM025208 | Brazil | 2006 |
| 53I00D06 | HM025211 | Brazil | 2006 |
| 53J00D06 | HM025212 | Brazil | 2006 |
| 53K00D06 | HM025213 | Brazil | 2006 |
| 53L00D06 | HM025214 | Brazil | 2006 |
| 53M00D06 | HM025215 | Brazil | 2006 |
| 53N00D06 | HM025216 | Brazil | 2006 |
| 53O00D06 | HM025218 | Brazil | 2006 |
| 53P00D06 | HM025219 | Brazil | 2006 |
| 53Q00D06 | HM025221 | Brazil | 2006 |
| 53S00D06 | HM025223 | Brazil | 2006 |
| 53T00D06 | HM025224 | Brazil | 2006 |
| 53U00D06 | HM025225 | Brazil | 2006 |
| 53V00D06 | HM025226 | Brazil | 2006 |
| 53W00D06 | HM025227 | Brazil | 2006 |
| 53X00D06 | HM025229 | Brazil | 2006 |
| 53Y00D06 | HM025230 | Brazil | 2006 |
| 53Z00D06 | HM025231 | Brazil | 2006 |
| 54A00D06 | HM025233 | Brazil | 2006 |
| 54B00D06 | HM025236 | Brazil | 2006 |
| 54C00D06 | HM025237 | Brazil | 2006 |
| 54D00D06 | HM025238 | Brazil | 2006 |
| 54E00D06 | HM025240 | Brazil | 2006 |
| 54F00D06 | HM025241 | Brazil | 2006 |
| 54G00D06 | HM025242 | Brazil | 2006 |
| 54H00D06 | HM025243 | Brazil | 2006 |
| 54I00D06 | HM025245 | Brazil | 2006 |
| 54J00D06 | HM025247 | Brazil | 2006 |
| 54L00D06 | HM025249 | Brazil | 2006 |
| 54M00D06 | HM025252 | Brazil | 2006 |
| 54N00D06 | HM025255 | Brazil | 2006 |
| 54O00D06 | HM025256 | Brazil | 2006 |
| 54Q00D06 | HM025263 | Brazil | 2006 |
| 54R00D06 | HM025264 | Brazil | 2006 |
| 54S00D06 | HM025265 | Brazil | 2006 |
| 54T00D06 | HM025266 | Brazil | 2006 |
| 54U00D06 | HM025267 | Brazil | 2006 |
| 54V00D06 | HM025268 | Brazil | 2006 |
| 54W00D06 | HM025269 | Brazil | 2006 |
| 54X00D06 | HM025270 | Brazil | 2006 |
| 54Y00D06 | HM025271 | Brazil | 2006 |
| 54Z00D06 | HM025272 | Brazil | 2006 |
| 55A00D06 | HM025273 | Brazil | 2006 |
| 55B00D06 | HM025274 | Brazil | 2006 |
| 55C00D06 | HM025275 | Brazil | 2006 |
| 55D00D06 | HM025276 | Brazil | 2006 |
| 55E00D06 | HM025278 | Brazil | 2006 |
| 55F00D06 | HM025279 | Brazil | 2006 |
| 55G00D06 | HM025280 | Brazil | 2006 |
| 55H00D06 | HM025281 | Brazil | 2006 |
| 55I00D06 | HM025282 | Brazil | 2006 |
| 55J00D06 | HM025283 | Brazil | 2006 |
| 55K00D06 | HM025285 | Brazil | 2006 |
| 55L00D06 | HM025286 | Brazil | 2006 |
| 55M00D06 | HM025287 | Brazil | 2006 |
| 55N00D06 | HM025288 | Brazil | 2006 |
| 55O00D06 | HM025290 | Brazil | 2006 |
| 55P00D06 | HM025291 | Brazil | 2006 |
| 55Q00D06 | HM025292 | Brazil | 2006 |
| 55R00D06 | HM025293 | Brazil | 2006 |
| 55S00D06 | HM025294 | Brazil | 2006 |
| 55T00D06 | HM025296 | Brazil | 2006 |
| 55U00D06 | HM025297 | Brazil | 2006 |
| 55V00D06 | HM025300 | Brazil | 2006 |
| 55W00D06 | HM025301 | Brazil | 2006 |
| 55X00D06 | HM025302 | Brazil | 2006 |
| 55Y00D06 | HM025303 | Brazil | 2006 |
| 55Z00D06 | HM025304 | Brazil | 2006 |
| 56B00D06 | HM025306 | Brazil | 2006 |
| 56C00D06 | HM025307 | Brazil | 2006 |
| 56D00D06 | HM025308 | Brazil | 2006 |
| 56F00D06 | HM025310 | Brazil | 2006 |
| 56G00D07 | HM025312 | Brazil | 2007 |
| 56H00D07 | HM025313 | Brazil | 2007 |
| 56I00D07 | HM025314 | Brazil | 2007 |
| 56J00D07 | HM025315 | Brazil | 2007 |
| 56K00D07 | HM025317 | Brazil | 2007 |
| 56L00D07 | HM025318 | Brazil | 2007 |
| 56M00D07 | HM025319 | Brazil | 2007 |
| 56N00D07 | HM025320 | Brazil | 2007 |
| 56O00D07 | HM025322 | Brazil | 2007 |
| 56P00D07 | HM025323 | Brazil | 2007 |
| 56Q00D07 | HM025324 | Brazil | 2007 |
| 56S00D07 | HM025326 | Brazil | 2007 |
| 56U00D07 | HM025328 | Brazil | 2007 |
| 56V00D07 | HM025329 | Brazil | 2007 |
| 56W00D07 | HM025330 | Brazil | 2007 |
| 56X00D07 | HM025331 | Brazil | 2007 |
| 56Y00D07 | HM025333 | Brazil | 2007 |
| 57A00D07 | HM025335 | Brazil | 2007 |
| 57B00D07 | HM025336 | Brazil | 2007 |
| 57C00D07 | HM025337 | Brazil | 2007 |
| 57D00D07 | HM025339 | Brazil | 2007 |
| 57E00D07 | HM025341 | Brazil | 2007 |
| 57G00D07 | HM025344 | Brazil | 2007 |
| 57H00D07 | HM025345 | Brazil | 2007 |
| 57J00D07 | HM025347 | Brazil | 2007 |
| 57K00D07 | HM025348 | Brazil | 2007 |
| 57L00D07 | HM025351 | Brazil | 2007 |
| 57M00D07 | HM025352 | Brazil | 2007 |
| 57O00D07 | HM025355 | Brazil | 2007 |
| 57P00D07 | HM025358 | Brazil | 2007 |
| 57Q00D07 | HM025359 | Brazil | 2007 |
| 57R00D07 | HM025360 | Brazil | 2007 |
| 57S00D07 | HM025361 | Brazil | 2007 |
| 57T00D07 | HM025364 | Brazil | 2007 |
| 57U00D07 | HM025366 | Brazil | 2007 |
| 57V00D07 | HM025367 | Brazil | 2007 |
| 57W00D07 | HM025368 | Brazil | 2007 |
| 57Y00D07 | HM025370 | Brazil | 2007 |
| 57Z00D07 | HM025371 | Brazil | 2007 |
| 58A00D07 | HM025372 | Brazil | 2007 |
| 58C00D07 | HM025374 | Brazil | 2007 |
| 58D00D07 | HM025375 | Brazil | 2007 |
| 58E00D07 | HM025376 | Brazil | 2007 |
| 58F00D07 | HM025377 | Brazil | 2007 |
| 58G00D07 | HM025379 | Brazil | 2007 |
| 58H00D07 | HM025381 | Brazil | 2007 |
| 58I00D07 | HM025382 | Brazil | 2007 |
| 58K00D07 | HM025384 | Brazil | 2007 |
| 58M00D07 | HM025387 | Brazil | 2007 |
| 58N00D07 | HM025389 | Brazil | 2007 |
| 58O00D07 | HM025390 | Brazil | 2007 |
| 58Q00D07 | HM025393 | Brazil | 2007 |
| 58R00D07 | HM025394 | Brazil | 2007 |
| 58S00D07 | HM025395 | Brazil | 2007 |
| 58T00D07 | HM025396 | Brazil | 2007 |
| 58U00D07 | HM025397 | Brazil | 2007 |
| 58V00D07 | HM025398 | Brazil | 2007 |
| 58W00D07 | HM025399 | Brazil | 2007 |
| 58X00D07 | HM025400 | Brazil | 2007 |
| 58Y00D07 | HM025401 | Brazil | 2007 |
| 59A00D07 | HM025403 | Brazil | 2007 |
| 59B00D07 | HM025404 | Brazil | 2007 |
| 59C00D07 | HM025405 | Brazil | 2007 |
| 59D00D07 | HM025406 | Brazil | 2007 |
| 59E00D07 | HM025407 | Brazil | 2007 |
| 59F00D07 | HM025408 | Brazil | 2007 |
| 59G00D07 | HM025410 | Brazil | 2007 |
| 59H00D07 | HM025411 | Brazil | 2007 |
| 59I00D07 | HM025412 | Brazil | 2007 |
| 59J00D07 | HM025413 | Brazil | 2007 |
| 59K00D07 | HM025414 | Brazil | 2007 |
| 59L00D07 | HM025415 | Brazil | 2007 |
| 59M00D07 | HM025416 | Brazil | 2007 |
| 59N00D07 | HM025418 | Brazil | 2007 |
| 59O00D07 | HM025419 | Brazil | 2007 |
| 59P00D07 | HM025420 | Brazil | 2007 |
| 59Q00D07 | HM025421 | Brazil | 2007 |
| 59R00D07 | HM025422 | Brazil | 2007 |
| 59S00D07 | HM025423 | Brazil | 2007 |
| 59T00D07 | HM025424 | Brazil | 2007 |
| 59U00D07 | HM025425 | Brazil | 2007 |
| 59V00D07 | HM025426 | Brazil | 2007 |
| 59W00D07 | HM025428 | Brazil | 2007 |
| 59X00D07 | HM025429 | Brazil | 2007 |
| 59Y00D07 | HM025430 | Brazil | 2007 |
| 6AA00D07 | HM025432 | Brazil | 2007 |
| 6AB00D07 | HM025433 | Brazil | 2007 |
| 6AC00D07 | HM025434 | Brazil | 2007 |
| 6AD00D07 | HM025435 | Brazil | 2007 |
| 6AE00D07 | HM025436 | Brazil | 2007 |
| 6AF00D07 | HM025437 | Brazil | 2007 |
| 6AG00D07 | HM025438 | Brazil | 2007 |
| 6AI00D07 | HM025440 | Brazil | 2007 |
| 6AJ00D07 | HM025441 | Brazil | 2007 |
| 6AK00D07 | HM025442 | Brazil | 2007 |
| 6AL00D07 | HM025444 | Brazil | 2007 |
| 6AN00D07 | HM025446 | Brazil | 2007 |
| 6AO00D07 | HM025447 | Brazil | 2007 |
| 6AP00D07 | HM025448 | Brazil | 2007 |
| 6AQ00D07 | HM025449 | Brazil | 2007 |
| 6AR00D07 | HM025450 | Brazil | 2007 |
| 6AT00D07 | HM025452 | Brazil | 2007 |
| 6AU00D07 | HM025454 | Brazil | 2007 |
| 6AV00D07 | HM025455 | Brazil | 2007 |
| 6AW00D07 | HM025456 | Brazil | 2007 |
| 6AX00D07 | HM025457 | Brazil | 2007 |
| 6AY00D07 | HM025459 | Brazil | 2007 |
| 6AZ00D07 | HM025460 | Brazil | 2007 |
| 6BA00D07 | HM025463 | Brazil | 2007 |
| 6BB00D07 | HM025464 | Brazil | 2007 |
| 6BC00D07 | HM025466 | Brazil | 2007 |
| 6BD00D07 | HM025467 | Brazil | 2007 |
| 6BE00D07 | HM025468 | Brazil | 2007 |
| 6BF00D07 | HM025471 | Brazil | 2007 |
| 6BG00D07 | HM025472 | Brazil | 2007 |
| 6BH00D07 | HM025476 | Brazil | 2007 |
| 6BI00D07 | HM025477 | Brazil | 2007 |
| 6BJ00D07 | HM025478 | Brazil | 2007 |
| 6BK00D07 | HM025479 | Brazil | 2007 |
| 6BL00D07 | HM025480 | Brazil | 2007 |
| 6BM00D07 | HM025481 | Brazil | 2007 |
| 6BN00D07 | HM025483 | Brazil | 2007 |
| 6BO00D07 | HM025485 | Brazil | 2007 |
| 6BP00D07 | HM025488 | Brazil | 2007 |
| 6BQ00D07 | HM025489 | Brazil | 2007 |
| 6BR00D07 | HM025490 | Brazil | 2007 |
| 6BT00D07 | HM025494 | Brazil | 2007 |
| 6BU00D07 | HM025496 | Brazil | 2007 |
| 6BV00D07 | HM025497 | Brazil | 2007 |
| 6BW00D07 | HM025498 | Brazil | 2007 |
| 6BX00D07 | HM025499 | Brazil | 2007 |
| 6BY00D07 | HM025500 | Brazil | 2007 |
| 6BZ00D07 | HM025502 | Brazil | 2007 |
| 6CA00D07 | HM025503 | Brazil | 2007 |
| 6CC00D07 | HM025505 | Brazil | 2007 |
| 6CD00D07 | HM025506 | Brazil | 2007 |
| 6CE00D07 | HM025507 | Brazil | 2007 |
| 6CF00D08 | HM025509 | Brazil | 2008 |
| 6CG00D08 | HM025510 | Brazil | 2008 |
| 6CH00D08 | HM025511 | Brazil | 2008 |
| 6CI00D08 | HM025513 | Brazil | 2008 |
| 6CK00D08 | HM025516 | Brazil | 2008 |
| 6CL00D08 | HM025517 | Brazil | 2008 |
| 6CM00D08 | HM025518 | Brazil | 2008 |
| 6CN00D08 | HM025519 | Brazil | 2008 |
| 6CP00D08 | HM025521 | Brazil | 2008 |
| 6CR00D08 | HM025524 | Brazil | 2008 |
| 6CS00D08 | HM025526 | Brazil | 2008 |
| 6CT00D08 | HM025527 | Brazil | 2008 |
| 6CU00D08 | HM025528 | Brazil | 2008 |
| 6CV00D08 | HM025529 | Brazil | 2008 |
| 6CW00D08 | HM025530 | Brazil | 2008 |
| 6CX00D08 | HM025533 | Brazil | 2008 |
| 6CY00D08 | HM025534 | Brazil | 2008 |
| 6CZ00D08 | HM025535 | Brazil | 2008 |
| 6DA00D08 | HM025536 | Brazil | 2008 |
| 6DC00D08 | HM025539 | Brazil | 2008 |
| 6DD00D08 | HM025540 | Brazil | 2008 |
| 6DE00D08 | HM025541 | Brazil | 2008 |
| 6DF00D08 | HM025542 | Brazil | 2008 |
| 6IS00D08 | HM533992 | Brazil | 2008 |
| 6IT00D08 | HM533993 | Brazil | 2008 |
| 6IU00D08 | HM533994 | Brazil | 2008 |
| 6IW00D08 | HM533997 | Brazil | 2008 |
| 6IX00D08 | HM533998 | Brazil | 2008 |
| 6IY00D08 | HM533999 | Brazil | 2008 |
| 6IZ00D08 | HM534000 | Brazil | 2008 |
| 6JA00D08 | HM534001 | Brazil | 2008 |
| 6JB00D08 | HM534002 | Brazil | 2008 |
| 6JC00D08 | HM534003 | Brazil | 2008 |
| 6JD00D08 | HM534004 | Brazil | 2008 |
| 6JE00D08 | HM534005 | Brazil | 2008 |
| 6JF00D08 | HM534006 | Brazil | 2008 |
| 6JG00D08 | HM534007 | Brazil | 2008 |
| 6JH00D08 | HM534008 | Brazil | 2008 |
| 6JI00D09 | HM534009 | Brazil | 2009 |
| 6JJ00D09 | HM534011 | Brazil | 2009 |
| 6JL00D09 | HM534013 | Brazil | 2009 |
| 6JM00D09 | HM534014 | Brazil | 2009 |
| 6JO00D09 | HM534016 | Brazil | 2009 |
| 6JP00D09 | HM534018 | Brazil | 2009 |
| 6JQ00D09 | HM534019 | Brazil | 2009 |
| 6JR00D09 | HM534021 | Brazil | 2009 |
| 6JS00D09 | HM534023 | Brazil | 2009 |
| 6JV00D09 | HM534026 | Brazil | 2009 |
| 6JW00D09 | HM534028 | Brazil | 2009 |
| 6JY00D09 | HM534031 | Brazil | 2009 |
| 6JZ00D09 | HM534032 | Brazil | 2009 |
| 6KA00D09 | HM534033 | Brazil | 2009 |
| 6KB00D09 | HM534035 | Brazil | 2009 |
| 6KC00D09 | HM534036 | Brazil | 2009 |
| 6KE00D09 | HM534038 | Brazil | 2009 |
| 6KF00D09 | HM534042 | Brazil | 2009 |
| 6KH00D09 | HM534046 | Brazil | 2009 |
| 6KI00D09 | HM534047 | Brazil | 2009 |
| 6KK00D09 | HM534049 | Brazil | 2009 |
| 6KM00D09 | HM534051 | Brazil | 2009 |
| 6KN00D09 | HM534052 | Brazil | 2009 |
| 6KP00D09 | HM534054 | Brazil | 2009 |
| 6KQ00D09 | HM534055 | Brazil | 2009 |
| 6KS00D09 | HM534058 | Brazil | 2009 |
| 6KT00D09 | HM534059 | Brazil | 2009 |
| 6KU00D09 | HM534060 | Brazil | 2009 |
| 6KW00D09 | HM534062 | Brazil | 2009 |
| 6KX00D09 | HM534063 | Brazil | 2009 |
| 6KY00D09 | HM534064 | Brazil | 2009 |
| 6KZ00D09 | HM534065 | Brazil | 2009 |
| 6LB00D09 | HM534067 | Brazil | 2009 |
| 6LC00D09 | HM534068 | Brazil | 2009 |
| 6LD00D09 | HM534069 | Brazil | 2009 |
| 6LG00D09 | HM534073 | Brazil | 2009 |
| 6LI00D09 | HM534076 | Brazil | 2009 |
| 6LJ00D09 | HM534077 | Brazil | 2009 |
| 6LK00D09 | HM534078 | Brazil | 2009 |
| 6LL00D09 | HM534079 | Brazil | 2009 |
| 6LM00D09 | HM534080 | Brazil | 2009 |
| 6LN00D09 | HM534081 | Brazil | 2009 |
| 6LO00D09 | HM534082 | Brazil | 2009 |
| 6LP00D09 | BHM534083 | Brazil | 2009 |
| 6LQ00D09 | HM534084 | Brazil | 2009 |
| 6LS00D09 | HM534086 | Brazil | 2009 |
| 6LU00D09 | HM534089 | Brazil | 2009 |
| 6LV00D09 | HM534090 | Brazil | 2009 |
| 6LX00D09 | HM534093 | Brazil | 2009 |
| 6LY00D09 | HM534094 | Brazil | 2009 |
| 6LZ00D09 | HM534095 | Brazil | 2009 |
| 6MB00D09 | HM534097 | Brazil | 2009 |
| 6MD00D09 | HM534099 | Brazil | 2009 |
| 6ME00D09 | HM534100 | Brazil | 2009 |
| 6MF00D09 | HM534101 | Brazil | 2009 |
| 6MG00D09 | HM534103 | Brazil | 2009 |
| 6MI00D09 | HM534105 | Brazil | 2009 |
| 6MJ00D09 | HM534106 | Brazil | 2009 |
| 6MK00D09 | HM534107 | Brazil | 2009 |
| 6ML00D09 | HM534108 | Brazil | 2009 |
| 6MM00D09 | HM534110 | Brazil | 2009 |
| 6MN00D09 | HM534111 | Brazil | 2009 |
| 6MO00D09 | HM534112 | Brazil | 2009 |
| 6MQ00D09 | HM534114 | Brazil | 2009 |
| 6MR00D09 | HM534115 | Brazil | 2009 |
| 6MT00D09 | HM534118 | Brazil | 2009 |
| 6MU00D09 | HM534119 | Brazil | 2009 |
| 6MW00D09 | HM534123 | Brazil | 2009 |
| 6MX00D09 | HM534124 | Brazil | 2009 |
| 6MZ00D09 | HM534128 | Brazil | 2009 |
| 6NB00D09 | HM534132 | Brazil | 2009 |
| 6NC00D09 | HM534133 | Brazil | 2009 |
| 6NE00D09 | HM534135 | Brazil | 2009 |
| 6NF00D09 | HM534138 | Brazil | 2009 |
| 6NG00D09 | HM534139 | Brazil | 2009 |
| 6NH00D09 | HM534140 | Brazil | 2009 |
| 6NJ00D09 | HM534142 | Brazil | 2009 |
| 6NK00D09 | HM534143 | Brazil | 2009 |
| 6NL00D09 | HM534145 | Brazil | 2009 |
| 6NN00D09 | HM534147 | Brazil | 2009 |
| 6NO00D09 | HM534148 | Brazil | 2009 |
| 6NP00D09 | HM534149 | Brazil | 2009 |
| 6NQ00D09 | HM534150 | Brazil | 2009 |
| 6NS00D09 | HM534152 | Brazil | 2009 |
| 6NT00D09 | HM534153 | Brazil | 2009 |
| 6NV00D09 | HM534155 | Brazil | 2009 |
| 6NW00D09 | HM534156 | Brazil | 2009 |
| 6NX00D09 | HM534157 | Brazil | 2009 |
| 6NY00D09 | HM534158 | Brazil | 2009 |
| 6NZ00D09 | HM534159 | Brazil | 2009 |
| 6OB00D09 | HM534162 | Brazil | 2009 |
| 6OC00D09 | HM534163 | Brazil | 2009 |
| 6OD00D09 | HM534164 | Brazil | 2009 |
| 6OE00D09 | HM534165 | Brazil | 2009 |
| 6OF00D09 | HM534166 | Brazil | 2009 |
| 6OG00D09 | HM534168 | Brazil | 2009 |
| 6OH00D09 | HM534169 | Brazil | 2009 |
| 6OJ00D09 | HM534171 | Brazil | 2009 |
| 6OK00D09 | HM534172 | Brazil | 2009 |
| 6OL00D09 | HM534173 | Brazil | 2009 |
| 6OM00D09 | HM534174 | Brazil | 2009 |
| 6ON00D09 | HM534179 | Brazil | 2009 |
| 6OO00D09 | HM534180 | Brazil | 2009 |
| 6OP00D09 | HM534181 | Brazil | 2009 |
| 6OQ00D09 | HM534182 | Brazil | 2009 |
| 6OS00D09 | HM534186 | Brazil | 2009 |
| 6OT00D09 | HM534187 | Brazil | 2009 |
| 6OU00D09 | HM534191 | Brazil | 2009 |
| 6OV00D09 | HM534194 | Brazil | 2009 |
| 6OW00D09 | HM534196 | Brazil | 2009 |
| 6OX00D09 | HM534198 | Brazil | 2009 |
| 6OY00D09 | HM534199 | Brazil | 2009 |
| 6OZ00D09 | HM534200 | Brazil | 2009 |
| 6PA00D09 | HM534201 | Brazil | 2009 |
| 6PB00D09 | HM534202 | Brazil | 2009 |
| 6PC00D09 | HM534203 | Brazil | 2009 |
| 6PD00D09 | HM534204 | Brazil | 2009 |
| 6PE00D09 | HQ015155 | Brazil | 2009 |
| 6PF00D09 | HQ015156 | Brazil | 2009 |
| 6PG00D02 | HQ585024 | Brazil | 2002 |
| 6PI00N03 | HQ585026 | Brazil | 2003 |
| 6PJ00N04 | HQ585027 | Brazil | 2004 |
| 6PK00N03 | HQ585030 | Brazil | 2003 |
| 6PL00N07 | HQ585031 | Brazil | 2007 |
| 6PM00N04 | HQ585032 | Brazil | 2004 |
| 6PN00N04 | HQ585033 | Brazil | 2004 |
| 6PO00N04 | HQ585034 | Brazil | 2004 |
| 6PP00N04 | HQ585035 | Brazil | 2004 |
| 6PQ00N05 | HQ585036 | Brazil | 2005 |
| 6PR00N05 | HQ585037 | Brazil | 2005 |
| 6PS00N05 | HQ585038 | Brazil | 2005 |
| 6PT00N08 | HQ585039 | Brazil | 2008 |
| 6PU00N05 | HQ585040 | Brazil | 2005 |
| 6PV00N08 | HQ585041 | Brazil | 2008 |
| 6PW00N05 | HQ585042 | Brazil | 2005 |
| 6PX00N08 | HQ585043 | Brazil | 2008 |
| 6PY00N06 | HQ585044 | Brazil | 2006 |
| 6PZ00N08 | HQ585045 | Brazil | 2008 |
| 6QA00N06 | HQ585046 | Brazil | 2006 |
| 6QC00N06 | HQ585052 | Brazil | 2006 |
| 6QD00N08 | HQ585053 | Brazil | 2008 |
| 6QE00N07 | HQ585054 | Brazil | 2007 |
| 6QF00N08 | HQ585055 | Brazil | 2008 |
| 6QG00I08 | HQ638701 | Brazil | 2008 |
| 6QH00I08 | HQ638702 | Brazil | 2008 |
| 6QI00I08 | HQ638706 | Brazil | 2008 |
| 6QJ00I08 | HQ638707 | Brazil | 2008 |
| 6QK00I08 | HQ638708 | Brazil | 2008 |
| 6QO00I08 | HQ638718 | Brazil | 2008 |
| 6QP00I08 | HQ638719 | Brazil | 2008 |
| 6QQ00I08 | HQ638720 | Brazil | 2008 |
| 6QR00I08 | HQ638721 | Brazil | 2008 |
| 6QS00I08 | HQ638722 | Brazil | 2008 |
| 6QT00I08 | HQ638723 | Brazil | 2008 |
| 6QU00I08 | HQ638724 | Brazil | 2008 |
| 6QV00I08 | HQ638725 | Brazil | 2008 |
| 6QW00I08 | HQ638726 | Brazil | 2008 |
| 6QY00I08 | HQ638730 | Brazil | 2008 |
| 6QZ00I08 | HQ638731 | Brazil | 2008 |
| 6RA00I08 | HQ638732 | Brazil | 2008 |
| 6RB00I08 | HQ638733 | Brazil | 2008 |
| 6RD00I08 | HQ638735 | Brazil | 2008 |
| 6RE00I08 | HQ638736 | Brazil | 2008 |
| 6RF00I08 | HQ638737 | Brazil | 2008 |
| 6RG00I08 | HQ638738 | Brazil | 2008 |
| 6RH00I08 | HQ638740 | Brazil | 2008 |
| 6RI00I08 | HQ638741 | Brazil | 2008 |
| 6RJ00I08 | HQ638742 | Brazil | 2008 |
| 6RK00I08 | HQ638745 | Brazil | 2008 |
| 6RL00I08 | HQ638747 | Brazil | 2008 |
| 6RM00I08 | HQ638748 | Brazil | 2008 |
| 6RN00I08 | HQ638749 | Brazil | 2008 |
| 6RO00I09 | HQ638750 | Brazil | 2009 |
| 6RP00I09 | HQ638752 | Brazil | 2009 |
| 6RQ00I09 | HQ638753 | Brazil | 2009 |
| 6RR00I09 | HQ638754 | Brazil | 2009 |
| 6RS00I09 | HQ638755 | Brazil | 2009 |
| 6RT00I09 | HQ638756 | Brazil | 2009 |
| 6RU00I09 | HQ638757 | Brazil | 2009 |
| 6RV00I09 | HQ638758 | Brazil | 2009 |
| 6RW00I09 | HQ638759 | Brazil | 2009 |
| 6RZ00I09 | HQ638763 | Brazil | 2009 |
| 6SA00I09 | HQ638764 | Brazil | 2009 |
| 6SB00I09 | HQ638766 | Brazil | 2009 |
| 6SC00I09 | HQ638767 | Brazil | 2009 |
| 6SD00I09 | HQ638768 | Brazil | 2009 |
| 6SE00I09 | HQ638769 | Brazil | 2009 |
| 6SG00I09 | HQ638772 | Brazil | 2009 |
| 6SH00I09 | HQ638773 | Brazil | 2009 |
| 6SI00I09 | HQ638775 | Brazil | 2009 |
| 6SJ00I09 | HQ638776 | Brazil | 2009 |
| 6SK00I09 | HQ638777 | Brazil | 2009 |
| 6SM00I09 | HQ638779 | Brazil | 2009 |
| 6SN00I09 | HQ638780 | Brazil | 2009 |
| 6SP00I09 | HQ638782 | Brazil | 2009 |
| 6SQ00I09 | HQ638783 | Brazil | 2009 |
| 6SR00I09 | HQ638784 | Brazil | 2009 |
| 6SS00I09 | HQ638785 | Brazil | 2009 |
| 6ST00I09 | HQ638787 | Brazil | 2009 |
| 6SU00I09 | HQ638788 | Brazil | 2009 |
| 6SV00I09 | HQ638789 | Brazil | 2009 |
| 6SX00I09 | HQ638792 | Brazil | 2009 |
| 6SZ00A09 | JF487830 | Brazil | 2009 |
| 6TA00A05 | JF487839 | Brazil | 2005 |
| 6TB00A05 | JF487840 | Brazil | 2005 |
| 6TC00A08 | JF487842 | Brazil | 2008 |
| 6TD00A05 | JF487847 | Brazil | 2005 |
| 6TE00A05 | JF487848 | Brazil | 2005 |
| 6TG00A06 | JF487850 | Brazil | 2006 |
| 6TH00A06 | JF487858 | Brazil | 2006 |
| 6TI00A06 | JF487860 | Brazil | 2006 |
| 6TJ00A05 | JF487861 | Brazil | 2005 |
| 6TK00A05 | JF487863 | Brazil | 2005 |
| 6TL00A05 | JF487865 | Brazil | 2005 |
| 6TM00A05 | JF487866 | Brazil | 2005 |
| 6TN00A06 | JF487869 | Brazil | 2006 |
| 6TO00A06 | JF487870 | Brazil | 2006 |
| 6TR00A05 | JF487895 | Brazil | 2005 |
| 6TS00A05 | JF487896 | Brazil | 2005 |
| 6TT00A07 | JF487898 | Brazil | 2007 |
| 6TW00A06 | JF487901 | Brazil | 2006 |
| 6TX00A06 | JF487902 | Brazil | 2006 |
| 6TY00A07 | JF487903 | Brazil | 2007 |
| 6TZ00A06 | JF487904 | Brazil | 2006 |
| 6UD00B08 | JF773988 | Brazil | 2008 |
| 6UE00B08 | JF773998 | Brazil | 2008 |
| 6UF00B08 | JF774002 | Brazil | 2008 |
| 6UH00B08 | JF774011 | Brazil | 2008 |
| 6UI00B08 | JF774021 | Brazil | 2008 |
| 6UJ00B08 | JF774024 | Brazil | 2008 |
| 6UK00B08 | JF774029 | Brazil | 2008 |
| 6UM00B08 | JF774044 | Brazil | 2008 |
| 6UN00B08 | JF774052 | Brazil | 2008 |
| 6UO00A05 | JN010440 | Brazil | 2005 |
| 6UP00A05 | JN010441 | Brazil | 2005 |
| 6UQ00A05 | JN010450 | Brazil | 2005 |
| 6US02A05 | JN010459 | Brazil | 2005 |
| 6UT03A05 | JN010464 | Brazil | 2005 |
| 6UX0NA05 | JN010549 | Brazil | 2005 |
| 6WA0NA05 | JN010578 | Brazil | 2005 |
| 6WB0NA05 | JN010583 | Brazil | 2005 |
| 6WD0NA05 | JN010612 | Brazil | 2005 |
| 6WI0NA05 | JN010669 | Brazil | 2005 |
| 6WK0NA05 | JN010678 | Brazil | 2005 |
| 6WN0NA05 | JN010725 | Brazil | 2005 |
| 6WO0NA05 | JN010733 | Brazil | 2005 |
| 6WSNNN07 | JN634395 | Brazil | 2007 |
| 6WTNNN10 | JN634396 | Brazil | 2010 |
| 6WUNNN10 | JN634397 | Brazil | 2010 |
| 6WVNNN10 | JN634398 | Brazil | 2010 |
| 6WWNNN08 | JN634403 | Brazil | 2008 |
| 6WXNNN10 | JN634404 | Brazil | 2010 |
| 6WYNNN10 | JN634405 | Brazil | 2010 |
| 6XANNN05 | JN634453 | Brazil | 2005 |
| 6XBNNN08 | JN634454 | Brazil | 2008 |
| 6XCNNN09 | JN634455 | Brazil | 2009 |
| 6XDNNN09 | JN634456 | Brazil | 2009 |
| 6XENNN10 | JN634457 | Brazil | 2010 |
| 6XFNNN08 | JN634458 | Brazil | 2008 |
| 6XGNNN08 | JN634459 | Brazil | 2008 |
| 6XHNNN08 | JN634460 | Brazil | 2008 |
| 6XINNN09 | JN634461 | Brazil | 2009 |
| 6XJ20D07 | JN687657 | Brazil | 2007 |
| 6XK20D07 | JN687658 | Brazil | 2007 |
| 6XLNND02 | JN692431 | Brazil | 2002 |
| 6XMNND02 | JN692432 | Brazil | 2002 |
| 6XNNND02 | JN692433 | Brazil | 2002 |
| 6XONND02 | JN692435 | Brazil | 2002 |
| 6XQNND02 | JN692439 | Brazil | 2002 |
| 6XRNND02 | JN692440 | Brazil | 2002 |
| 6XSNND02 | JN692443 | Brazil | 2002 |
| 6XTNND02 | JN692444 | Brazil | 2002 |
| 6XUN1D03 | JN692445 | Brazil | 2003 |
| 6XVNND03 | JN692446 | Brazil | 2003 |
| 6XWNND03 | JN692447 | Brazil | 2003 |
| 6XXNND04 | JN692450 | Brazil | 2004 |
| 6XYNND04 | JN692451 | Brazil | 2004 |
| 6XZNND04 | JN692452 | Brazil | 2004 |
| 6YANND04 | JN692453 | Brazil | 2004 |
| 6YBNND04 | JN692455 | Brazil | 2004 |
| 6YCNND04 | JN692457 | Brazil | 2004 |
| 6YENND05 | JN692459 | Brazil | 2005 |
| 6YFN1D05 | JN692460 | Brazil | 2005 |
| 6YGNND05 | JN692461 | Brazil | 2005 |
| 6YHNND05 | JN692462 | Brazil | 2005 |
| 6YINND05 | JN692463 | Brazil | 2005 |
| 6YJNND05 | JN692464 | Brazil | 2005 |
| 6YKNND05 | JN692465 | Brazil | 2005 |
| 6YMNND05 | JN692467 | Brazil | 2005 |
| 6YNNND05 | JN692468 | Brazil | 2005 |
| 6YPNND02 | JN692470 | Brazil | 2002 |
| 6YQNND05 | JN692471 | Brazil | 2005 |
| 6YSNND05 | JN692473 | Brazil | 2005 |
| 6YTNND05 | JN692474 | Brazil | 2005 |
| 6YVNND05 | JN692476 | Brazil | 2005 |
| 6YWNND06 | JN692479 | Brazil | 2006 |
| 6YXNND06 | JN692480 | Brazil | 2006 |
| 6ZC002N | AF456612 | Argentina | N |
| 6ZD002N | AF456613 | Argentina | N |
| 6ZE002N | AF456614 | Argentina | N |
| 6ZF002N | AF456615 | Argentina | N |
| 6ZG002N | AF456616 | Argentina | N |
| 6ZH002N | AF456617 | Argentina | N |
| 6ZI002N | AF456618 | Argentina | N |
| 6ZJ002N | AF456619 | Argentina | N |
| 6ZK002N | AF456620 | Argentina | N |
| 6ZL002N | AF456621 | Argentina | N |
| 6ZM002N | AF456622 | Argentina | N |
| 6ZN002N | AF456623 | Argentina | N |
| 6ZO002N | AF456624 | Argentina | N |
| 6ZP002N | AF456625 | Argentina | N |
| 6ZQ002N | AF456626 | Argentina | N |
| 6ZR002N | AF456627 | Argentina | N |
| 6ZS002N | AF456628 | Argentina | N |
| 6ZT002N | AF456629 | Argentina | N |
| 6ZU002N | AF456630 | Argentina | N |
| 6ZV002N | AF456631 | Argentina | N |
| 6ZW002N | AF456632 | Argentina | N |
| 6ZX002N | AF456633 | Argentina | N |
| 6ZY002N | AF456634 | Argentina | N |
| 6ZZ002N | AF456635 | Argentina | N |
| 60A002N | AF456636 | Argentina | N |
| 60C002N | AF456638 | Argentina | N |
| 60D002N | AF456639 | Argentina | N |
| 60E002N | AF456640 | Argentina | N |
| 60F002N | AF456641 | Argentina | N |
| 60G002N | AF456642 | Argentina | N |
| 60H002N | AF456643 | Argentina | N |
| 60I002N | AF456644 | Argentina | N |
| 60J002N | AF456645 | Argentina | N |
| 60K002N | AF456646 | Argentina | N |
| 60L002N | AF456647 | Argentina | N |
| 60M002N | AF456648 | Argentina | N |
| 60N002N | AF456649 | Argentina | N |
| 60O002N | AF456650 | Argentina | N |
| 60P002N | AF456651 | Argentina | N |
| 60Q002N | AF456652 | Argentina | N |
| 60R002N | AF456653 | Argentina | N |
| 60S002N | AF456654 | Argentina | N |
| 60T002N | AF456655 | Argentina | N |
| 60U002N | AF456656 | Argentina | N |
| 60V002N | AF456657 | Argentina | N |
| 60W002N | AF456658 | Argentina | N |
| 60X002N | AF456659 | Argentina | N |
| 60Y002N | AF456660 | Argentina | N |
| 61A002N | AF456662 | Argentina | N |
| 61B002N | AF456663 | Argentina | N |
| 61C002N | AF456664 | Argentina | N |
| 61D002N | AF456665 | Argentina | N |
| 61E002N | AF456666 | Argentina | N |
| 61F002N | AF456667 | Argentina | N |
| 61G002N | AF456668 | Argentina | N |
| 61H002N | AF456669 | Argentina | N |
| 61I002N | AF456670 | Argentina | N |
| 61J002N | AF456671 | Argentina | N |
| 61K002N | AF456672 | Argentina | N |
| 61L002N | AF456673 | Argentina | N |
| 61M002N | AF456674 | Argentina | N |
| 61N002N | AF456675 | Argentina | N |
| 61O002N | AF456676 | Argentina | N |
| 61P002N | AF456677 | Argentina | N |
| 61Q002N | AF456678 | Argentina | N |
| 61R002N | AF456679 | Argentina | N |
| 61S002N | AF456680 | Argentina | N |
| 61T002N | AF456681 | Argentina | N |
| 61U002N | AF456682 | Argentina | N |
| 61V002N | AF456683 | Argentina | N |
| 61W002N | AF456684 | Argentina | N |
| 61X002N | AF456685 | Argentina | N |
| 61Y002N | AF456686 | Argentina | N |
| 61Z002N | AF456687 | Argentina | N |
| 62A002N | AF456688 | Argentina | N |
| 62B002N | AF456689 | Argentina | N |
| 62C002N | AF456690 | Argentina | N |
| 62D002N | AF456691 | Argentina | N |
| 62E002N | AF456692 | Argentina | N |
| 62F002N | AF456693 | Argentina | N |
| 62G002N | AF456694 | Argentina | N |
| 62H002N | AF456695 | Argentina | N |
| 62I002N | AF456696 | Argentina | N |
| 62J002N | AF456697 | Argentina | N |
| 62K002N | AF456698 | Argentina | N |
| 62L002N | AF456699 | Argentina | N |
| 62M002N | AF456700 | Argentina | N |
| 62N002N | AF456701 | Argentina | N |
| 62O002N | AF456702 | Argentina | N |
| 62P002N | AF456703 | Argentina | N |
| 62Q002N | AF456704 | Argentina | N |
| 62R002N | AF456705 | Argentina | N |
| 62S002N | AF456706 | Argentina | N |
| 62T002N | AF456707 | Argentina | N |
| 62U002N | AF456708 | Argentina | N |
| 62V002N | AF456709 | Argentina | N |
| 62W002N | AF456710 | Argentina | N |
| 62Y002N | AF456712 | Argentina | N |
| 62Z002N | AF456713 | Argentina | N |
| 63A002N | AF456714 | Argentina | N |
| 63B002N | AF456715 | Argentina | N |
| 63C002N | AF456716 | Argentina | N |
| 63D002N | AF456717 | Argentina | N |
| 63E002N | AF456718 | Argentina | N |
| 63F002N | AF456719 | Argentina | N |
| 63G002N | AF456720 | Argentina | N |
| 63H002N | AF456721 | Argentina | N |
| 63I002N | AF456722 | Argentina | N |
| 63J002N | AF456723 | Argentina | N |
| 63K002N | AF456724 | Argentina | N |
| 63L002N | AF456725 | Argentina | N |
| 63M002N | AF456726 | Argentina | N |
| 63N002N | AF456727 | Argentina | N |
| 63O002N | AF456728 | Argentina | N |
| 63P002N | AF456729 | Argentina | N |
| 63Q002N | AF456730 | Argentina | N |
| 63R002N | AF456731 | Argentina | N |
| 63S002N | AF456732 | Argentina | N |
| 63U002N | AF456734 | Argentina | N |
| 63V002N | AF456735 | Argentina | N |
| 63W002N | AF456736 | Argentina | N |
| 63X002N | AF456737 | Argentina | N |
| 63Y002N | AF456738 | Argentina | N |
| 63Z002N | AF456739 | Argentina | N |
| 64A002N | AF456740 | Argentina | N |
| 64B002N | AF456741 | Argentina | N |
| 64C002N | AF456743 | Argentina | N |
| 64D002N | AF456744 | Argentina | N |
| 64E002N | AF456745 | Argentina | N |
| 64F002N | AF456746 | Argentina | N |
| 64G002N | AF456747 | Argentina | N |
| 64H002N | AF456748 | Argentina | N |
| 64I002N | AF456749 | Argentina | N |
| 64J002N | AF456750 | Argentina | N |
| 64K002N | AF456751 | Argentina | N |
| 64L002N | AF456752 | Argentina | N |
| 64M002N | AF456753 | Argentina | N |
| 64N002N | AF456754 | Argentina | N |
| 64O002N | AF456755 | Argentina | N |
| 64Q002N | AF456757 | Argentina | N |
| 64R002N | AF456758 | Argentina | N |
| 64S002N | AF456759 | Argentina | N |
| 64U002N | AF456761 | Argentina | N |
| 64V002N | AF456762 | Argentina | N |
| 64W002N | AF456763 | Argentina | N |
| 64X002N | AF456764 | Argentina | N |
| 64Y1N2N | AF470802 | Argentina | N |
| 64Z1N2N | AF470803 | Argentina | N |
| 65A1N2N | AF470804 | Argentina | N |
| 65B1N2N | AF470805 | Argentina | N |
| 65C1N2N | AF470806 | Argentina | N |
| 65E1N2N | AF470808 | Argentina | N |
| 65F1N2N | AF470809 | Argentina | N |
| 65G1N2N | AF470811 | Argentina | N |
| 65H1N2N | AF470812 | Argentina | N |
| 65J1N2N | AF470814 | Argentina | N |
| 65K1N2N | AF470815 | Argentina | N |
| 65L1N2N | AF470816 | Argentina | N |
| 65M1N2N | AF470817 | Argentina | N |
| 65N1N2N | AF470818 | Argentina | N |
| 65O1N2N | AF470819 | Argentina | N |
| 65P1N2N | AF470820 | Argentina | N |
| 65S1N2N | AF470823 | Argentina | N |
| 65T1N2N | AF470824 | Argentina | N |
| 65U1N2N | AF470825 | Argentina | N |
| 65V1N2N | AF470889 | Argentina | N |
| 65X1N2N | AF470891 | Argentina | N |
| 65Y1N2N | AF470892 | Argentina | N |
| 65Z002N | AY211624 | Argentina | N |
| 66A002N | AY211625 | Argentina | N |
| 66B002N | AY211626 | Argentina | N |
| 66C002N | AY211649 | Argentina | N |
| 66E002N | AY211651 | Argentina | N |
| 66F002N | AY211652 | Argentina | N |
| 66G002N | AY211653 | Argentina | N |
| 66H002N | AY211654 | Argentina | N |
| 66I002N | AY211655 | Argentina | N |
| 66J002N | AY211656 | Argentina | N |
| 66KNN201 | AY365470 | Argentina | 2001 |
| 66LNN201 | AY365472 | Argentina | 2001 |
| 66MNN201 | AY365474 | Argentina | 2001 |
| 66NNN201 | AY365477 | Argentina | 2001 |
| 66ONN201 | AY365479 | Argentina | 2001 |
| 66PNN201 | AY365482 | Argentina | 2001 |
| 66QNN201 | AY365486 | Argentina | 2001 |
| 66RNN201 | AY365495 | Argentina | 2001 |
| 66SNN201 | AY365496 | Argentina | 2001 |
| 66TNN201 | AY365497 | Argentina | 2001 |
| 66UNN201 | AY365502 | Argentina | 2001 |
| 66WNN201 | AY365505 | Argentina | 2001 |
| 66XNN201 | AY365507 | Argentina | 2001 |
| 66ZNN201 | AY365510 | Argentina | 2001 |
| 67ANN201 | AY365513 | Argentina | 2001 |
| 67BNN201 | AY365515 | Argentina | 2001 |
| 67CNN201 | AY365517 | Argentina | 2001 |
| 67DNN201 | AY365518 | Argentina | 2001 |
| 67ENN201 | AY365519 | Argentina | 2001 |
| 67FNN201 | AY365520 | Argentina | 2001 |
| 67GNN201 | AY365521 | Argentina | 2001 |
| 67HNN201 | AY365522 | Argentina | 2001 |
| 67INN201 | AY365525 | Argentina | 2001 |
| 67JNN201 | AY365526 | Argentina | 2001 |
| 67KNN201 | AY365529 | Argentina | 2001 |
| 67LNN201 | AY365530 | Argentina | 2001 |
| 67NNN201 | AY365532 | Argentina | 2001 |
| 67ONN201 | AY365533 | Argentina | 2001 |
| 67PNN201 | AY365534 | Argentina | 2001 |
| 67QNN201 | AY365535 | Argentina | 2001 |
| 67RNN201 | AY365540 | Argentina | 2001 |
| 67SNN201 | AY365543 | Argentina | 2001 |
| 67TNN201 | AY365544 | Argentina | 2001 |
| 67UNN201 | AY365547 | Argentina | 2001 |
| 67VNN201 | AY365550 | Argentina | 2001 |
| 67WNN201 | AY365552 | Argentina | 2001 |
| 67XNN201 | AY365555 | Argentina | 2001 |
| 67YNN201 | AY365556 | Argentina | 2001 |
| 67ZNN201 | AY365557 | Argentina | 2001 |
| 68ANN201 | AY365559 | Argentina | 2001 |
| 68BNN201 | AY365560 | Argentina | 2001 |
| 68CNN201 | AY365561 | Argentina | 2001 |
| 68DNN201 | AY365562 | Argentina | 2001 |
| 68ENN201 | AY365564 | Argentina | 2001 |
| 68FNN201 | AY365565 | Argentina | 2001 |
| 68GNN201 | AY365567 | Argentina | 2001 |
| 68HNN201 | AY365568 | Argentina | 2001 |
| 68INN201 | AY365569 | Argentina | 2001 |
| 68JNN201 | AY365572 | Argentina | 2001 |
| 68KNN201 | AY365573 | Argentina | 2001 |
| 68LNN201 | AY365574 | Argentina | 2001 |
| 68MNN201 | AY365576 | Argentina | 2001 |
| 68NNN201 | AY365577 | Argentina | 2001 |
| 68ONN201 | AY365579 | Argentina | 2001 |
| 68PNN201 | AY365580 | Argentina | 2001 |
| 68QNN201 | AY365581 | Argentina | 2001 |
| 68RNN201 | AY365582 | Argentina | 2001 |
| 68SNN201 | AY365585 | Argentina | 2001 |
| 68TNN201 | AY365587 | Argentina | 2001 |
| 68UNN201 | AY365589 | Argentina | 2001 |
| 68VNN201 | AY365590 | Argentina | 2001 |
| 68WNN201 | AY365594 | Argentina | 2001 |
| 68XNN201 | AY365595 | Argentina | 2001 |
| 68YNN201 | AY365596 | Argentina | 2001 |
| 68ZNN201 | AY365597 | Argentina | 2001 |
| 69ANN201 | AY365598 | Argentina | 2001 |
| 69BNN201 | AY365599 | Argentina | 2001 |
| 69CNN201 | AY365601 | Argentina | 2001 |
| 69DNN201 | AY365602 | Argentina | 2001 |
| 69ENN201 | AY365603 | Argentina | 2001 |
| 69FNN201 | AY365605 | Argentina | 2001 |
| 69GNN201 | AY365606 | Argentina | 2001 |
| 69INN201 | AY365608 | Argentina | 2001 |
| 69JNN201 | AY365610 | Argentina | 2001 |
| 69KNN201 | AY365611 | Argentina | 2001 |
| 69LNN201 | AY365612 | Argentina | 2001 |
| 69MNN201 | AY365614 | Argentina | 2001 |
| 69NNN201 | AY365615 | Argentina | 2001 |
| 69PNN201 | AY365617 | Argentina | 2001 |
| 69QNN201 | AY365618 | Argentina | 2001 |
| 69RNN201 | AY365621 | Argentina | 2001 |
| 69SNN201 | AY365624 | Argentina | 2001 |
| 69TNN201 | AY365626 | Argentina | 2001 |
| 69UNN201 | AY365631 | Argentina | 2001 |
| 69VNN201 | AY365633 | Argentina | 2001 |
| 69WNN201 | AY365634 | Argentina | 2001 |
| 69XNN201 | AY365635 | Argentina | 2001 |
| 69ZNN201 | AY365637 | Argentina | 2001 |
| 7AANN201 | AY365642 | Argentina | 2001 |
| 7ABNN201 | AY365644 | Argentina | 2001 |
| 7ACNN201 | AY365646 | Argentina | 2001 |
| 7ADNN201 | AY365647 | Argentina | 2001 |
| 7AENN201 | AY365648 | Argentina | 2001 |
| 7AFNN201 | AY365650 | Argentina | 2001 |
| 7AGNN201 | AY365651 | Argentina | 2001 |
| 7AHNN201 | AY365652 | Argentina | 2001 |
| 7AINN201 | AY365654 | Argentina | 2001 |
| 7AJNN201 | AY365656 | Argentina | 2001 |
| 7AKNN201 | AY365657 | Argentina | 2001 |
| 7AMNN201 | AY365662 | Argentina | 2001 |
| 7ANNN201 | AY365666 | Argentina | 2001 |
| 7AONN201 | AY365667 | Argentina | 2001 |
| 7APNN201 | AY365668 | Argentina | 2001 |
| 7AQNN201 | AY365674 | Argentina | 2001 |
| 7ARNN201 | AY365679 | Argentina | 2001 |
| 7ASNN201 | AY365680 | Argentina | 2001 |
| 7ATNN201 | AY365685 | Argentina | 2001 |
| 7AUNN201 | AY365688 | Argentina | 2001 |
| 7AVNN201 | AY365689 | Argentina | 2001 |
| 7AWNN201 | AY365690 | Argentina | 2001 |
| 7AYNN201 | AY365693 | Argentina | 2001 |
| 7AZNN201 | AY365695 | Argentina | 2001 |
| 7BANN201 | AY365696 | Argentina | 2001 |
| 7BBNN201 | AY365702 | Argentina | 2001 |
| 7BCNN201 | AY365704 | Argentina | 2001 |
| 7BDNN201 | AY365705 | Argentina | 2001 |
| 7BENN201 | AY365708 | Argentina | 2001 |
| 7BFNN201 | AY365709 | Argentina | 2001 |
| 7BGNN201 | AY365710 | Argentina | 2001 |
| 7BHNN201 | AY365713 | Argentina | 2001 |
| 7BINN201 | AY365717 | Argentina | 2001 |
| 7BJNN201 | AY365718 | Argentina | 2001 |
| 7BKNN201 | AY365722 | Argentina | 2001 |
| 7BLNN201 | AY365726 | Argentina | 2001 |
| 7BMNN201 | AY365731 | Argentina | 2001 |
| 7BNNN201 | AY365733 | Argentina | 2001 |
| 7BONN201 | AY365735 | Argentina | 2001 |
| 7BPNN201 | AY365737 | Argentina | 2001 |
| 7BQNN201 | AY365739 | Argentina | 2001 |
| 7BRNN201 | AY365747 | Argentina | 2001 |
| 7BSNN201 | AY365749 | Argentina | 2001 |
| 7BTNN201 | AY365750 | Argentina | 2001 |
| 7BUNN201 | AY365752 | Argentina | 2001 |
| 7BVNN201 | AY365753 | Argentina | 2001 |
| 7BWNN201 | AY365755 | Argentina | 2001 |
| 7BXNN201 | AY365757 | Argentina | 2001 |
| 7BYNN201 | AY365764 | Argentina | 2001 |
| 7BZNN201 | AY365765 | Argentina | 2001 |
| 7CANN201 | AY365766 | Argentina | 2001 |
| 7CBNN201 | AY365769 | Argentina | 2001 |
| 7CCNN201 | AY365771 | Argentina | 2001 |
| 7CDNN201 | AY365772 | Argentina | 2001 |
| 7CENN201 | AY365773 | Argentina | 2001 |
| 7CGNN201 | AY365779 | Argentina | 2001 |
| 7CHNN201 | AY365780 | Argentina | 2001 |
| 7CINN201 | AY365781 | Argentina | 2001 |
| 7CJNN201 | AY365782 | Argentina | 2001 |
| 7CKNN201 | AY365784 | Argentina | 2001 |
| 7CLNN201 | AY365785 | Argentina | 2001 |
| 7CMNN201 | AY365788 | Argentina | 2001 |
| 7CNNN201 | AY365791 | Argentina | 2001 |
| 7CONN201 | AY365792 | Argentina | 2001 |
| 7CPNN201 | AY365793 | Argentina | 2001 |
| 7CQNN201 | AY365794 | Argentina | 2001 |
| 7CRNN201 | AY365795 | Argentina | 2001 |
| 7CSNN201 | AY365797 | Argentina | 2001 |
| 7CTNN201 | AY365798 | Argentina | 2001 |
| 7CUNN201 | AY365799 | Argentina | 2001 |
| 7CVNN201 | AY365807 | Argentina | 2001 |
| 7CWNN201 | AY365808 | Argentina | 2001 |
| 7CXNN201 | AY365814 | Argentina | 2001 |
| 7CYNN201 | AY365815 | Argentina | 2001 |
| 7CZNN201 | AY365816 | Argentina | 2001 |
| 7DANN201 | AY365817 | Argentina | 2001 |
| 7DBNN201 | AY365821 | Argentina | 2001 |
| 7DCNN201 | AY365822 | Argentina | 2001 |
| 7DDNN201 | AY365825 | Argentina | 2001 |
| 7DENN201 | AY365830 | Argentina | 2001 |
| 7DFNN201 | AY365831 | Argentina | 2001 |
| 7DGNN201 | AY365842 | Argentina | 2001 |
| 7DHNN201 | AY365843 | Argentina | 2001 |
| 7DINN201 | AY365844 | Argentina | 2001 |
| 7DJNN201 | AY365845 | Argentina | 2001 |
| 7DKNN201 | AY365846 | Argentina | 2001 |
| 7DLNN201 | AY365847 | Argentina | 2001 |
| 7DMNN201 | AY365848 | Argentina | 2001 |
| 7DNNN201 | AY365849 | Argentina | 2001 |
| 7DONN201 | AY365853 | Argentina | 2001 |
| 7DPNN201 | AY365855 | Argentina | 2001 |
| 7DQNN201 | AY365858 | Argentina | 2001 |
| 7DRNN201 | AY365860 | Argentina | 2001 |
| 7DSNN201 | AY365863 | Argentina | 2001 |
| 7DTNN201 | AY365866 | Argentina | 2001 |
| 7DUNN201 | AY365867 | Argentina | 2001 |
| 7DVNN201 | AY365868 | Argentina | 2001 |
| 7DWNN201 | AY365869 | Argentina | 2001 |
| 7DXNN201 | AY365870 | Argentina | 2001 |
| 7DYNN201 | AY365872 | Argentina | 2001 |
| 7DZNN201 | AY365873 | Argentina | 2001 |
| 7EANN201 | AY365874 | Argentina | 2001 |
| 7EBNN201 | AY365876 | Argentina | 2001 |
| 7ECNN201 | AY365877 | Argentina | 2001 |
| 7EDNN201 | AY365878 | Argentina | 2001 |
| 7EENN201 | AY365879 | Argentina | 2001 |
| 7EFNN201 | AY365882 | Argentina | 2001 |
| 7EGNN201 | AY365883 | Argentina | 2001 |
| 7EHNN201 | AY365886 | Argentina | 2001 |
| 7EINN201 | AY365887 | Argentina | 2001 |
| 7EKNN201 | AY365889 | Argentina | 2001 |
| 7ELNN201 | AY365890 | Argentina | 2001 |
| 7EMNN201 | AY365892 | Argentina | 2001 |
| 7ENNN201 | AY365893 | Argentina | 2001 |
| 7EONN201 | AY365894 | Argentina | 2001 |
| 7EPNN201 | AY365897 | Argentina | 2001 |
| 7EQNN201 | AY365898 | Argentina | 2001 |
| 7ERNN201 | AY365899 | Argentina | 2001 |
| 7ETNN201 | AY365902 | Argentina | 2001 |
| 7EUNN201 | AY365903 | Argentina | 2001 |
| 7EVNN201 | AY365904 | Argentina | 2001 |
| 7EWNN201 | AY365907 | Argentina | 2001 |
| 7EXNN201 | AY365908 | Argentina | 2001 |
| 7EYNN201 | AY365910 | Argentina | 2001 |
| 7FANN201 | AY365915 | Argentina | 2001 |
| 7FBNN201 | AY365918 | Argentina | 2001 |
| 7FCNN201 | AY365919 | Argentina | 2001 |
| 7FDNN201 | AY365920 | Argentina | 2001 |
| 7FENN201 | AY365921 | Argentina | 2001 |
| 7FFNN201 | AY365922 | Argentina | 2001 |
| 7FGNN201 | AY365923 | Argentina | 2001 |
| 7FHNN201 | AY365929 | Argentina | 2001 |
| 7FINN201 | AY365930 | Argentina | 2001 |
| 7FJNN201 | AY365932 | Argentina | 2001 |
| 7FKNN201 | AY365938 | Argentina | 2001 |
| 7FLNN201 | AY365940 | Argentina | 2001 |
| 7FMNN201 | AY365941 | Argentina | 2001 |
| 7FNNN201 | AY365943 | Argentina | 2001 |
| 7FONN201 | AY365945 | Argentina | 2001 |
| 7FPNN201 | AY365946 | Argentina | 2001 |
| 7FQNN201 | AY365948 | Argentina | 2001 |
| 7FRNN201 | AY365950 | Argentina | 2001 |
| 7FSNN201 | AY365951 | Argentina | 2001 |
| 7FTNN201 | AY365954 | Argentina | 2001 |
| 7FVNN201 | AY365956 | Argentina | 2001 |
| 7FWNN201 | AY365957 | Argentina | 2001 |
| 7FXNN201 | AY365962 | Argentina | 2001 |
| 7FYNN201 | AY365964 | Argentina | 2001 |
| 7FZNN201 | AY365970 | Argentina | 2001 |
| 7GANN201 | AY365971 | Argentina | 2001 |
| 7GBNN201 | AY365972 | Argentina | 2001 |
| 7GCNN201 | AY365973 | Argentina | 2001 |
| 7GDNN201 | AY365975 | Argentina | 2001 |
| 7GENN201 | AY365979 | Argentina | 2001 |
| 7GFNN201 | AY365980 | Argentina | 2001 |
| 7GGNN201 | AY365982 | Argentina | 2001 |
| 7GHNN201 | AY365984 | Argentina | 2001 |
| 7GINN201 | AY365985 | Argentina | 2001 |
| 7GJNN201 | AY365986 | Argentina | 2001 |
| 7GKNN201 | AY365989 | Argentina | 2001 |
| 7GLNN201 | AY365995 | Argentina | 2001 |
| 7GMNN201 | AY365996 | Argentina | 2001 |
| 7GNNN201 | AY365997 | Argentina | 2001 |
| 7GONN201 | AY365998 | Argentina | 2001 |
| 7GPNN201 | AY365999 | Argentina | 2001 |
| 7GQNN201 | AY366002 | Argentina | 2001 |
| 7GRNN201 | AY366004 | Argentina | 2001 |
| 7GSNN201 | AY366008 | Argentina | 2001 |
| 7GTNN201 | AY366010 | Argentina | 2001 |
| 7GUNN201 | AY366011 | Argentina | 2001 |
| 7GVNN201 | AY366016 | Argentina | 2001 |
| 7GWNN201 | AY366017 | Argentina | 2001 |
| 7GXNN201 | AY366024 | Argentina | 2001 |
| 7GYNN201 | AY366025 | Argentina | 2001 |
| 7GZNN201 | AY366026 | Argentina | 2001 |
| 7HANN201 | AY366029 | Argentina | 2001 |
| 7HBNN201 | AY366035 | Argentina | 2001 |
| 7HCNN201 | AY366036 | Argentina | 2001 |
| 7HDNN201 | AY366037 | Argentina | 2001 |
| 7HENN201 | AY366038 | Argentina | 2001 |
| 7HFNN201 | AY366039 | Argentina | 2001 |
| 7HGNN201 | AY366042 | Argentina | 2001 |
| 7HHNN201 | AY366044 | Argentina | 2001 |
| 7HINN201 | AY366045 | Argentina | 2001 |
| 7HJNN201 | AY366046 | Argentina | 2001 |
| 7HKNN201 | AY366049 | Argentina | 2001 |
| 7HLNN201 | AY366051 | Argentina | 2001 |
| 7HMNN201 | AY366054 | Argentina | 2001 |
| 7HNNN201 | AY366055 | Argentina | 2001 |
| 7HO002N | AY493358 | Argentina | N |
| 7HP002N | AY493359 | Argentina | N |
| 7HQ002N | AY493360 | Argentina | N |
| 7HR002N | AY493363 | Argentina | N |
| 7HS002N | AY493364 | Argentina | N |
| 7HT002N | AY493366 | Argentina | N |
| 7HU002N | AY493367 | Argentina | N |
| 7HV002N | AY493368 | Argentina | N |
| 7HW002N | AY493371 | Argentina | N |
| 7HYNN204 | DQ518496 | Argentina | 2004 |
| 7HZNN204 | DQ518498 | Argentina | 2004 |
| 7IANN204 | DQ518500 | Argentina | 2004 |
| 7IBNN204 | DQ518502 | Argentina | 2004 |
| 7ICNN204 | DQ518512 | Argentina | 2004 |
| 7IDNN204 | DQ518514 | Argentina | 2004 |
| 7IENN204 | DQ877743 | Argentina | 2004 |
| 7IFNN203 | DQ877744 | Argentina | 2003 |
| 7IG052N | DQ995539 | Argentina | N |
| 7IH052N | DQ995548 | Argentina | N |
| 7II052N | DQ995550 | Argentina | N |
| 7IJ052N | DQ995551 | Argentina | N |
| 7IK052N | DQ995553 | Argentina | N |
| 7IL052N | DQ995555 | Argentina | N |
| 7IM052N | DQ995557 | Argentina | N |
| 7IN052N | DQ995569 | Argentina | N |
| 7IO052N | DQ995570 | Argentina | N |
| 7IP052N | DQ995579 | Argentina | N |
| 7IQ052N | DQ995583 | Argentina | N |
| 7IR052N | DQ995586 | Argentina | N |
| 7IS052N | DQ995587 | Argentina | N |
| 7IT052N | DQ995588 | Argentina | N |
| 7IUNN203 | EF120074 | Argentina | 2003 |
| 7IVNN203 | EF120075 | Argentina | 2003 |
| 7IWNN203 | EF120080 | Argentina | 2003 |
| 7IXNN203 | EF120084 | Argentina | 2003 |
| 7IYNN203 | EF120087 | Argentina | 2003 |
| 7IZNN203 | EF120088 | Argentina | 2003 |
| 7JANN203 | EF120095 | Argentina | 2003 |
| 7JBNN203 | EF120097 | Argentina | 2003 |
| 7JCNN203 | EF120098 | Argentina | 2003 |
| 7JDNN203 | EF120102 | Argentina | 2003 |
| 7JENN203 | EF120103 | Argentina | 2003 |
| 7JFNN203 | EF120105 | Argentina | 2003 |
| 7JGNN204 | EF120112 | Argentina | 2004 |
| 7JHNN203 | EF120115 | Argentina | 2003 |
| 7JINN203 | EF120116 | Argentina | 2003 |
| 7JJNN203 | EF120124 | Argentina | 2003 |
| 7JKNN203 | EF120125 | Argentina | 2003 |
| 7JLNN203 | EF120126 | Argentina | 2003 |
| 7JMNN203 | EF120133 | Argentina | 2003 |
| 7JNNN203 | EF120135 | Argentina | 2003 |
| 7JPNN203 | EF120150 | Argentina | 2003 |
| 7JQNN203 | EF120153 | Argentina | 2003 |
| 7JRNN203 | EF120155 | Argentina | 2003 |
| 7JSNN203 | EF120157 | Argentina | 2003 |
| 7JTNN203 | EF120158 | Argentina | 2003 |
| 7JUNN203 | EF120162 | Argentina | 2003 |
| 7JVNN203 | EF120165 | Argentina | 2003 |
| 7JWNN203 | EF120168 | Argentina | 2003 |
| 7JXNN203 | EF120171 | Argentina | 2003 |
| 7JYNN203 | EF120175 | Argentina | 2003 |
| 7JZNN203 | EF120179 | Argentina | 2003 |
| 7KANN203 | EF120181 | Argentina | 2003 |
| 7KBNN203 | EF120183 | Argentina | 2003 |
| 7KCNN203 | EF120184 | Argentina | 2003 |
| 7KDNN203 | EF120186 | Argentina | 2003 |
| 7KENN203 | EF120187 | Argentina | 2003 |
| 7KFNN203 | EF120190 | Argentina | 2003 |
| 7KHNN203 | EF120194 | Argentina | 2003 |
| 7KJNN203 | EF120197 | Argentina | 2003 |
| 7KKNN203 | EF120200 | Argentina | 2003 |
| 7KLNN203 | EF120201 | Argentina | 2003 |
| 7KMNN203 | EF120202 | Argentina | 2003 |
| 7KNNN203 | EF120203 | Argentina | 2003 |
| 7KONN203 | EF120206 | Argentina | 2003 |
| 7KPNN203 | EF120207 | Argentina | 2003 |
| 7KQNN203 | EF120209 | Argentina | 2003 |
| 7KSNN203 | EF120212 | Argentina | 2003 |
| 7KTNN203 | EF120215 | Argentina | 2003 |
| 7KUNN203 | EF120219 | Argentina | 2003 |
| 7KWNN203 | EF120226 | Argentina | 2003 |
| 7KXNN203 | EF120229 | Argentina | 2003 |
| 7KYNN203 | EF120230 | Argentina | 2003 |
| 7KZNN203 | EF120234 | Argentina | 2003 |
| 7LBNN203 | EF120237 | Argentina | 2003 |
| 7LENN203 | EF120243 | Argentina | 2003 |
| 7LFNN203 | EF120245 | Argentina | 2003 |
| 7LGNN203 | EF120246 | Argentina | 2003 |
| 7LHNN203 | EF120247 | Argentina | 2003 |
| 7LINN203 | EF120248 | Argentina | 2003 |
| 7LJNN203 | EF120249 | Argentina | 2003 |
| 7LKNN203 | EF120250 | Argentina | 2003 |
| 7LLNN203 | EF120259 | Argentina | 2003 |
| 7LNNN203 | EF120263 | Argentina | 2003 |
| 7LONN203 | EF120270 | Argentina | 2003 |
| 7LPNN203 | EF120271 | Argentina | 2003 |
| 7LQNN203 | EF120273 | Argentina | 2003 |
| 7LRNN203 | EF120274 | Argentina | 2003 |
| 7LSNN203 | EF120276 | Argentina | 2003 |
| 7LTNN203 | EF120277 | Argentina | 2003 |
| 7LUNN203 | EF120281 | Argentina | 2003 |
| 7LVNN203 | EF120282 | Argentina | 2003 |
| 7LWNN203 | EF120286 | Argentina | 2003 |
| 7LXNN203 | EF120287 | Argentina | 2003 |
| 7LYNN203 | EF120288 | Argentina | 2003 |
| 7LZNN203 | EF120289 | Argentina | 2003 |
| 7MANN203 | EF120291 | Argentina | 2003 |
| 7MBNN203 | EF120294 | Argentina | 2003 |
| 7MCNN203 | EF120295 | Argentina | 2003 |
| 7MDNN203 | EF120296 | Argentina | 2003 |
| 7MENN203 | EF120297 | Argentina | 2003 |
| 7MFNN203 | EF120298 | Argentina | 2003 |
| 7MGNN203 | EF120299 | Argentina | 2003 |
| 7MHNN203 | EF120302 | Argentina | 2003 |
| 7MINN203 | EF120305 | Argentina | 2003 |
| 7MJNN203 | EF120306 | Argentina | 2003 |
| 7MKNN203 | EF120307 | Argentina | 2003 |
| 7MLNN203 | EF120310 | Argentina | 2003 |
| 7MMNN203 | EF120312 | Argentina | 2003 |
| 7MNNN203 | EF120314 | Argentina | 2003 |
| 7MONN203 | EF120315 | Argentina | 2003 |
| 7MPNN203 | EF120316 | Argentina | 2003 |
| 7MRNN203 | EF120318 | Argentina | 2003 |
| 7MSNN203 | EF120319 | Argentina | 2003 |
| 7MTNN203 | EF120320 | Argentina | 2003 |
| 7MUNN203 | EF120321 | Argentina | 2003 |
| 7MWNN203 | EF120323 | Argentina | 2003 |
| 7MXNN203 | EF120324 | Argentina | 2003 |
| 7MYNN203 | EF120326 | Argentina | 2003 |
| 7MZNN203 | EF120329 | Argentina | 2003 |
| 7NANN203 | eF120330 | Argentina | 2003 |
| 7NCNN203 | eF120332 | Argentina | 2003 |
| 7NDNN203 | eF120333 | Argentina | 2003 |
| 7NENN203 | eF120334 | Argentina | 2003 |
| 7NFNN203 | eF120335 | Argentina | 2003 |
| 7NGNN204 | eF120339 | Argentina | 2004 |
| 7NHNN203 | eF120341 | Argentina | 2003 |
| 7NKNN203 | EF120346 | Argentina | 2003 |
| 7NLNN203 | EF120347 | Argentina | 2003 |
| 7NMNN203 | EF120351 | Argentina | 2003 |
| 7NNNN203 | EF120354 | Argentina | 2003 |
| 7NONN203 | EF120356 | Argentina | 2003 |
| 7NP002N | EU046511 | Argentina | N |
| 7NQ002N | EU046512 | Argentina | N |
| 7NR002N | EU046513 | Argentina | N |
| 7NS002N | EU046514 | Argentina | N |
| 7NT002N | EU046515 | Argentina | N |
| 7OB002N | EU046523 | Argentina | N |
| 7OC002N | EU046524 | Argentina | N |
| 7OD002N | EU046525 | Argentina | N |
| 7OE002N | EU046526 | Argentina | N |
| 7OF002N | EU046527 | Argentina | N |
| 7OI002N | EU046530 | Argentina | N |
| 7OJ002N | EU046531 | Argentina | N |
| 7OK002N | EU046532 | Argentina | N |
| 7OL002N | EU046533 | Argentina | N |
| 7OM002N | EU046534 | Argentina | N |
| 7ON002N | EU046535 | Argentina | N |
| 7OO002N | EU046536 | Argentina | N |
| 7OP002N | EU046537 | Argentina | N |
| 7OQ002N | EU046538 | Argentina | N |
| 7OR002N | EU046539 | Argentina | N |
| 7OS002N | EU046540 | Argentina | N |
| 7OT002N | EU046541 | Argentina | N |
| 7OV002N | EU046551 | Argentina | N |
| 7OW002N | EU707944 | Argentina | N |
| 7OX03205 | EU707945 | Argentina | 2005 |
| 7OY03205 | EU707946 | Argentina | 2005 |
| 7OZ03205 | EU707947 | Argentina | 2005 |
| 7PA03205 | EU707948 | Argentina | 2005 |
| 7PB03205 | EU707949 | Argentina | 2005 |
| 7PC03205 | EU707950 | Argentina | 2005 |
| 7PD03205 | EU707951 | Argentina | 2005 |
| 7PE03205 | EU707952 | Argentina | 2005 |
| 7PF03205 | EU707953 | Argentina | 2005 |
| 7PG03205 | EU707954 | Argentina | 2005 |
| 7PH03205 | EU707955 | Argentina | 2005 |
| 7PI03205 | EU707957 | Argentina | 2005 |
| 7PJ03204 | EU707971 | Argentina | 2004 |
| 7PK05206 | FJ525814 | Argentina | 2006 |
| 7PL05207 | FJ525822 | Argentina | 2007 |
| 7PM05207 | FJ525844 | Argentina | 2007 |
| 7PN05207 | FJ525849 | Argentina | 2007 |
| 7PO05207 | FJ525851 | Argentina | 2007 |
| 7PP05208 | FJ525868 | Argentina | 2008 |
| 7PQ14207 | JN206625 | Argentina | 2007 |
| 7PR11204 | JN206626 | Argentina | 2004 |
| 7PSN0202 | JN669432 | Argentina | 2002 |
| 7PUN0203 | JN669436 | Argentina | 2003 |
| 7PVN0203 | JN669441 | Argentina | 2003 |
| 7PWN0203 | JN669443 | Argentina | 2003 |
| 7PXN0203 | JN669445 | Argentina | 2003 |
| 7PYN0203 | JN669446 | Argentina | 2003 |
| 7PZN0203 | JN669448 | Argentina | 2003 |
| 7QAN0203 | JN669451 | Argentina | 2003 |
| 7QBN0203 | JN669453 | Argentina | 2003 |
| 7QCN0203 | JN669460 | Argentina | 2003 |
| 7QDN0203 | JN669461 | Argentina | 2003 |
| 7QEN0203 | JN669462 | Argentina | 2003 |
| 7QFN0203 | JN669463 | Argentina | 2003 |
| 7QHN0203 | JN669465 | Argentina | 2003 |
| 7QIN0203 | JN669466 | Argentina | 2003 |
| 7QJN0203 | JN669467 | Argentina | 2003 |
| 7QKN0203 | JN669472 | Argentina | 2003 |
| 7QLN0203 | JN669475 | Argentina | 2003 |
| 7QMN0203 | JN669476 | Argentina | 2003 |
| 7QNN0203 | JN669477 | Argentina | 2003 |
| 7QON0203 | JN669478 | Argentina | 2003 |
| 7QPN0203 | JN669479 | Argentina | 2003 |
| 7QQN0203 | JN669480 | Argentina | 2003 |
| 7QRN0203 | JN669481 | Argentina | 2003 |
| 7QSN0203 | JN669488 | Argentina | 2003 |
| 7QTN0203 | JN669489 | Argentina | 2003 |
| 7QVN0203 | JN669491 | Argentina | 2003 |
| 7QWN0203 | JN669492 | Argentina | 2003 |
| 7QXN0203 | JN669495 | Argentina | 2003 |
| 7QZN0203 | JN669497 | Argentina | 2003 |
| 7RAN0203 | JN669500 | Argentina | 2003 |
| 7RBN0203 | JN669502 | Argentina | 2003 |
| 7RCN0203 | JN669503 | Argentina | 2003 |
| 7RDN0203 | JN669505 | Argentina | 2003 |
| 7REN0203 | JN669506 | Argentina | 2003 |
| 7RFN0203 | JN669509 | Argentina | 2003 |
| 7RGN0203 | JN669511 | Argentina | 2003 |
| 7RHN0203 | JN669512 | Argentina | 2003 |
| 7RJN0203 | JN669514 | Argentina | 2003 |
| 7RKN0203 | JN669515 | Argentina | 2003 |
| 7RLN0203 | JN669518 | Argentina | 2003 |
| 7RON0203 | JN669525 | Argentina | 2003 |
| 7RPN0203 | JN669528 | Argentina | 2003 |
| 7RQN0203 | JN669529 | Argentina | 2003 |
| 7RRN0203 | JN669530 | Argentina | 2003 |
| 7RTN0203 | JN669534 | Argentina | 2003 |
| 7RUN0203 | JN669536 | Argentina | 2003 |
| 7RVN0203 | JN669539 | Argentina | 2003 |
| 7RWN0203 | JN669541 | Argentina | 2003 |
| 7RXN0203 | JN669542 | Argentina | 2003 |
| 7RYN0203 | JN669543 | Argentina | 2003 |
| 7RZN0203 | JN669544 | Argentina | 2003 |
| 7SAN0203 | JN669546 | Argentina | 2003 |
| 7SBN0203 | JN669547 | Argentina | 2003 |
| 7SCN0203 | JN669548 | Argentina | 2003 |
| 7SDN0203 | JN669549 | Argentina | 2003 |
| 7SEN0203 | JN669550 | Argentina | 2003 |
| 7SFN0203 | JN669551 | Argentina | 2003 |
| 7SGN0203 | JN669553 | Argentina | 2003 |
| 7SHN0203 | JN669554 | Argentina | 2003 |
| 7SIN0203 | JN669560 | Argentina | 2003 |
| 7SJN0203 | JN669564 | Argentina | 2003 |
| 7SKN0203 | JN669565 | Argentina | 2003 |
| 7SMN0203 | JN669568 | Argentina | 2003 |
| 7SNN0203 | JN669569 | Argentina | 2003 |
| 7SON0203 | JN669574 | Argentina | 2003 |
| 7SPN0203 | JN669575 | Argentina | 2003 |
| 7SQN0203 | JN669576 | Argentina | 2003 |
| 7SRN0203 | JN669577 | Argentina | 2003 |
| 7SSN0203 | JN669578 | Argentina | 2003 |
| 7STN0203 | JN669581 | Argentina | 2003 |
| 7SUN0203 | JN669582 | Argentina | 2003 |
| 7SVN0203 | JN669584 | Argentina | 2003 |
| 7SWN0203 | JN669586 | Argentina | 2003 |
| 7SXN0203 | JN669588 | Argentina | 2003 |
| 7SYN0203 | JN669592 | Argentina | 2003 |
| 7SZN0203 | JN669593 | Argentina | 2003 |
| 7TAN0203 | JN669594 | Argentina | 2003 |
| 7TBN0203 | JN669595 | Argentina | 2003 |
| 7TCN0203 | JN669596 | Argentina | 2003 |
| 7TDN0203 | JN669597 | Argentina | 2003 |
| 7TEN0203 | JN669598 | Argentina | 2003 |
| 7TFN0203 | JN669600 | Argentina | 2003 |
| 7TGN0203 | JN669603 | Argentina | 2003 |
| 7THN0203 | JN669604 | Argentina | 2003 |
| 7TIN0203 | JN669605 | Argentina | 2003 |
| 7TJN0203 | JN669606 | Argentina | 2003 |
| 7TKN0203 | JN669609 | Argentina | 2003 |
| 7TLN0203 | JN669610 | Argentina | 2003 |
| 7TMN0203 | JN669611 | Argentina | 2003 |
| 7TNN0203 | JN669612 | Argentina | 2003 |
| 7TON0204 | JN669616 | Argentina | 2004 |
| 7TPN0204 | JN669617 | Argentina | 2004 |
| 7TQN0204 | JN669619 | Argentina | 2004 |
| 7TRN0204 | JN669621 | Argentina | 2004 |
| 7TSN0204 | JN669623 | Argentina | 2004 |
| 7TTN0204 | JN669624 | Argentina | 2004 |
| 7TUN0204 | JN669626 | Argentina | 2004 |
| 7TVN0204 | JN669627 | Argentina | 2004 |
| 7TWN0204 | JN669628 | Argentina | 2004 |
| 7TXN0204 | JN669632 | Argentina | 2004 |
| 7TZN0204 | JN669637 | Argentina | 2004 |
| 7UAN0204 | JN669638 | Argentina | 2004 |
| 7UBN0204 | JN669639 | Argentina | 2004 |
| 7UCN0204 | JN669643 | Argentina | 2004 |
| 7UDN0204 | JN669645 | Argentina | 2004 |
| 7UEN0204 | JN669649 | Argentina | 2004 |
| 7UFN0204 | JN669650 | Argentina | 2004 |
| 7UGN0204 | JN669653 | Argentina | 2004 |
| 7UHN0204 | JN669657 | Argentina | 2004 |
| 7UIN0204 | JN669658 | Argentina | 2004 |
| 7UJN0204 | JN669659 | Argentina | 2004 |
| 7UMN0204 | JN669666 | Argentina | 2004 |
| 7UNN0204 | JN669667 | Argentina | 2004 |
| 7UON0204 | JN669669 | Argentina | 2004 |
| 7UPN0204 | JN669670 | Argentina | 2004 |
| 7UQN0204 | JN669671 | Argentina | 2004 |
| 7USN0204 | jN669674 | Argentina | 2004 |
| 7UTN0204 | jN669675 | Argentina | 2004 |
| 7UUN0204 | JN669676 | Argentina | 2004 |
| 7UWN0204 | JN669678 | Argentina | 2004 |
| 7UXN0204 | JN669680 | Argentina | 2004 |
| 7UYN0204 | JN669683 | Argentina | 2004 |
| 7UZN0204 | JN669686 | Argentina | 2004 |
| 7WBN0204 | JN669695 | Argentina | 2004 |
| 7WCN0204 | JN669702 | Argentina | 2004 |
| 7WDN0204 | JN669704 | Argentina | 2004 |
| 7WEN0204 | JN669705 | Argentina | 2004 |
| 7WFN0204 | JN669706 | Argentina | 2004 |
| 7WHN0204 | JN669709 | Argentina | 2004 |
| 7WIN0204 | JN669710 | Argentina | 2004 |
| 7WJN0204 | JN669711 | Argentina | 2004 |
| 7WKN0204 | JN669712 | Argentina | 2004 |
| 7WLN0204 | JN669713 | Argentina | 2004 |
| 7WMN0204 | JN669717 | Argentina | 2004 |
| 7WNN0204 | JN669722 | Argentina | 2004 |
| 7WON0204 | JN669725 | Argentina | 2004 |
| 7WPN0204 | JN669726 | Argentina | 2004 |
| 7WQN0204 | JN669729 | Argentina | 2004 |
| 7WRN0204 | JN669732 | Argentina | 2004 |
| 7WSN0204 | JN669736 | Argentina | 2004 |
| 7WTN0204 | JN669740 | Argentina | 2004 |
| 7WUN0204 | JN669742 | Argentina | 2004 |
| 7WVN0204 | JN669744 | Argentina | 2004 |
| 7WWN0204 | JN669746 | Argentina | 2004 |
| 7WXN0204 | jN669747 | Argentina | 2004 |
| 7WYN0204 | JN669748 | Argentina | 2004 |
| 7WZN0204 | JN669749 | Argentina | 2004 |
| 7XAN0204 | JN669751 | Argentina | 2004 |
| 7XBN0204 | JN669754 | Argentina | 2004 |
| 7XCN02N | JN669755 | Argentina | N |
| 7XDN0204 | JN669759 | Argentina | 2004 |
| 7XEN0204 | JN669760 | Argentina | 2004 |
| 7XFN0204 | JN669762 | Argentina | 2004 |
| 7XGN0204 | JN669763 | Argentina | 2004 |
| 7XHN0204 | JN669764 | Argentina | 2004 |
| 7XIN0204 | JN669768 | Argentina | 2004 |
| 7XJN0204 | JN669769 | Argentina | 2004 |
| 7XKN0204 | JN669770 | Argentina | 2004 |
| 7XLN0204 | JN669771 | Argentina | 2004 |
| 7XMN0204 | JN669773 | Argentina | 2004 |
| 7XNN0204 | JN669774 | Argentina | 2004 |
| 7XON0204 | JN669777 | Argentina | 2004 |
| 7XPN0204 | JN669779 | Argentina | 2004 |
| 7XQN0204 | JN669781 | Argentina | 2004 |
| 7XRN0204 | JN669782 | Argentina | 2004 |
| 7XSN0204 | JN669787 | Argentina | 2004 |
| 7XTN0204 | JN669788 | Argentina | 2004 |
| 7XUN0204 | JN669790 | Argentina | 2004 |
| 7XVN0204 | JN669791 | Argentina | 2004 |
| 7XWN0204 | JN669792 | Argentina | 2004 |
| 7XXN0207 | JN669794 | Argentina | 2007 |
| 7XYN0204 | JN669795 | Argentina | 2004 |
| 7XZN0204 | JN669798 | Argentina | 2004 |
| 7YBN0204 | JN669800 | Argentina | 2004 |
| 7YEN0204 | JN669804 | Argentina | 2004 |
| 7YGN0204 | JN669810 | Argentina | 2004 |
| 7YHN0204 | JN669812 | Argentina | 2004 |
| 7YIN0204 | JN669814 | Argentina | 2004 |
| 7YJN0204 | JN669815 | Argentina | 2004 |
| 7YKN0204 | JN669818 | Argentina | 2004 |
| 7YLN0204 | JN669819 | Argentina | 2004 |
| 7YMN02N | JN669821 | Argentina | N |
| 7YON0204 | JN669823 | Argentina | 2004 |
| 7YQN0204 | JN669829 | Argentina | 2004 |
| 7YRN0204 | JN669830 | Argentina | 2004 |
| 7YSN0204 | JN669831 | Argentina | 2004 |
| 7YTN0204 | JN669832 | Argentina | 2004 |
| 7YUN0204 | JN669833 | Argentina | 2004 |
| 7YWN0204 | JN669836 | Argentina | 2004 |
| 7YXN0204 | JN669837 | Argentina | 2004 |
| 7YYN0204 | JN669838 | Argentina | 2004 |
| 7YZN0204 | JN669840 | Argentina | 2004 |
| 7ZAN0204 | JN669841 | Argentina | 2004 |
| 7ZBN0204 | JN669844 | Argentina | 2004 |
| 7ZCN0204 | JN669845 | Argentina | 2004 |
| 7ZDN0204 | JN669847 | Argentina | 2004 |
| 7ZEN0204 | JN669849 | Argentina | 2004 |
| 7ZFN0204 | JN669850 | Argentina | 2004 |
| 7ZHN0204 | JN669853 | Argentina | 2004 |
| 7ZJN0204 | JN669855 | Argentina | 2004 |
| 7ZLN0204 | JN669859 | Argentina | 2004 |
| 7ZMN0204 | JN669860 | Argentina | 2004 |
| 7ZNN0204 | JN669861 | Argentina | 2004 |
| 7ZON0204 | JN669862 | Argentina | 2004 |
| 7ZPN0204 | JN669868 | Argentina | 2004 |
| 7ZQN0204 | JN669870 | Argentina | 2004 |
| 7ZRN0204 | JN669871 | Argentina | 2004 |
| 7ZSN0204 | JN669872 | Argentina | 2004 |
| 7ZTN0204 | JN669874 | Argentina | 2004 |
| 7ZUN0204 | JN669875 | Argentina | 2004 |
| 7ZVN0204 | JN669876 | Argentina | 2004 |
| 7ZWN0204 | JN669877 | Argentina | 2004 |
| 7ZXN0204 | JN669884 | Argentina | 2004 |
| 7ZYN0204 | JN669886 | Argentina | 2004 |
| 70AN0204 | JN669889 | Argentina | 2004 |
| 70BN0204 | JN669891 | Argentina | 2004 |
| 70CN0204 | JN669894 | Argentina | 2004 |
| 70DN0204 | JN669897 | Argentina | 2004 |
| 70EN0204 | JN669898 | Argentina | 2004 |
| 70FN0204 | JN669900 | Argentina | 2004 |
| 70GN0204 | JN669902 | Argentina | 2004 |
| 70HN0204 | JN669904 | Argentina | 2004 |
| 70JN0204 | JN669908 | Argentina | 2004 |
| 70KN0204 | JN669909 | Argentina | 2004 |
| 70LN0204 | JN669913 | Argentina | 2004 |
| 70MN0204 | JN669915 | Argentina | 2004 |
| 70NN0204 | JN669917 | Argentina | 2004 |
| 70ON0204 | JN669918 | Argentina | 2004 |
| 70PN02N | JN669920 | Argentina | N |
| 70QN0204 | JN669921 | Argentina | 2004 |
| 70RN0204 | JN669923 | Argentina | 2004 |
| 70SN0204 | JN669926 | Argentina | 2004 |
| 70TN0204 | JN669929 | Argentina | 2004 |
| 70UN0204 | JN669931 | Argentina | 2004 |
| 70VN0204 | JN669932 | Argentina | 2004 |
| 70WN0204 | JN669933 | Argentina | 2004 |
| 70XN0204 | JN669934 | Argentina | 2004 |
| 70YN0204 | JN669937 | Argentina | 2004 |
| 70ZN0204 | JN669938 | Argentina | 2004 |
| 71BN0204 | JN669942 | Argentina | 2004 |
| 71CN0204 | JN669945 | Argentina | 2004 |
| 71DN0204 | JN669950 | Argentina | 2004 |
| 71FN0204 | JN669952 | Argentina | 2004 |
| 71GN0204 | JN669958 | Argentina | 2004 |
| 71HN0204 | JN669962 | Argentina | 2004 |
| 71IN0204 | JN669963 | Argentina | 2004 |
| 71JN0204 | JN669964 | Argentina | 2004 |
| 71LN0204 | JN669967 | Argentina | 2004 |
| 71MN0204 | JN669970 | Argentina | 2004 |
| 71NN0204 | JN669971 | Argentina | 2004 |
| 71ON0204 | JN669978 | Argentina | 2004 |
| 71PN0204 | JN669979 | Argentina | 2004 |
| 71QN0204 | JN669982 | Argentina | 2004 |
| 71RN0204 | JN669985 | Argentina | 2004 |
| 71SN0204 | JN669986 | Argentina | 2004 |
| 71TN0204 | JN669989 | Argentina | 2004 |
| 71UN0204 | JN669990 | Argentina | 2004 |
| 71VN0204 | JN669993 | Argentina | 2004 |
| 71WN0204 | JN669994 | Argentina | 2004 |
| 71XN0204 | JN669995 | Argentina | 2004 |
| 71YN0204 | JN669997 | Argentina | 2004 |
| 72AN0204 | JN670002 | Argentina | 2004 |
| 72BN0204 | JN670005 | Argentina | 2004 |
| 72CN0204 | JN670006 | Argentina | 2004 |
| 72EN0204 | JN670019 | Argentina | 2004 |
| 72HN0204 | JN670023 | Argentina | 2004 |
| 72IN0204 | JN670031 | Argentina | 2004 |
| 72JN0204 | JN670032 | Argentina | 2004 |
| 72KN0204 | JN670033 | Argentina | 2004 |
| 72LN0204 | JN670034 | Argentina | 2004 |
| 72MN0204 | JN670035 | Argentina | 2004 |
| 72NN0204 | JN670036 | Argentina | 2004 |
| 72ON0204 | JN670037 | Argentina | 2004 |
| 72PN0204 | JN670038 | Argentina | 2004 |
| 72QN0205 | JN670042 | Argentina | 2005 |
| 72RN0205 | JN670043 | Argentina | 2005 |
| 72SN0205 | JN670044 | Argentina | 2005 |
| 72TN0205 | JN670046 | Argentina | 2005 |
| 72UN0205 | JN670047 | Argentina | 2005 |
| 72VN0205 | JN670048 | Argentina | 2005 |
| 72WN0205 | JN670051 | Argentina | 2005 |
| 72ZN0205 | JN670054 | Argentina | 2005 |
| 73AN0205 | JN670055 | Argentina | 2005 |
| 73BN0205 | JN670056 | Argentina | 2005 |
| 73EN0205 | JN670059 | Argentina | 2005 |
| 73FN0205 | JN670062 | Argentina | 2005 |
| 73GN0205 | JN670063 | Argentina | 2005 |
| 73HN0205 | JN670064 | Argentina | 2005 |
| 73IN0205 | JN670066 | Argentina | 2005 |
| 73JN0205 | JN670067 | Argentina | 2005 |
| 73KN0205 | JN670071 | Argentina | 2005 |
| 73LN0205 | JN670072 | Argentina | 2005 |
| 73MN0205 | JN670076 | Argentina | 2005 |
| 73NN0205 | JN670084 | Argentina | 2005 |
| 73ON0205 | J670085 | Argentina | 2005 |
| 73PN0205 | JN670086 | Argentina | 2005 |
| 73QN0205 | JN670087 | Argentina | 2005 |
| 73RN0205 | JN670095 | Argentina | 2005 |
| 73SN0205 | JN670096 | Argentina | 2005 |
| 73TN0205 | JN670099 | Argentina | 2005 |
| 73UN0205 | JN670101 | Argentina | 2005 |
| 73VN0205 | JN670103 | Argentina | 2005 |
| 73WN0205 | JN670104 | Argentina | 2005 |
| 73XN0205 | JN670107 | Argentina | 2005 |
| 73YN0205 | JN670108 | Argentina | 2005 |
| 73ZN0205 | JN670110 | Argentina | 2005 |
| 74AN0205 | JN670111 | Argentina | 2005 |
| 74BN0205 | JN670112 | Argentina | 2005 |
| 74CN0205 | JN670113 | Argentina | 2005 |
| 74DN0205 | JN670115 | Argentina | 2005 |
| 74EN0205 | JN670116 | Argentina | 2005 |
| 74FN0205 | JN670118 | Argentina | 2005 |
| 74GN0205 | JN670120 | Argentina | 2005 |
| 74HN0205 | JN670123 | Argentina | 2005 |
| 74IN0205 | JN670125 | Argentina | 2005 |
| 74KN0205 | JN670131 | Argentina | 2005 |
| 74LN0205 | JN670133 | Argentina | 2005 |
| 74MN0205 | JN670134 | Argentina | 2005 |
| 74NN0205 | JN670135 | Argentina | 2005 |
| 74ON0205 | JN670136 | Argentina | 2005 |
| 74PN0205 | JN670140 | Argentina | 2005 |
| 74QN0205 | JN670144 | Argentina | 2005 |
| 74RN0205 | JN670146 | Argentina | 2005 |
| 74TN0205 | JN670153 | Argentina | 2005 |
| 74UN0205 | JN670154 | Argentina | 2005 |
| 74WN0205 | JN670158 | Argentina | 2005 |
| 74XN0205 | JN670163 | Argentina | 2005 |
| 74YN0205 | JN670164 | Argentina | 2005 |
| 75AN0205 | JN670169 | Argentina | 2005 |
| 75CN0205 | JN670171 | Argentina | 2005 |
| 75DN0205 | JN670172 | Argentina | 2005 |
| 75EN0205 | JN670175 | Argentina | 2005 |
| 75FN0205 | JN670176 | Argentina | 2005 |
| 75GN0205 | JN670177 | Argentina | 2005 |
| 75HN0205 | JN670181 | Argentina | 2005 |
| 75IN0205 | JN670183 | Argentina | 2005 |
| 75JN0205 | JN670185 | Argentina | 2005 |
| 75KN0205 | JN670186 | Argentina | 2005 |
| 75LN0205 | JN670187 | Argentina | 2005 |
| 75MN0205 | JN670189 | Argentina | 2005 |
| 75NN0205 | JN670191 | Argentina | 2005 |
| 75ON0205 | JN670192 | Argentina | 2005 |
| 75PN0205 | JN670193 | Argentina | 2005 |
| 75RN0205 | JN670197 | Argentina | 2005 |
| 75TN0205 | JN670202 | Argentina | 2005 |
| 75UN0205 | JN670205 | Argentina | 2005 |
| 75VN0205 | JN670206 | Argentina | 2005 |
| 75WN0205 | JN670208 | Argentina | 2005 |
| 75XN0205 | JN670209 | Argentina | 2005 |
| 75YN0205 | JN670210 | Argentina | 2005 |
| 75ZN0205 | JN670214 | Argentina | 2005 |
| 76AN0205 | JN670215 | Argentina | 2005 |
| 76BN0205 | JN670216 | Argentina | 2005 |
| 76CN0205 | JN670217 | Argentina | 2005 |
| 76DN0205 | JN670218 | Argentina | 2005 |
| 76EN0205 | JN670225 | Argentina | 2005 |
| 76FN0205 | JN670226 | Argentina | 2005 |
| 76GN0205 | JN670227 | Argentina | 2005 |
| 76HN0205 | JN670228 | Argentina | 2005 |
| 76JN0205 | JN670234 | Argentina | 2005 |
| 76KN0205 | JN670241 | Argentina | 2005 |
| 76LN0205 | JN670245 | Argentina | 2005 |
| 76MN0205 | JN670248 | Argentina | 2005 |
| 76NN0205 | JN670249 | Argentina | 2005 |
| 76ON0205 | JN670252 | Argentina | 2005 |
| 76QN0205 | JN670260 | Argentina | 2005 |
| 76RN0205 | JN670261 | Argentina | 2005 |
| 76SN0205 | JN670262 | Argentina | 2005 |
| 76TN0205 | JN670264 | Argentina | 2005 |
| 76UN0205 | JN670265 | Argentina | 2005 |
| 76VN0205 | JN670268 | Argentina | 2005 |
| 76WN0205 | JN670272 | Argentina | 2005 |
| 76XN0205 | JN670273 | Argentina | 2005 |
| 76YN02N | JN670275 | Argentina | N |
| 76ZN0205 | JN670277 | Argentina | 2005 |
| 77AN0205 | JN670280 | Argentina | 2005 |
| 77BN0205 | JN670281 | Argentina | 2005 |
| 77CN0205 | JN670287 | Argentina | 2005 |
| 77DN0205 | JN670291 | Argentina | 2005 |
| 77EN0205 | JN670295 | Argentina | 2005 |
| 77FN0205 | JN670298 | Argentina | 2005 |
| 77GN0205 | JN670303 | Argentina | 2005 |
| 77HN0205 | JN670304 | Argentina | 2005 |
| 77KN0205 | JN670318 | Argentina | 2005 |
| 77LN0205 | JN670319 | Argentina | 2005 |
| 77MN0205 | JN670322 | Argentina | 2005 |
| 77NN0205 | JN670323 | Argentina | 2005 |
| 77ON0205 | JN670325 | Argentina | 2005 |
| 77PN0205 | jN670326 | Argentina | 2005 |
| 77QN0205 | jN670328 | Argentina | 2005 |
| 77RN0205 | JN670329 | Argentina | 2005 |
| 77SN0205 | JN670331 | Argentina | 2005 |
| 77TN0205 | JN670333 | Argentina | 2005 |
| 77UN0205 | JN670334 | Argentina | 2005 |
| 77VN0205 | JN670337 | Argentina | 2005 |
| 77WN0205 | jN670338 | Argentina | 2005 |
| 77XN0205 | jN670340 | Argentina | 2005 |
| 77YN02N | JN670345 | Argentina | N |
| 77ZN0205 | JN670346 | Argentina | 2005 |
| 78AN0205 | JN670347 | Argentina | 2005 |
| 78BN0205 | JN670348 | Argentina | 2005 |
| 78CN0205 | JN670350 | Argentina | 2005 |
| 78DN0205 | JN670351 | Argentina | 2005 |
| 78EN0205 | JN670352 | Argentina | 2005 |
| 78FN0205 | JN670356 | Argentina | 2005 |
| 78GN0205 | JN670358 | Argentina | 2005 |
| 78HN0205 | JN670362 | Argentina | 2005 |
| 78IN0205 | JN670366 | Argentina | 2005 |
| 78JN0205 | JN670367 | Argentina | 2005 |
| 78KN0205 | JN670369 | Argentina | 2005 |
| 78LN0205 | JN670370 | Argentina | 2005 |
| 78MN0205 | JN670371 | Argentina | 2005 |
| 78NN0205 | JN670376 | Argentina | 2005 |
| 78ON0205 | JN670377 | Argentina | 2005 |
| 78PN0205 | JN670379 | Argentina | 2005 |
| 78QN0205 | JN670385 | Argentina | 2005 |
| 78RN0205 | JN670387 | Argentina | 2005 |
| 78SN0205 | JN670388 | Argentina | 2005 |
| 78TN0205 | JN670392 | Argentina | 2005 |
| 78VN0205 | JN670396 | Argentina | 2005 |
| 78XN0205 | JN670400 | Argentina | 2005 |
| 78ZN0205 | JN670403 | Argentina | 2005 |
| 79AN0205 | JN670407 | Argentina | 2005 |
| 79BN0205 | JN670408 | Argentina | 2005 |
| 79CN0205 | JN670411 | Argentina | 2005 |
| 79DN0205 | JN670412 | Argentina | 2005 |
| 79EN0205 | JN670413 | Argentina | 2005 |
| 79FN0205 | JN670417 | Argentina | 2005 |
| 79GN02N | JN670423 | Argentina | N |
| 79HN02N | JN670424 | Argentina | N |
| 79IN0205 | JN670426 | Argentina | 2005 |
| 79JN02N | JN670431 | Argentina | N |
| 79KN0205 | JN670433 | Argentina | 2005 |
| 79MN0205 | JN670440 | Argentina | 2005 |
| 79NN0205 | JN670441 | Argentina | 2005 |
| 79ON0205 | JN670446 | Argentina | 2005 |
| 79PN0205 | JN670447 | Argentina | 2005 |
| 79QN0205 | JN670448 | Argentina | 2005 |
| 79RN0205 | JN670449 | Argentina | 2005 |
| 79TN0205 | JN670452 | Argentina | 2005 |
| 79UN0205 | JN670453 | Argentina | 2005 |
| 79VN0205 | JN670457 | Argentina | 2005 |
| 79WN0205 | JN670458 | Argentina | 2005 |
| 79XN02N | JN670460 | Argentina | N |
| 79YN0205 | JN670465 | Argentina | 2005 |
| 79ZN0205 | JN670466 | Argentina | 2005 |
| 8AAN0205 | JN670467 | Argentina | 2005 |
| 8ABN0205 | JN670474 | Argentina | 2005 |
| 8ADN0205 | JN670481 | Argentina | 2005 |
| 8AFN0205 | JN670485 | Argentina | 2005 |
| 8AGN0205 | JN670489 | Argentina | 2005 |
| 8AHN0205 | JN670490 | Argentina | 2005 |
| 8AIN0205 | JN670493 | Argentina | 2005 |
| 8AJN0205 | JN670494 | Argentina | 2005 |
| 8AKN0205 | JN670496 | Argentina | 2005 |
| 8ALN0205 | JN670497 | Argentina | 2005 |
| 8AON0205 | JN670508 | Argentina | 2005 |
| 8APN0205 | JN670519 | Argentina | 2005 |
| 8AQN0205 | JN670520 | Argentina | 2005 |
| 8ARN0205 | JN670522 | Argentina | 2005 |
| 8ASN0205 | JN670527 | Argentina | 2005 |
| 8AUN0205 | JN670532 | Argentina | 2005 |
| 8AVN0205 | JN670534 | Argentina | 2005 |
| 8AWN0205 | JN670535 | Argentina | 2005 |
| 8AYN0205 | JN670539 | Argentina | 2005 |
| 8AZN0205 | JN670542 | Argentina | 2005 |
| 8BAN0205 | JN670543 | Argentina | 2005 |
| 8BCN0205 | JN670547 | Argentina | 2005 |
| 8BDN0205 | JN670550 | Argentina | 2005 |
| 8BFN0205 | JN670554 | Argentina | 2005 |
| 8BGN0205 | JN670555 | Argentina | 2005 |
| 8BIN0205 | JN670558 | Argentina | 2005 |
| 8BJN0205 | JN670560 | Argentina | 2005 |
| 8BKN0205 | JN670561 | Argentina | 2005 |
| 8BMN0205 | JN670567 | Argentina | 2005 |
| 8BON0205 | JN670569 | Argentina | 2005 |
| 8BPN0205 | JN670572 | Argentina | 2005 |
| 8BQN0205 | JN670573 | Argentina | 2005 |
| 8BRN0205 | JN670574 | Argentina | 2005 |
| 8BSN0205 | JN670575 | Argentina | 2005 |
| 8BTN0205 | JN670577 | Argentina | 2005 |
| 8BUN0205 | JN670578 | Argentina | 2005 |
| 8BVN0205 | JN670579 | Argentina | 2005 |
| 8BWN0205 | JN670580 | Argentina | 2005 |
| 8BYN0205 | JN670584 | Argentina | 2005 |
| 8BZN0205 | JN670586 | Argentina | 2005 |
| 8CAN0205 | JN670588 | Argentina | 2005 |
| 8CDN0205 | JN670596 | Argentina | 2005 |
| 8CEN0205 | JN670598 | Argentina | 2005 |
| 8CFN0205 | JN670600 | Argentina | 2005 |
| 8CGN0205 | JN670602 | Argentina | 2005 |
| 8CHN0205 | JN670604 | Argentina | 2005 |
| 8CIN0205 | JN670607 | Argentina | 2005 |
| 8CJN0205 | JN670610 | Argentina | 2005 |
| 8CKN0205 | JN670613 | Argentina | 2005 |
| 8CLN0205 | JN670616 | Argentina | 2005 |
| 8CNN0205 | JN670619 | Argentina | 2005 |
| 8CON0205 | JN670620 | Argentina | 2005 |
| 8CPN0205 | JN670622 | Argentina | 2005 |
| 8CQN0206 | JN670625 | Argentina | 2006 |
| 8CRN0206 | JN670626 | Argentina | 2006 |
| 8CSN0206 | JN670628 | Argentina | 2006 |
| 8CUN0206 | jN670631 | Argentina | 2006 |
| 8CVN0206 | JN670633 | Argentina | 2006 |
| 8CWN0206 | jN670634 | Argentina | 2006 |
| 8CXN0206 | jN670636 | Argentina | 2006 |
| 8CYN0206 | jN670640 | Argentina | 2006 |
| 8CZN0206 | jN670641 | Argentina | 2006 |
| 8DCN0206 | jN670646 | Argentina | 2006 |
| 8DDN0206 | JN670651 | Argentina | 2006 |
| 8DHN0206 | jN670657 | Argentina | 2006 |
| 8DJN0206 | JN670663 | Argentina | 2006 |
| 8DKN0206 | JN670664 | Argentina | 2006 |
| 8DLN0206 | JN670669 | Argentina | 2006 |
| 8DMN0206 | JN670674 | Argentina | 2006 |
| 8DNN0206 | JN670675 | Argentina | 2006 |
| 8DON0206 | JN670677 | Argentina | 2006 |
| 8DPN0206 | JN670681 | Argentina | 2006 |
| 8DQN0206 | JN670686 | Argentina | 2006 |
| 8DRN0206 | JN670687 | Argentina | 2006 |
| 8DSN0206 | JN670688 | Argentina | 2006 |
| 8DTN0206 | JN670690 | Argentina | 2006 |
| 8DUN0206 | JN670692 | Argentina | 2006 |
| 8DVN0206 | JN670695 | Argentina | 2006 |
| 8DWN0206 | JN670696 | Argentina | 2006 |
| 8DXN0206 | JN670698 | Argentina | 2006 |
| 8DYN0206 | JN670699 | Argentina | 2006 |
| 8DZN0206 | JN670700 | Argentina | 2006 |
| 8EBN0206 | JN670704 | Argentina | 2006 |
| 8ECN0206 | JN670707 | Argentina | 2006 |
| 8EDN0206 | JN670708 | Argentina | 2006 |
| 8EEN0206 | JN670709 | Argentina | 2006 |
| 8EFN0206 | JN670713 | Argentina | 2006 |
| 8EHN0206 | JN670716 | Argentina | 2006 |
| 8EIN0206 | JN670718 | Argentina | 2006 |
| 8EJN0206 | JN670719 | Argentina | 2006 |
| 8ELN0206 | JN670726 | Argentina | 2006 |
| 8EMN0206 | JN670730 | Argentina | 2006 |
| 8EON0206 | JN670740 | Argentina | 2006 |
| 8EQN0206 | JN670745 | Argentina | 2006 |
| 8ERN0206 | jN670749 | Argentina | 2006 |
| 8ESN0206 | JN670752 | Argentina | 2006 |
| 8ETN0206 | JN670756 | Argentina | 2006 |
| 8EUN0206 | JN670757 | Argentina | 2006 |
| 8EVN0206 | JN670759 | Argentina | 2006 |
| 8EWN0206 | JN670760 | Argentina | 2006 |
| 8EYN0206 | jN670774 | Argentina | 2006 |
| 8EZN0206 | JN670775 | Argentina | 2006 |
| 8FAN0206 | JN670782 | Argentina | 2006 |
| 8FBN0206 | JN670788 | Argentina | 2006 |
| 8FCN0206 | JN670790 | Argentina | 2006 |
| 8FDN0206 | JN670791 | Argentina | 2006 |
| 8FEN0206 | JN670793 | Argentina | 2006 |
| 8FFN0206 | JN670799 | Argentina | 2006 |
| 8FGN0206 | JN670805 | Argentina | 2006 |
| 8FHN0206 | JN670806 | Argentina | 2006 |
| 8FIN0206 | JN670810 | Argentina | 2006 |
| 8FJN0206 | JN670812 | Argentina | 2006 |
| 8FKN0206 | JN670813 | Argentina | 2006 |
| 8FLN0206 | JN670814 | Argentina | 2006 |
| 8FMN0206 | JN670815 | Argentina | 2006 |
| 8FNN0206 | JN670818 | Argentina | 2006 |
| 8FON0206 | JN670820 | Argentina | 2006 |
| 8FPN0206 | JN670821 | Argentina | 2006 |
| 8FQN0206 | JN670822 | Argentina | 2006 |
| 8FSN0206 | JN670832 | Argentina | 2006 |
| 8FTN0206 | JN670833 | Argentina | 2006 |
| 8FUN0206 | JN670840 | Argentina | 2006 |
| 8FVN0206 | JN670841 | Argentina | 2006 |
| 8FWN0206 | JN670843 | Argentina | 2006 |
| 8FXN0206 | JN670845 | Argentina | 2006 |
| 8FZN0206 | JN670850 | Argentina | 2006 |
| 8GAN0206 | JN670852 | Argentina | 2006 |
| 8GBN0206 | JN670854 | Argentina | 2006 |
| 8GCN0206 | JN670855 | Argentina | 2006 |
| 8GDN0206 | JN670858 | Argentina | 2006 |
| 8GEN0206 | JN670860 | Argentina | 2006 |
| 8GFN0206 | JN670861 | Argentina | 2006 |
| 8GGN0206 | JN670867 | Argentina | 2006 |
| 8GIN0206 | JN670871 | Argentina | 2006 |
| 8GJN0206 | JN670874 | Argentina | 2006 |
| 8GKN0206 | JN670878 | Argentina | 2006 |
| 8GLN0206 | JN670882 | Argentina | 2006 |
| 8GMN0206 | JN670884 | Argentina | 2006 |
| 8GNN0206 | JN670885 | Argentina | 2006 |
| 8GON0206 | JN670888 | Argentina | 2006 |
| 8GPN0206 | JN670889 | Argentina | 2006 |
| 8GQN0206 | JN670891 | Argentina | 2006 |
| 8GRN0206 | JN670892 | Argentina | 2006 |
| 8GSN0206 | JN670896 | Argentina | 2006 |
| 8GTN0206 | JN670897 | Argentina | 2006 |
| 8GVN0206 | JN670900 | Argentina | 2006 |
| 8GWN0206 | JN670902 | Argentina | 2006 |
| 8GXN0206 | JN670903 | Argentina | 2006 |
| 8GZN0206 | JN670908 | Argentina | 2006 |
| 8HAN0206 | JN670909 | Argentina | 2006 |
| 8HCN0206 | JN670915 | Argentina | 2006 |
| 8HDN0206 | JN670916 | Argentina | 2006 |
| 8HEN0206 | JN670917 | Argentina | 2006 |
| 8HFN0206 | JN670921 | Argentina | 2006 |
| 8HGN0206 | JN670925 | Argentina | 2006 |
| 8HHN0206 | JN670927 | Argentina | 2006 |
| 8HIN0206 | JN670928 | Argentina | 2006 |
| 8HKN0206 | JN670933 | Argentina | 2006 |
| 8HLN0206 | JN670936 | Argentina | 2006 |
| 8HMN0206 | JN670937 | Argentina | 2006 |
| 8HON0206 | JN670940 | Argentina | 2006 |
| 8HPN0206 | JN670941 | Argentina | 2006 |
| 8HRN0206 | JN670943 | Argentina | 2006 |
| 8HSN0206 | JN670944 | Argentina | 2006 |
| 8HTN0206 | JN670948 | Argentina | 2006 |
| 8HVN0206 | JN670951 | Argentina | 2006 |
| 8HWN0206 | JN670952 | Argentina | 2006 |
| 8HXN0206 | JN670954 | Argentina | 2006 |
| 8HZN0206 | JN670958 | Argentina | 2006 |
| 8IAN0206 | JN670959 | Argentina | 2006 |
| 8IBN0206 | JN670960 | Argentina | 2006 |
| 8ICN0206 | JN670962 | Argentina | 2006 |
| 8IDN0206 | JN670963 | Argentina | 2006 |
| 8IEN0206 | JN670966 | Argentina | 2006 |
| 8IFN0206 | JN670969 | Argentina | 2006 |
| 8IIN0206 | JN670979 | Argentina | 2006 |
| 8IJN0206 | JN670981 | Argentina | 2006 |
| 8IKN0206 | JN670982 | Argentina | 2006 |
| 8ILN0206 | JN670983 | Argentina | 2006 |
| 8INN0206 | JN670985 | Argentina | 2006 |
| 8ION0206 | JN670989 | Argentina | 2006 |
| 8IPN0206 | JN670991 | Argentina | 2006 |
| 8IQN0206 | JN670992 | Argentina | 2006 |
| 8IRN0206 | JN670993 | Argentina | 2006 |
| 8ISN0206 | JN670998 | Argentina | 2006 |
| 8ITN0206 | JN671003 | Argentina | 2006 |
| 8IUN0206 | JN671008 | Argentina | 2006 |
| 8IWN0206 | JN671013 | Argentina | 2006 |
| 8IXN0206 | JN671017 | Argentina | 2006 |
| 8IZN0206 | JN671024 | Argentina | 2006 |
| 8JBN0206 | JN671028 | Argentina | 2006 |
| 8JCN0206 | JN671031 | Argentina | 2006 |
| 8JDN0206 | JN671033 | Argentina | 2006 |
| 8JEN0206 | JN671036 | Argentina | 2006 |
| 8JFN0206 | JN671037 | Argentina | 2006 |
| 8JGN0206 | JN671040 | Argentina | 2006 |
| 8JHN0206 | JN671041 | Argentina | 2006 |
| 8JIN0206 | JN671046 | Argentina | 2006 |
| 8JJN0206 | JN671047 | Argentina | 2006 |
| 8JKN0206 | JN671048 | Argentina | 2006 |
| 8JLN0206 | JN671049 | Argentina | 2006 |
| 8JMN0206 | JN671051 | Argentina | 2006 |
| 8JNN0206 | JN671058 | Argentina | 2006 |
| 8JON0206 | JN671059 | Argentina | 2006 |
| 8JPN0206 | JN671060 | Argentina | 2006 |
| 8JQN0206 | JN671061 | Argentina | 2006 |
| 8JRN0206 | JN671062 | Argentina | 2006 |
| 8JSN0206 | JN671066 | Argentina | 2006 |
| 8JUN0206 | JN671069 | Argentina | 2006 |
| 8JVN0206 | JN671070 | Argentina | 2006 |
| 8JWN0206 | JN671071 | Argentina | 2006 |
| 8JXN0206 | JN671072 | Argentina | 2006 |
| 8JZN0206 | JN671075 | Argentina | 2006 |
| 8KAN0206 | JN671078 | Argentina | 2006 |
| 8KBN0206 | JN671080 | Argentina | 2006 |
| 8KCN0206 | JN671081 | Argentina | 2006 |
| 8KDN0206 | JN671083 | Argentina | 2006 |
| 8KEN0206 | JN671084 | Argentina | 2006 |
| 8KGN0206 | JN671087 | Argentina | 2006 |
| 8KHN0206 | JN671089 | Argentina | 2006 |
| 8KJN0206 | JN671091 | Argentina | 2006 |
| 8KKN0206 | JN671092 | Argentina | 2006 |
| 8KLN0206 | JN671094 | Argentina | 2006 |
| 8KNN0206 | JN671098 | Argentina | 2006 |
| 8KON0206 | JN671099 | Argentina | 2006 |
| 8KQN0206 | JN671106 | Argentina | 2006 |
| 8KRN0206 | JN671112 | Argentina | 2006 |
| 8KTN0206 | JN671117 | Argentina | 2006 |
| 8KUN0206 | JN671119 | Argentina | 2006 |
| 8KVN0206 | JN671122 | Argentina | 2006 |
| 8KWN0206 | JN671123 | Argentina | 2006 |
| 8KXN0206 | JN671124 | Argentina | 2006 |
| 8KYN0206 | JN671125 | Argentina | 2006 |
| 8KZN0206 | JN671126 | Argentina | 2006 |
| 8LAN0206 | JN671127 | Argentina | 2006 |
| 8LBN0206 | JN671132 | Argentina | 2006 |
| 8LCN0206 | JN671137 | Argentina | 2006 |
| 8LDN0206 | JN671141 | Argentina | 2006 |
| 8LEN0206 | JN671142 | Argentina | 2006 |
| 8LHN0206 | JN671151 | Argentina | 2006 |
| 8LIN0206 | JN671153 | Argentina | 2006 |
| 8LJN0206 | JN671154 | Argentina | 2006 |
| 8LKN0206 | JN671155 | Argentina | 2006 |
| 8LLN0206 | JN671156 | Argentina | 2006 |
| 8LMN0206 | JN671160 | Argentina | 2006 |
| 8LNN0206 | JN671162 | Argentina | 2006 |
| 8LON0206 | JN671167 | Argentina | 2006 |
| 8LPN0206 | JN671169 | Argentina | 2006 |
| 8LQN0206 | JN671171 | Argentina | 2006 |
| 8LRN0206 | JN671173 | Argentina | 2006 |
| 8LSN0206 | JN671174 | Argentina | 2006 |
| 8LTN0206 | JN671176 | Argentina | 2006 |
| 8LUN0206 | JN671179 | Argentina | 2006 |
| 8LVN0206 | JN671181 | Argentina | 2006 |
| 8LWN0206 | JN671182 | Argentina | 2006 |
| 8LXN0206 | JN671183 | Argentina | 2006 |
| 8LYN0206 | JN671188 | Argentina | 2006 |
| 8LZN0206 | JN671189 | Argentina | 2006 |
| 8MBN0206 | JN671192 | Argentina | 2006 |
| 8MCN0206 | JN671193 | Argentina | 2006 |
| 8MDN0206 | JN671194 | Argentina | 2006 |
| 8MFN0206 | JN671197 | Argentina | 2006 |
| 8MGN0206 | JN671199 | Argentina | 2006 |
| 8MHN0206 | JN671209 | Argentina | 2006 |
| 8MJN0206 | JN671213 | Argentina | 2006 |
| 8MKN0206 | JN671215 | Argentina | 2006 |
| 8MLN0206 | JN671218 | Argentina | 2006 |
| 8MMN0206 | JN671222 | Argentina | 2006 |
| 8MNN0206 | JN671223 | Argentina | 2006 |
| 8MON0206 | JN671226 | Argentina | 2006 |
| 8MPN0206 | JN671227 | Argentina | 2006 |
| 8MQN0206 | JN671229 | Argentina | 2006 |
| 8MRN0206 | JN671230 | Argentina | 2006 |
| 8MSN0206 | JN671233 | Argentina | 2006 |
| 8MTN0206 | JN671234 | Argentina | 2006 |
| 8MUN0206 | JN671237 | Argentina | 2006 |
| 8MVN0206 | JN671238 | Argentina | 2006 |
| 8MWN0206 | JN671239 | Argentina | 2006 |
| 8MXN0206 | JN671241 | Argentina | 2006 |
| 8MYN0206 | JN671242 | Argentina | 2006 |
| 8MZN0206 | JN671243 | Argentina | 2006 |
| 8NAN0206 | JN671245 | Argentina | 2006 |
| 8NBN0206 | JN671246 | Argentina | 2006 |
| 8NCN0207 | JN671247 | Argentina | 2007 |
| 8NDN0207 | JN671249 | Argentina | 2007 |
| 8NEN0207 | JN671250 | Argentina | 2007 |
| 8NFN0207 | jN671253 | Argentina | 2007 |
| 8NGN0207 | JN671256 | Argentina | 2007 |
| 8NHN0207 | JN671257 | Argentina | 2007 |
| 8NIN0207 | JN671259 | Argentina | 2007 |
| 8NJN0207 | JN671260 | Argentina | 2007 |
| 8NKN0207 | JN671261 | Argentina | 2007 |
| 8NLN0207 | JN671262 | Argentina | 2007 |
| 8NMN0207 | JN671264 | Argentina | 2007 |
| 8NON0207 | JN671270 | Argentina | 2007 |
| 8NPN0207 | JN671271 | Argentina | 2007 |
| 8NRN0207 | JN671274 | Argentina | 2007 |
| 8NSN0207 | JN671275 | Argentina | 2007 |
| 8NTN0207 | JN671276 | Argentina | 2007 |
| 8NUN0207 | JN671280 | Argentina | 2007 |
| 8NVN0207 | JN671282 | Argentina | 2007 |
| 8NWN0207 | JN671288 | Argentina | 2007 |
| 8NYN0207 | JN671291 | Argentina | 2007 |
| 8NZN0207 | JN671294 | Argentina | 2007 |
| 8OAN0207 | JN671297 | Argentina | 2007 |
| 8OBN0207 | JN671298 | Argentina | 2007 |
| 8ODN0207 | JN671301 | Argentina | 2007 |
| 8OFN0207 | JN671304 | Argentina | 2007 |
| 8OGN0207 | JN671305 | Argentina | 2007 |
| 8OHN0207 | JN671306 | Argentina | 2007 |
| 8OIN0207 | JN671307 | Argentina | 2007 |
| 8OJN0207 | JN671313 | Argentina | 2007 |
| 8OKN0207 | JN671314 | Argentina | 2007 |
| 8OLN0207 | JN671315 | Argentina | 2007 |
| 8OMN0207 | JN671316 | Argentina | 2007 |
| 8ONN0207 | JN671317 | Argentina | 2007 |
| 8OON0207 | JN671320 | Argentina | 2007 |
| 8OPN0207 | JN671322 | Argentina | 2007 |
| 8OQN0207 | JN671333 | Argentina | 2007 |
| 8ORN0207 | JN671345 | Argentina | 2007 |
| 8OTN0207 | JN671348 | Argentina | 2007 |
| 8OUN0207 | JN671351 | Argentina | 2007 |
| 8OVN0207 | JN671353 | Argentina | 2007 |
| 8OWN0207 | JN671354 | Argentina | 2007 |
| 8OXN0207 | JN671355 | Argentina | 2007 |
| 8OYN0207 | JN671356 | Argentina | 2007 |
| 8OZN0207 | JN671357 | Argentina | 2007 |
| 8PAN0207 | JN671360 | Argentina | 2007 |
| 8PBN0207 | JN671365 | Argentina | 2007 |
| 8PCN0207 | JN671367 | Argentina | 2007 |
| 8PDN0207 | JN671368 | Argentina | 2007 |
| 8PEN0207 | JN671369 | Argentina | 2007 |
| 8PFN0207 | JN671370 | Argentina | 2007 |
| 8PGN0207 | JN671375 | Argentina | 2007 |
| 8PHN0207 | JN671376 | Argentina | 2007 |
| 8PIN0207 | JN671377 | Argentina | 2007 |
| 8PKN0207 | JN671379 | Argentina | 2007 |
| 8PLN0207 | JN671380 | Argentina | 2007 |
| 8PMN0207 | JN671381 | Argentina | 2007 |
| 8PNN0207 | JN671385 | Argentina | 2007 |
| 8PON0207 | JN671386 | Argentina | 2007 |
| 8PPN0207 | JN671387 | Argentina | 2007 |
| 8PQN0207 | JN671388 | Argentina | 2007 |
| 8PRN0207 | JN671389 | Argentina | 2007 |
| 8PTN0207 | JN671391 | Argentina | 2007 |
| 8PVN0207 | JN671395 | Argentina | 2007 |
| 8PXN0207 | JN671399 | Argentina | 2007 |
| 8PYN0207 | JN671400 | Argentina | 2007 |
| 8PZN0207 | JN671402 | Argentina | 2007 |
| 8QAN0207 | JN671404 | Argentina | 2007 |
| 8QBN0207 | JN671405 | Argentina | 2007 |
| 8QCN0207 | JN671406 | Argentina | 2007 |
| 8QDN0207 | JN671407 | Argentina | 2007 |
| 8QEN0207 | JN671408 | Argentina | 2007 |
| 8QFN0207 | JN671410 | Argentina | 2007 |
| 8QGN0207 | JN671412 | Argentina | 2007 |
| 8QHN0207 | JN671413 | Argentina | 2007 |
| 8QIN0207 | JN671416 | Argentina | 2007 |
| 8QLN0207 | JN671421 | Argentina | 2007 |
| 8QMN0207 | JN671422 | Argentina | 2007 |
| 8QON0207 | JN671427 | Argentina | 2007 |
| 8QPN0207 | JN671431 | Argentina | 2007 |
| 8QQN0207 | JN671436 | Argentina | 2007 |
| 8QRN0207 | JN671438 | Argentina | 2007 |
| 8QSN0207 | JN671440 | Argentina | 2007 |
| 8QTN0207 | JN671441 | Argentina | 2007 |
| 8RN008N | AY841802 | Venezuela | N |
| 8RO008N | AY841803 | Venezuela | N |
| 8RP008N | AY841804 | Venezuela | N |
| 8RQ008N | AY841805 | Venezuela | N |
| 8RS008N | AY841808 | Venezuela | N |
| 8RT008N | AY841810 | Venezuela | N |
| 8RU008N | AY841811 | Venezuela | N |
| 8RV008N | AY841815 | Venezuela | N |
| 8RW008N | AY841816 | Venezuela | N |
| 8RX008N | AY841817 | Venezuela | N |
| 8RY008N | AY841818 | Venezuela | N |
| 8RZ008N | AY841819 | Venezuela | N |
| 8SB008N | AY841821 | Venezuela | N |
| 8SC008N | AY841822 | Venezuela | N |
| 8SD008N | AY841823 | Venezuela | N |
| 8SG008N | AY841826 | Venezuela | N |
| 8SH008N | AY841827 | Venezuela | N |
| 8SI008N | AY841828 | Venezuela | N |
| 8SJ008N | AY841829 | Venezuela | N |
| 8SK008N | AY841830 | Venezuela | N |
| 8SL00804 | FJ659505 | Venezuela | 2004 |
| 8SM00804 | FJ659506 | Venezuela | 2004 |
| 8SN00804 | FJ659508 | Venezuela | 2004 |
| 8SO00804 | FJ659512 | Venezuela | 2004 |
| 8SQ00804 | FJ659514 | Venezuela | 2004 |
| 8SS00804 | FJ659516 | Venezuela | 2004 |
| 8ST00804 | FJ659517 | Venezuela | 2004 |
| 8SV00804 | FJ659519 | Venezuela | 2004 |
| 8SW00804 | FJ659520 | Venezuela | 2004 |
| 8SX00804 | FJ659521 | Venezuela | 2004 |
| 8SY00804 | FJ659522 | Venezuela | 2004 |
| 8SZ00804 | FJ659523 | Venezuela | 2004 |
| 8TA00804 | FJ659524 | Venezuela | 2004 |
| 8TB00804 | FJ659525 | Venezuela | 2004 |
| 8TC00804 | FJ659526 | Venezuela | 2004 |
| 8TF00804 | FJ659529 | Venezuela | 2004 |
| 8TG00804 | FJ659530 | Venezuela | 2004 |
| 8TH00804 | FJ659531 | Venezuela | 2004 |
| 8TI00804 | FJ659532 | Venezuela | 2004 |
| 8TJ00804 | FJ659533 | Venezuela | 2004 |
| 8TK00804 | FJ659534 | Venezuela | 2004 |
| 8TM00804 | FJ659536 | Venezuela | 2004 |
| 8TN00804 | FJ659537 | Venezuela | 2004 |
| 8TP00804 | FJ659539 | Venezuela | 2004 |
| 8TQ00804 | FJ659540 | Venezuela | 2004 |
| 8TR00804 | FJ659541 | Venezuela | 2004 |
| 8TS00804 | FJ659542 | Venezuela | 2004 |
| 8TT00804 | FJ659543 | Venezuela | 2004 |
| 8TU00804 | FJ659545 | Venezuela | 2004 |
| 8TW00804 | FJ659547 | Venezuela | 2004 |
| 8TX00804 | FJ659548 | Venezuela | 2004 |
| 8TY00804 | FJ659549 | Venezuela | 2004 |
| 8TZ00804 | FJ659550 | Venezuela | 2004 |
| 8UA00804 | FJ659551 | Venezuela | 2004 |
| 8UC00804 | FJ659553 | Venezuela | 2004 |
| 8UD00804 | FJ659554 | Venezuela | 2004 |
| 8UF00804 | FJ659556 | Venezuela | 2004 |
| 8UJ00804 | FJ659560 | Venezuela | 2004 |
| 8UK00804 | FJ659561 | Venezuela | 2004 |
| 8UM00804 | FJ659563 | Venezuela | 2004 |
| 8UN00804 | FJ659564 | Venezuela | 2004 |
| 8UO00804 | FJ659565 | Venezuela | 2004 |
| 8UP00804 | FJ659566 | Venezuela | 2004 |
| 8UQ00804 | FJ659567 | Venezuela | 2004 |
| 8UR00804 | FJ659568 | Venezuela | 2004 |
| 8UT00804 | FJ659570 | Venezuela | 2004 |
| 8UU00804 | FJ659571 | Venezuela | 2004 |
| 8UV00804 | FJ659572 | Venezuela | 2004 |
| 8UW00804 | FJ659573 | Venezuela | 2004 |
| 8UX00804 | FJ659574 | Venezuela | 2004 |
| 8UY00804 | FJ659575 | Venezuela | 2004 |
| 8UZ00804 | FJ659576 | Venezuela | 2004 |
| 8WA00804 | FJ659577 | Venezuela | 2004 |
| 8WB00804 | FJ659578 | Venezuela | 2004 |
| 8WC00804 | FJ659579 | Venezuela | 2004 |
| 8WD00804 | FJ659580 | Venezuela | 2004 |
| 8WE00804 | FJ659581 | Venezuela | 2004 |
| 8WF00804 | FJ659582 | Venezuela | 2004 |
| 8WG00804 | FJ659583 | Venezuela | 2004 |
| 8WH00804 | FJ659584 | Venezuela | 2004 |
| 8WI00804 | FJ659585 | Venezuela | 2004 |
| 8WJ00804 | FJ659586 | Venezuela | 2004 |
| 8WN00804 | FJ659590 | Venezuela | 2004 |
| 8WO00804 | FJ659591 | Venezuela | 2004 |
| 8WP00804 | FJ659592 | Venezuela | 2004 |
| 8WQ00804 | FJ659593 | Venezuela | 2004 |
| 8WR00804 | FJ659594 | Venezuela | 2004 |
| 8WS00804 | FJ659595 | Venezuela | 2004 |
| 8WT00804 | FJ659596 | Venezuela | 2004 |
| 8WU00804 | FJ659597 | Venezuela | 2004 |
| 8WV00804 | FJ659598 | Venezuela | 2004 |
| 8WW00804 | FJ659599 | Venezuela | 2004 |
| 8WY00804 | FJ659601 | Venezuela | 2004 |
| 8WZ00804 | FJ659602 | Venezuela | 2004 |
| 8XA00804 | FJ659603 | Venezuela | 2004 |
| 8XB00804 | FJ659604 | Venezuela | 2004 |
| 8XC00804 | FJ659605 | Venezuela | 2004 |
| 8XE00804 | FJ659607 | Venezuela | 2004 |
| 8XF00804 | FJ659608 | Venezuela | 2004 |
| 8XH00804 | FJ659610 | Venezuela | 2004 |
| 8XI00804 | FJ659611 | Venezuela | 2004 |
| 8XK00804 | FJ659613 | Venezuela | 2004 |
| 8XL00804 | FJ659614 | Venezuela | 2004 |
| 8XM00804 | FJ659615 | Venezuela | 2004 |
| 8XN00804 | FJ659616 | Venezuela | 2004 |
| 8XO00804 | FJ659617 | Venezuela | 2004 |
| 8XP00804 | FJ659618 | Venezuela | 2004 |
| 8XQ00804 | FJ659619 | Venezuela | 2004 |
| 8XR00804 | FJ659620 | Venezuela | 2004 |
| 8XS00804 | FJ659621 | Venezuela | 2004 |
| 8XU00804 | FJ659623 | Venezuela | 2004 |
| 8XV00804 | FJ659624 | Venezuela | 2004 |
| 8XW00804 | FJ659625 | Venezuela | 2004 |
| 8XY00804 | FJ659627 | Venezuela | 2004 |
| 8XZ00804 | FJ659628 | Venezuela | 2004 |
| 8YA00804 | FJ659629 | Venezuela | 2004 |
| 8YB00804 | FJ659630 | Venezuela | 2004 |
| 8YC00804 | FJ659631 | Venezuela | 2004 |
| 8YE00804 | FJ659633 | Venezuela | 2004 |
| 8YF00804 | FJ659634 | Venezuela | 2004 |
| 8YG00804 | FJ659635 | Venezuela | 2004 |
| 8YH00804 | FJ659636 | Venezuela | 2004 |
| 8YI00804 | FJ659637 | Venezuela | 2004 |
| 8YJ00804 | FJ659638 | Venezuela | 2004 |
| 8YK00804 | FJ659640 | Venezuela | 2004 |
| 8YL00804 | FJ659641 | Venezuela | 2004 |
| 8YM00804 | FJ659643 | Venezuela | 2004 |
| 8YN00804 | FJ659644 | Venezuela | 2004 |
| 8YO00804 | FJ659645 | Venezuela | 2004 |
| 8YP00804 | FJ659646 | Venezuela | 2004 |
| 8YQ00804 | FJ659647 | Venezuela | 2004 |
| 8YR00804 | FJ659648 | Venezuela | 2004 |
| 8YS00804 | FJ659649 | Venezuela | 2004 |
| 8YT00804 | FJ659650 | Venezuela | 2004 |
| 8YU00804 | FJ659651 | Venezuela | 2004 |
| 8YW00804 | FJ659653 | Venezuela | 2004 |
| 8YY00804 | FJ659655 | Venezuela | 2004 |
| 8YZ00804 | FJ659656 | Venezuela | 2004 |
| 8ZA00804 | FJ659657 | Venezuela | 2004 |
| 8ZB00804 | FJ659658 | Venezuela | 2004 |
| 8ZC00804 | FJ659659 | Venezuela | 2004 |
| 8ZD00804 | FJ659660 | Venezuela | 2004 |
| 8ZE00804 | FJ659661 | Venezuela | 2004 |
| 8ZG00804 | FJ659663 | Venezuela | 2004 |
| 8ZH00804 | FJ659664 | Venezuela | 2004 |
| 8ZI00804 | FJ659665 | Venezuela | 2004 |
| 8ZJ00804 | FJ659666 | Venezuela | 2004 |
| 8ZK00804 | FJ659667 | Venezuela | 2004 |
| 8ZL00804 | FJ659668 | Venezuela | 2004 |
| 8ZO00804 | FJ659671 | Venezuela | 2004 |
| 8ZP00804 | FJ659672 | Venezuela | 2004 |
| 8ZS00804 | FJ659676 | Venezuela | 2004 |
| 8ZW00804 | FJ659703 | Venezuela | 2004 |
| 80A00805 | GU807494 | Venezuela | 2005 |
| 80B00805 | GU807495 | Venezuela | 2005 |
| 80C00809 | GU807496 | Venezuela | 2009 |
| 80D00809 | GU807497 | Venezuela | 2009 |
| 80E00809 | GU807498 | Venezuela | 2009 |
| 80F00809 | GU807499 | Venezuela | 2009 |
| 80G00809 | GU807500 | Venezuela | 2009 |
| 80H00809 | GU807501 | Venezuela | 2009 |
| 80L00809 | GU807505 | Venezuela | 2009 |
| 80M00809 | GU807506 | Venezuela | 2009 |
| 80O00809 | GU807508 | Venezuela | 2009 |
| 80P00809 | GU807509 | Venezuela | 2009 |
| 80Q00809 | GU807510 | Venezuela | 2009 |
| 80R00809 | GU807511 | Venezuela | 2009 |
| 80S00809 | GU807512 | Venezuela | 2009 |
| 80T00809 | GU807513 | Venezuela | 2009 |
| 80U00809 | GU807514 | Venezuela | 2009 |
| 80V00809 | GU807515 | Venezuela | 2009 |
| 80W00809 | GU807516 | Venezuela | 2009 |
| 80X00809 | GU807517 | Venezuela | 2009 |
| 80Y00809 | GU807518 | Venezuela | 2009 |
| 81A00809 | GU807520 | Venezuela | 2009 |
| 81B00809 | GU807521 | Venezuela | 2009 |
| 81D00809 | GU807523 | Venezuela | 2009 |
| 81E00809 | GU807524 | Venezuela | 2009 |
| 81F00809 | GU807525 | Venezuela | 2009 |
| 81G00809 | GU807526 | Venezuela | 2009 |
| 81I00809 | GU807528 | Venezuela | 2009 |
| 81J00809 | GU807529 | Venezuela | 2009 |
| 81T10706 | JF320009 | Peru | 2006 |
| 81V10707 | JF320012 | Peru | 2007 |
| 81W10707 | JF320013 | Peru | 2007 |
| 81X10707 | JF320014 | Peru | 2007 |
| 81Y10707 | JF320015 | Peru | 2007 |
| 81Z10707 | JF320016 | Peru | 2007 |
| 82A10707 | JF320017 | Peru | 2007 |
| 82B10707 | JF320018 | Peru | 2007 |
| 82C10707 | JF320019 | Peru | 2007 |
| 82D10707 | JF320020 | Peru | 2007 |
| 82E10707 | JF320021 | Peru | 2007 |
| 82F10707 | JF320022 | Peru | 2007 |
| 82H10707 | JF320024 | Peru | 2007 |
| 82I10707 | JF320025 | Peru | 2007 |
| 82J10707 | JF320026 | Peru | 2007 |
| 82L10707 | JF320028 | Peru | 2007 |
| 82M10707 | JF320029 | Peru | 2007 |
| 82N10707 | JF320030 | Peru | 2007 |
| 82P10707 | JF320032 | Peru | 2007 |
| 82S10707 | JF320035 | Peru | 2007 |
| 82T10706 | JF320183 | Peru | 2006 |
| 82U10706 | JF320186 | Peru | 2006 |
| 82V10706 | JF320187 | Peru | 2006 |
| 82W10707 | JF320189 | Peru | 2007 |
| 82X10707 | JF320191 | Peru | 2007 |
| 82Y10706 | JF320193 | Peru | 2006 |
| 82Z10707 | JF320195 | Peru | 2007 |
| 83B10707 | JF320198 | Peru | 2007 |
| 83C10707 | JF320199 | Peru | 2007 |
| 83D10706 | JF320201 | Peru | 2006 |
| 83E10706 | JF320203 | Peru | 2006 |
| 83G10707 | JF320206 | Peru | 2007 |
| 83I10706 | JF320209 | Peru | 2006 |
| 83J10707 | JF320211 | Peru | 2007 |
| 83P10707 | JF320217 | Peru | 2007 |
| 83R10706 | JF320219 | Peru | 2006 |
| 83S10707 | JF320220 | Peru | 2007 |
| 83U10706 | JF320222 | Peru | 2006 |
| 83W10706 | JF320224 | Peru | 2006 |
| 84A10706 | JF320228 | Peru | 2006 |
| 84B10706 | JF320229 | Peru | 2006 |
| 84G10706 | JF320241 | Peru | 2006 |
| 84I10706 | JF320243 | Peru | 2006 |
| 84K12601 | AY561236 | Colombia | 2001 |
| 84L12601 | AY561237 | Colombia | 2001 |
| 84M12601 | AY561238 | Colombia | 2001 |
| 84N12601 | AY561239 | Colombia | 2001 |
| 84O12601 | AY561240 | Colombia | 2001 |
| 84P12601 | AY561242 | Colombia | 2001 |
| 84Q11601 | AY561244 | Colombia | 2001 |
| 84R126N | AY566119 | Colombia | N |
| 84S116N | AY566120 | Colombia | N |
| 84T11601 | AY566121 | Colombia | 2001 |
| 84U116N | AY566122 | Colombia | N |
| 87C22302 | JN235958 | Uruguay | 2002 |
| 87D1N399 | JN235959 | Uruguay | 1999 |
| 87E1N399 | JN235965 | Uruguay | 1999 |
| 87I0N000 | AY267326 | Suriname | 2000 |
| 87J0N000 | AY267327 | Suriname | 2000 |
| 87K0N000 | AY267328 | Suriname | 2000 |
| 87L0N189 | AY173959 | Ecuador | 1989 |
| 87M0N189 | AY173960 | Ecuador | 1989 |
| 87N1NQ02 | JN251896 | Paraguay | 2002 |
| 87O2NQ02 | JN251901 | Paraguay | 2002 |
| 87P1NQ03 | JN251906 | Paraguay | 2003 |
| 87R00900 | AY267309 | Guyana | 2000 |
| 87T00900 | AY267311 | Guyana | 2000 |
| 87ZNN201 | AY366043 | Argentina | 2001 |
| 89BNN201 | AY365994 | Argentina | 2001 |
| 9CSNN201 | AY365806 | Argentina | 2001 |
| 9CTNN201 | AY365805 | Argentina | 2001 |
| 9EHNN201 | AY365730 | Argentina | 2001 |
| 9KUNNDN | AY771513 | Brazil | N |
| 9LCNNDN | AY771553 | Brazil | N |
| 9LM00E05 | DQ899677 | Brazil | 2005 |
| 1NC11B12 | EU293463 | Brazil | 2012 |
| 3ND11B12 | FJ591316 | Brazil | 2012 |
| 3NE11B12 | FJ591317 | Brazil | 2012 |
| 6NF11B12 | HM534138 | Brazil | 2012 |
| 6NG11B12 | HM534139 | Brazil | 2012 |
| 6NH22B12 | HM534140 | Brazil | 2012 |
| 6NI22B12 | HM534141 | Brazil | 2012 |
| 6NJ12B12 | HM534142 | Brazil | 2012 |
| 6NK22B12 | HM534143 | Brazil | 2012 |
| 4NL12B12 | HM534145 | Brazil | 2012 |
| 9NM12B13 | HM534146 | Brazil | 2013 |
| 9NN11B13 | HM534147 | Brazil | 2013 |
| 9NO11B13 | HM534148 | Brazil | 2013 |
| 9NP12A11 | HM534149 | Brazil | 2011 |
| 9NR12A12 | HM534151 | Brazil | 2012 |
| 9NS12A12 | HM534152 | Brazil | 2012 |
| 9NT22A12 | HM534153 | Brazil | 2012 |
| 9NU11B12 | HM534154 | Brazil | 2012 |
| 9NW22B12 | HM534156 | Brazil | 2012 |
| 9NX10B12 | HM534157 | Brazil | 2012 |
| 9NY12B12 | HM534158 | Brazil | 2012 |
| 9NZ11B13 | HM534159 | Brazil | 2013 |
| 9OA12A13 | HM534161 | Brazil | 2013 |
| 9OB22A13 | HM534162 | Brazil | 2013 |
| 9OC12A13 | HM534163 | Brazil | 2013 |
| 9OD22A13 | HM534164 | Brazil | 2013 |
| 9OE22A13 | HM534165 | Brazil | 2013 |
| 9OF22A13 | HM534166 | Brazil | 2013 |
| 9OG11A13 | HM534168 | Brazil | 2013 |
| 9OI22A10 | HM534170 | Brazil | 2010 |
| 9OJ22A10 | HM534171 | Brazil | 2010 |
| 9OK12A10 | HM534172 | Brazil | 2010 |
