# Supplementary material for: Short-Term Dynamic and Local Epidemiological Trends in the South American HIV-1B Epidemic
Source: PLoS One. 2016 Jun 3;11(6):e0156712. doi: 10.1371/journal.pone.0156712 (PMC4892525; doi:10.1371/journal.pone.0156712)
Supplement: S2 Table — (DOCX) [file pone.0156712.s003.docx]

**S2 Table. Geographical type of HIV-1 Subtype B transmissions among clusters identified within South America for the complete dataset (1000bp).**

| **Clustered Individuals** | **Geographical Type of Transmission** | **Number of Clusters Identified** | **%** |
| --- | --- | --- | --- |
| **2** | Local Transmission* | 353 | 69.8 |
|  | Interstate Transmission (Brazil) | 40 | 7.91 |
|  | International Transmission | 34 | 6.72 |
|  | Unidentified | 79 | 15.6 |
| **3** | Local Transmission* | 69 | 66.3 |
|  | Interstate Transmission (Brazil) | 13 | 12.5 |
|  | International Transmission | 7 | 6.73 |
|  | Unidentified | 15 | 14.4 |
| **4** | Local Transmission* | 23 | 60.5 |
|  | Interstate Transmission (Brazil) | 9 | 23.7 |
|  | International Transmission | 3 | 7.89 |
|  | Unidentified | 3 | 7.89 |
| **5** | Local Transmission* | 11 | 78.6 |
|  | Interstate Transmission (Brazil) | 1 | 7.14 |
|  | International Transmission | 2 | 14.3 |
| **6** | Local Transmission* | 1 | 100 |
| **7** | Local Transmission* | - | - |
|  | Interstate Transmission (Brazil) | 2 | 100 |
| **≥8** | Local Transmission* | 4 | 100 |
| **Total** | | 669 | - |

* Transmission Clusters involving sequences sampled in the same state for Brazilian sequences or in the same country for non-Brazilian sequences
