# Supplementary material for: Short-Term Dynamic and Local Epidemiological Trends in the South American HIV-1B Epidemic
Source: PLoS One. 2016 Jun 3;11(6):e0156712. doi: 10.1371/journal.pone.0156712 (PMC4892525; doi:10.1371/journal.pone.0156712)
Supplement: S3 Table — (DOCX) [file pone.0156712.s004.docx]

**S3 Table. Number of links identified for the states within Brazil involved in interstate transmissions and for the countries involved in international transmissions at South America (including results from transmission pairs and transmission clusters).**

| **Geographical Type of Transmission** | **Region** | **Number of Links with other Sequences** | |
| --- | --- | --- | --- |
|  |  | **Complete *pol* Sequences** | **Codon-stripped *pol* Sequences** |
| **Interstate** | Amazonas (AM) | - | 1 |
|  | Espírito Santo (ES) | 4 | 2 |
|  | Goiás (GO) | 8 | 5 |
|  | Minas Gerais (MG) | 1 | - |
|  | Mato Grosso do Sul (MS) | 2 | 2 |
|  | Mato Grosso (MT) | 10 | 3 |
|  | Paraná (PR) | 6 | 5 |
|  | Rio de Janeiro (RJ) | 8 | 3 |
|  | Rio Grande do Sul (RS) | 2 | 1 |
|  | Santa Catarina (SC) | 2 | - |
|  | São Paulo (SP) | 12 | 8 |
|  | Tocantins (TO) | 3 | 2 |
|  | Unidentified | 2 | 2 |
| **International** | Argentina | 5 | 6 |
|  | Brazil | 7 | 4 |
|  | Chile | 2 | 2 |
|  | Guiana | 1 | 1 |
|  | Peru | 2 | 2 |
|  | Paraguai | 1 | - |
|  | Uruguai | 2 | 1 |
|  | Venezuela | 2 | 3 |
|  | Unidentified | - | - |
