# Supplementary material for: Short-Term Dynamic and Local Epidemiological Trends in the South American HIV-1B Epidemic
Source: PLoS One. 2016 Jun 3;11(6):e0156712. doi: 10.1371/journal.pone.0156712 (PMC4892525; doi:10.1371/journal.pone.0156712)
Supplement: S4 Table — (DOCX) [file pone.0156712.s005.docx]

**S4 Table. Average time of HIV-1 subtype B transmission among South American individuals for the complete dataset (1000bp).**

| **Cluster** | **Taxa** | **Geographical Type** | **Exposure Category** | **Median Internal Branchs (years)** |
| --- | --- | --- | --- | --- |
| **1** | 5 | Local | MSM | 0.355 |
| **2** | 5 | Local | MSM | 0.468 |
| **3** | 4 | Local | - | 3.615 |
| **4** | 5 | Local | - | 2.350 |
| **5** | 5 | International | - | 6.613 |
| **6** | 5 | Local | MSM | 3.987 |
| **7** | 5 | Local | MSM | 0.273 |
| **8** | 4 | International | - | 0.802 |
| **9** | 5 | Local | HET | 2.355 |
| **10** | 5 | Local | - | 2.617 |
| **11** | 5 | Local | MSM | 0.239 |
| **12** | 6 | Local | MSM | 0.494 |
| **13** | 7 | Interstate | - | 4.035 |
| **14** | 7 | Interstate | HET/MSM | 4.161 |
| **15** | 9 | Local | HET/MSM | 2.973 |
| **16** | 13 | Local | - | 2.891 |
| **Median 2.39 (95% CI 1.48 - 3.30)** | | | | |

Abbreviations: HET: Heterosexual individual, MSM: men who have sex with men individual
