# Supplementary material for: Short-Term Dynamic and Local Epidemiological Trends in the South American HIV-1B Epidemic
Source: PLoS One. 2016 Jun 3;11(6):e0156712. doi: 10.1371/journal.pone.0156712 (PMC4892525; doi:10.1371/journal.pone.0156712)
Supplement: S5 Table — (DOCX) [file pone.0156712.s006.docx]

**S5 Table.** **Amino acid substitutions in HIV-1 Protease gene related to drug resistance to protease inhibitors (PI) identified among 4,810 sequences clustered or not clustered in transmission clusters within South America.**

| **PI Major Mutation** | **Full Dataset (n= 4,810)** | | **Clustered Sequences (n= 1,633)** | | **Not Clustered Sequences (n= 3,177)** | |
| --- | --- | --- | --- | --- | --- | --- |
|  | **N** | **%** | **N** | **%** | **N** | **%** |
| **L90M** | 926 | 17,1 | 214 | 17,5 | 712 | 17 |
| **M46I** | 647 | 11,9 | 159 | 13.0 | 488 | 11,6 |
| **I54V** | 592 | 10,9 | 112 | 9,14 | 480 | 11,4 |
| **V82A** | 556 | 10,3 | 109 | 8,89 | 447 | 10,7 |
| **I84V** | 335 | 6,18 | 111 | 9,05 | 224 | 5,34 |
| **D30N** | 250 | 4,61 | 36 | 2,94 | 214 | 5,1 |
| **N88D** | 233 | 4,3 | 36 | 2,94 | 197 | 4,69 |
| **G73S** | 226 | 4,17 | 60 | 4,89 | 166 | 3,96 |
| **M46L** | 208 | 3,84 | 50 | 4,08 | 158 | 3,77 |
| **I85V** | 160 | 2,95 | 42 | 3,43 | 118 | 2,81 |
| **F53L** | 145 | 2,67 | 29 | 2,37 | 116 | 2,76 |
| **L24I** | 139 | 2,56 | 28 | 2,28 | 111 | 2,65 |
| **V32I** | 138 | 2,55 | 39 | 3,18 | 99 | 2,36 |
| **I47V** | 107 | 1,97 | 32 | 2,61 | 75 | 1,79 |
| **L76V** | 69 | 1,27 | 17 | 1,39 | 52 | 1,24 |
| **I54L** | 62 | 1,14 | 12 | 0,98 | 50 | 1,19 |
| **G73T** | 62 | 1,14 | 13 | 1,06 | 49 | 1,17 |
| **V82T** | 50 | 0,92 | 11 | 0,9 | 39 | 0,93 |
| **V82F** | 50 | 0,92 | 8 | 0,65 | 42 | 1.0 |
| **I50L** | 50 | 0,92 | 3 | 0,24 | 47 | 1,12 |
| **G48V** | 50 | 0,92 | 13 | 1,06 | 37 | 0,88 |
| **I54M** | 47 | 0,87 | 22 | 1,79 | 25 | 0,6 |
| **N88S** | 40 | 0,74 | 11 | 0,9 | 29 | 0,69 |
| **N83D** | 34 | 0,63 | 20 | 1,63 | 14 | 0,33 |
| **I50V** | 28 | 0,52 | 3 | 0,24 | 25 | 0,6 |
| **I54A** | 26 | 0,48 | 3 | 0,24 | 23 | 0,55 |
| **G73C** | 25 | 0,46 | 2 | 0,16 | 23 | 0,55 |
| **L23I** | 24 | 0,44 | 1 | 0,08 | 23 | 0,55 |
| **V82S** | 19 | 0,35 | 6 | 0,49 | 13 | 0,31 |
| **V82C** | 15 | 0,28 | 4 | 0,33 | 11 | 0,26 |
| **G73A** | 11 | 0,2 | 3 | 0,24 | 8 | 0,19 |
| **V82M** | 9 | 0,17 | - | - | 9 | 0,21 |
| **M46IL** | 9 | 0,17 | 1 | 0,08 | 8 | 0,19 |
| **I47A** | 9 | 0,17 | - | - | 9 | 0,21 |
| **F53Y** | 9 | 0,17 | 2 | 0,16 | 7 | 0,17 |
| **V82L** | 8 | 0,15 | 2 | 0,16 | 6 | 0,14 |
| **V82AT** | 8 | 0,15 | 3 | 0,24 | 5 | 0,12 |
| **I54T** | 8 | 0,15 | 3 | 0,24 | 5 | 0,12 |
| **I54S** | 7 | 0,13 | - | - | 7 | 0,17 |
| **G48M** | 7 | 0,13 | 2 | 0,16 | 5 | 0,12 |
| **G73AST** | 5 | 0,09 | - | - | 5 | 0,12 |
| **G73ST** | 4 | 0,07 | 1 | 0,08 | 3 | 0,07 |
| **I54AV** | 3 | 0,06 | 1 | 0,08 | 2 | 0,05 |
| **G73AT** | 3 | 0,06 | - | - | 3 | 0,07 |
| **I54MV** | 2 | 0,04 | 1 | 0,08 | 1 | 0,02 |
| **I54LV** | 2 | 0,04 | - | - | 2 | 0,05 |
| **V82AS** | 1 | 0,02 | - | - | 1 | 0,02 |
| **I84A** | 1 | 0,02 | - | - | 1 | 0,02 |
| **I54ST** | 1 | 0,02 | - | - | 1 | 0,02 |
| **I54LM** | 1 | 0,02 | 1 | 0,08 | - | - |
| **I54AS** | 1 | 0,02 | - | - | 1 | 0,02 |
| **Total** | **5422** | **-** | **1226** | **-** | **4196** | **-** |
