# Supplementary material for: Short-Term Dynamic and Local Epidemiological Trends in the South American HIV-1B Epidemic
Source: PLoS One. 2016 Jun 3;11(6):e0156712. doi: 10.1371/journal.pone.0156712 (PMC4892525; doi:10.1371/journal.pone.0156712)
Supplement: S6 Table — (DOCX) [file pone.0156712.s007.docx]

**S6 Table.** **Amino acid substitutions in HIV-1 Reverse transcriptase gene related to drug resistance to nucleoside reverse transcriptase inhibitors (NRTI) identified among 4,810 sequences clustered or not clustered in transmission clusters within South America.**

| **NRTI Major Mutation** | **Full Dataset (n=4,810)** | | **Clustered Sequences (n= 1,633)** | | **Not Clustered Sequences (n= 3,177)** | |
| --- | --- | --- | --- | --- | --- | --- |
|  | **N** | **%** | **N** | **%** | **N** | **%** |
| M184V | 1831 | 18,2 | 376 | 18,2 | 1455 | 18,2 |
| M41L | 1370 | 13,7 | 306 | 14,8 | 1064 | 13,3 |
| T215Y | 1229 | 12,2 | 268 | 13 | 961 | 12,1 |
| L210W | 899 | 8,96 | 200 | 9,7 | 699 | 8,77 |
| D67N | 1071 | 10,7 | 197 | 9,56 | 874 | 11 |
| K70R | 698 | 6,96 | 118 | 5,73 | 580 | 7,27 |
| L74V | 293 | 2,92 | 67 | 3,25 | 226 | 2,83 |
| T215F | 342 | 3,41 | 61 | 2,96 | 281 | 3,52 |
| K219Q | 354 | 3,53 | 57 | 2,77 | 297 | 3,72 |
| T69D | 209 | 2,08 | 53 | 2,57 | 156 | 1,96 |
| K219E | 298 | 2,97 | 53 | 2,57 | 245 | 3,07 |
| K219R | 87 | 0,87 | 31 | 1,5 | 56 | 0,7 |
| Q151M | 124 | 1,24 | 29 | 1,41 | 95 | 1,19 |
| Y115F | 73 | 0,73 | 28 | 1,36 | 45 | 0,56 |
| F116Y | 98 | 0,98 | 23 | 1,12 | 75 | 0,94 |
| F77L | 101 | 1,01 | 23 | 1,12 | 78 | 0,98 |
| V75M | 140 | 1,4 | 21 | 1,02 | 119 | 1,49 |
| L74I | 142 | 1,42 | 20 | 0,97 | 122 | 1,53 |
| D67G | 79 | 0,79 | 18 | 0,87 | 61 | 0,76 |
| K219N | 134 | 1,34 | 16 | 0,78 | 118 | 1,48 |
| K65R | 60 | 0,6 | 11 | 0,53 | 49 | 0,61 |
| T215S | 28 | 0,28 | 9 | 0,44 | 19 | 0,24 |
| T215I | 41 | 0,41 | 9 | 0,44 | 32 | 0,4 |
| V75T | 43 | 0,43 | 8 | 0,39 | 35 | 0,44 |
| L74IV | 14 | 0,14 | 6 | 0,29 | 8 | 0,1 |
| M184I | 25 | 0,25 | 6 | 0,29 | 19 | 0,24 |
| T215D | 23 | 0,23 | 5 | 0,24 | 18 | 0,23 |
| T215SY | 35 | 0,35 | 5 | 0,24 | 30 | 0,38 |
| V75A | 27 | 0,27 | 5 | 0,24 | 22 | 0,28 |
| T215FY | 26 | 0,26 | 5 | 0,24 | 21 | 0,26 |
| D67E | 13 | 0,13 | 4 | 0,19 | 9 | 0,11 |
| T215V | 17 | 0,17 | 4 | 0,19 | 13 | 0,16 |
| T215CY | 8 | 0,08 | 4 | 0,19 | 4 | 0,05 |
| K70E | 11 | 0,11 | 2 | 0,1 | 9 | 0,11 |
| T215C | 22 | 0,22 | 2 | 0,1 | 20 | 0,25 |
| T215FV | 6 | 0,06 | 2 | 0,1 | 4 | 0,05 |
| T215FIS | 11 | 0,11 | 2 | 0,1 | 9 | 0,11 |
| D67EG | 1 | 0,01 | 1 | 0,05 | 0 | 0 |
| V75S | 8 | 0,08 | 1 | 0,05 | 7 | 0,09 |
| M184IV | 8 | 0,08 | 1 | 0,05 | 7 | 0,09 |
| T215DY | 2 | 0,02 | 1 | 0,05 | 1 | 0,01 |
| T215CF | 2 | 0,02 | 1 | 0,05 | 1 | 0,01 |
| K219QR | 2 | 0,02 | 1 | 0,05 | 1 | 0,01 |
| V75AT | 7 | 0,07 | 1 | 0,05 | 6 | 0,08 |
| T215E | 2 | 0,02 | 0 | 0 | 2 | 0,03 |
| T215FS | 4 | 0,04 | 0 | 0 | 4 | 0,05 |
| K219EQ | 10 | 0,1 | 0 | 0 | 10 | 0,13 |
| T215FI | 6 | 0,06 | 0 | 0 | 6 | 0,08 |
| T215IV | 1 | 0,01 | 0 | 0 | 1 | 0,01 |
| **Total** | **10035** | **-** | **2061** | **-** | **7974** | **-** |
