# Supplementary material for: Short-Term Dynamic and Local Epidemiological Trends in the South American HIV-1B Epidemic
Source: PLoS One. 2016 Jun 3;11(6):e0156712. doi: 10.1371/journal.pone.0156712 (PMC4892525; doi:10.1371/journal.pone.0156712)
Supplement: S7 Table — (DOCX) [file pone.0156712.s008.docx]

**S7 Table.** **Amino acid substitutions in HIV-1 reverse transcriptase gene related to drug resistance to non-nucleoside reverse transcriptase inhibitors (NNRTI) identified among 4,810 sequences clustered or not clustered in transmission clusters within South America.**

| **NNRTI Major Mutation** | **Full Dataset (n=4,810)** | | **Clustered Sequences (n= 1,633)** | | **Not Clustered Sequences (n= 3,177)** | |
| --- | --- | --- | --- | --- | --- | --- |
|  | **N** | **%** | **N** | **%** | **N** | **%** |
| **190A** | 13 | 0,42 | - | - | 13 | 0,54 |
| **190S** | 2 | 0,07 | - | - | 2 | 0,08 |
| **225H** | 3 | 0.10 | - | - | 3 | 0,12 |
| **230L** | 1 | 0,03 | - | - | 1 | 0,04 |
| **G190A** | 450 | 14,7 | 105 | 16.0 | 345 | 14,3 |
| **G190AS** | 2 | 0,07 | - | - | 2 | 0,08 |
| **G190E** | 13 | 0,42 | 5 | 0,76 | 8 | 0,33 |
| **G190S** | 75 | 2,44 | 16 | 2,44 | 59 | 2,45 |
| **K101E** | 205 | 6,68 | 37 | 5,63 | 168 | 6,96 |
| **K101P** | 57 | 1,86 | 11 | 1,67 | 46 | 1,91 |
| **K103N** | 1095 | 35,7 | 244 | 37,1 | 851 | 35,3 |
| **K103NS** | 18 | 0,59 | 3 | 0,46 | 15 | 0,62 |
| **K103S** | 58 | 1,89 | 8 | 1,22 | 50 | 2,07 |
| **L100I** | 165 | 5,37 | 30 | 4,57 | 135 | 5,59 |
| **M230L** | 32 | 1,04 | 3 | 0,46 | 29 | 1.20 |
| **P225H** | 146 | 4,76 | 25 | 3,81 | 121 | 5,01 |
| **V106A** | 47 | 1,53 | 7 | 1,07 | 40 | 1,66 |
| **V106M** | 31 | 1,01 | 6 | 0,91 | 25 | 1,04 |
| **V179F** | 4 | 0,13 | - | - | 4 | 0,17 |
| **Y181C** | 482 | 15,7 | 115 | 17,5 | 367 | 15,2 |
| **Y181I** | 21 | 0,68 | 7 | 1,07 | 14 | 0,58 |
| **Y181V** | 14 | 0,46 | 3 | 0,46 | 11 | 0,46 |
| **Y188C** | 5 | 0,16 | - | - | 5 | 0,21 |
| **Y188H** | 7 | 0,23 | 2 | 0.30 | 5 | 0,21 |
| **Y188HL** | 7 | 0,23 | 2 | 0.30 | 5 | 0,21 |
| **Y188L** | 117 | 3,81 | 28 | 4,26 | 89 | 3,69 |
| **Total** | **3070** | **-** | **657** | **-** | **2413** | **-** |
