# Supplementary material for: Short-Term Dynamic and Local Epidemiological Trends in the South American HIV-1B Epidemic
Source: PLoS One. 2016 Jun 3;11(6):e0156712. doi: 10.1371/journal.pone.0156712 (PMC4892525; doi:10.1371/journal.pone.0156712)
Supplement: S8 Table — (DOCX) [file pone.0156712.s009.docx]

**S8 Table.** **Antiretroviral therapy (ART) status of the patients and clustering behavior of the respective sequences.**

| **Patients' Antiretroviral Therapy Status** | **Full Dataset (n=4,810)** | | **Clustered Sequences (n=1,633)** | | **Not Clustered Sequences (n=3,177)** | |
| --- | --- | --- | --- | --- | --- | --- |
|  | **N** | **%** | **N** | **%** | **N** | **%** |
| Naive | 511 | 10.6 | 275* | 16.8 | 236 | 7.4 |
| Treated | 221 | 4.6 | 88 | 5.4 | 133 | 4.2 |
| Failing ART | 1777 | 36.9 | 506* | 31 | 1271 | 40 |
| Unidentified | 2301 | 47.8 | 764 | 46.8 | 1537 | 48.4 |
| **Total** | **4810** | **-** | **1633** | **-** | **3177** | **-** |

*P<0.001
