# Supplementary material for: Short-Term Dynamic and Local Epidemiological Trends in the South American HIV-1B Epidemic
Source: PLoS One. 2016 Jun 3;11(6):e0156712. doi: 10.1371/journal.pone.0156712 (PMC4892525; doi:10.1371/journal.pone.0156712)
Supplement: S9 Table — (DOCX) [file pone.0156712.s010.docx]

**S9 Table.** **Ancestral reconstruction analysis of the sequences included in clusters and presenting resistance drug mutations.**

| **Cluster** | **Number of individuals** | **Region** | **Type** |  | **Resistance Mutations Analyzed** | | | | |  | **Ancestral Reconstruction** | |
| --- | --- | --- | --- | --- | --- | --- | --- | --- | --- | --- | --- | --- |
|  |  |  |  |  | **PI** |  | **NRTI** |  | **NNRTI** |  | **PT** | **RT** |
| **33** | 3 | Chile | Local |  | - |  | - |  | K103N |  | - | K103N |
| **267** | 4 | Venezuela | Local |  | M46I, I84V, L90M |  | D67N, T215F, K219Q |  | K101P, K103N |  | M46I, I84V, L90M | D67N, T215F, K219Q |
| **305** | 5 | Argentina | Local |  | - |  | M184V |  | K103NS |  | - | K103N |
| **380** | 3 | Argentina | Local |  | - |  | D67N, L74IV, M184V, L210W, T215Y |  | Y181C |  | - | D67N, Y181C, M184V, L210W, T215Y |
| **445** | 4 | Argentina | Local |  | - |  | M41L, T215F |  | - |  | - | M41L, T215F |
| **469** | 24 | Argentina | Local |  | M46I, I84V, L90M |  | M41L, D67N, M184V, L210W, T215Y |  | Y188L |  | M46I, I84V, L90M | M41L, D67N |
| **476** | 4 | São Paulo | Local |  | - |  | - |  | K103N |  | - | K103N |
| **507** | 3 | Argentina | Local |  | V32I, M46I, I47V, I54M, V82A, L90M |  | K65R, K70R, Q151M, K219E |  | Y181C |  | V32I, M46I, I47V, I54M, V82A, L90M | K65R, K70R, Q151M, Y181C, K219E |
| **508** | 3 | Venezuela | Local |  | I84V, L90M |  | Y115F, Q151M, M184V |  | - |  | I84V, L90M | Y115F, Q151M, M184V |
| **510** | 3 | Argentina | Local |  | L90M |  | M41L, L210W, T215Y |  | G190A |  | L90M | M41L, L90M, L210W, T215Y |
| **511** | 3 | Argentina | Local |  | M46L, V82A, L90M |  | M41L, L210W, T215Y |  | - |  | M46L, V82A, L90M | M41L, L210W, T215Y |
| **526** | 4 | Argentina | Local |  | L90M |  | - |  | K103N |  | - | K103N |
| **552** | 3 | Argentina | Local |  | - |  | M41L, D67N, T69D, K70R, L210W, T215Y, K219Q |  | - |  | - | M41L, D67N, T69D, K70R, L210W, T215Y, K219Q |
| **560** | 3 | Argentina | Local |  | M46I, L90M |  | T215Y |  | - |  | M46I, L90M | T215Y |
| **575** | 3 | Argentina | Local |  | - |  | T215SY |  | - |  | - | T215Y |
| **578** | 3 | Argentina | Local |  | M46L, I54V, V82A |  | M41L, D67N, T215Y |  | K103N, G190A |  | M46L, I54V, V82A | M41L, D67N, K103S, G190A, T215Y |
| **632** | 3 | Argentina | Local |  | G48V, I54V, V82A, L90M |  | M41L, T215FY |  | - |  | G48V, I54V, V82A, L90M | M41L, T215FY |
| **642** | 3 | Argentina | Local |  | M46L, L90M |  | M41L, L74V, L210W, T215Y |  | - |  | M46L, L90M | M41L, L74V, L210W, T215Y |
| **643** | 20 | Argentina | Local |  | M46I, I84V |  | M41L, T69D, M184V, T215Y, K219R |  | K103N |  | - | M41L, T69D, T215Y |
| **648** | 3 | Argentina | Local |  | I84V, L90M |  | M41L, D67G, T69D, L210W, T215Y, K219R |  | Y181C |  | I84V, L90M | M41L, D67G, T69D, Y181C, L210W, T215Y, K219R |
| **653** | 3 | Argentina | Local |  | M46I, I54V, V82A, L90M |  | M41L, L210W, T215Y |  | K103N |  | M46I, I54V, L90M | M41L, K103N, L210W, T215Y |
| **658** | 3 | Argentina | Local |  | M46L, G48V, I50V, I54V, V82A |  | M41L, K70R, T215FY, K219E |  | Y181C, G190A |  | M46L, G48V, I50V, I54V, V82A | M41L, K70R, T215F, K219E |
| **665** | 3 | Argentina | Local |  | M46I, I47V, I54V, I84V, L90M |  | L74I, T215Y |  | K103N |  | M46I, I47V, I54V, I84V, L90M | L74I, K103N, T215Y |
| **666** | 3 | Argentina | Local |  | M46L, I47V, I54M, L76V, I84V, I85V, L90M |  | M41L, D67N, T69D, L210W, T215Y, K219N |  | L100I, K103N |  | M46L, I47V, I54M, L76V, I84V, I85V, L90M | M41L, D67N, T69D, L100I, K103N, L210W, T215Y, K219N |
| **668** | 3 | Argentina | Local |  | M46L, I54V, V82A, L90M |  | M41L, D67N, L74V, L210W, T215Y, K219R |  | 101E, Y181C, G190A |  | M46L, I54V, V82A, L90M | M41L, D67N, L74V, 101E, Y181C, G190A, L210W, T215Y, K219R |
| **156** | 3 | Argentina | Local |  | - |  | M41L, D67N, K70R, T215F, K219E |  | - |  | - | M41L, D67N, K70R, T215F, K219E |
| **184** | 3 | Brasil | Interestate |  | D30N, N88D |  | - |  | - |  | D30N, N88D | - |
| **206** | 4 | Argentina | Local |  | V32I, M46I, V82A |  | D67G, T69D, K70R, M184V, T215F, K219Q |  | - |  | V32I, M46L, V82A | D67G, T69D, K70R, M184V, T215F, K219Q |

*Drug resistance mutations harbored by the reconstructed ancestral sequence which were detected in the sequences included in the cluster
